# Comparative Enzymatic and Stability Assays Reveal GPLG as an Effective Cathepsin B Cleavable Linker for Tumor-Targeting Drug Conjugates

**DOI:** 10.1021/acsomega.5c05758

**Published:** 2025-09-04

**Authors:** Giulia Cazzaniga, Marco Zambra, Samuele Bongiolo, Helena Prpić, Elettra Fasola, Federico Arrigoni, Umberto Piarulli, Silvia Gazzola

**Affiliations:** Department of Science and High Technology, University of Insubria, via Valleggio 9, Como 22100, Italy

## Abstract

In the past decade, targeted drug delivery systems have significantly advanced cancer therapy. A key component of these constructs is the chemical linker that covalently connects a targeting unit to a potent cytotoxic payload. Among approved and investigational antibody–drug conjugates (ADCs) and small molecule–drug conjugates (SMDCs), lysosomal-cleavable peptide sequences such as Val-Cit (VCit), Val–Ala (VA), and Gly–Phe–Leu–Gly (GFLG) are widely used for tumor-specific drug release. However, premature drug release and instability often cause off-target toxicity and poor selectivity. Since lysosomal proteases are still considered optimal for the drug release within the tumor site, the quest for new and more stable lysosomal-sensitive peptide sequences is currently an ongoing challenge. This work investigates the enzymatic susceptibility, cleavage kinetics, and metabolic stability of the peptide sequence Gly–Pro–Leu–Gly (GPLG) as a novel Cathepsin B-cleavable linker for tumor-targeting drug conjugates. Compared to GFLG, VCit, and VA, all conjugated to paclitaxel via a PABC-*N*,*N*′-dimethylethylenediamine spacer, GPLG exhibited the fastest Cathepsin B cleavage within the first 30 min of the assay, and higher stability at pH 5.4 and in both human and rat plasma samples. These results highlight GPLG as a promising lysosomal-sensitive linker for next-generation SMDCs and ADCs.

## Graphical abstract

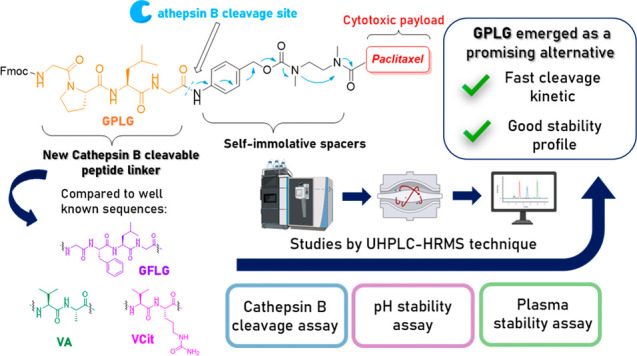

## Introduction

The design of ligand-targeted therapy, exemplified by ligand–drug conjugates, is nowadays an established approach for cancer treatment.^1^ Ideally, linking cytotoxic molecules to ligands (small molecules, peptides, or antibodies) specific for tumor cell membrane receptors aims at ensuring the selective delivery of the cytotoxic payload to cancer cells thanks to the presence of the targeting unit, thereby circumventing side effects due to off-targeting and consequent damage of healthy tissues.^2^

The design of antibody–drug conjugates (ADCs), small molecule–drug conjugates (SMDCs), and peptide–drug conjugates (PDCs) follows a similar principle, which is based on the assembly of the triad composed of (i) a targeting ligand, (ii) a cytotoxic payload, and (iii) a linker connecting the other two subunits. A careful design of this last component is very important to tune the hydrophobicity of the conjugate (e.g., adding a PEG spacer or short hydrophilic peptide sequences) as well as to ensure enough stability for systemic circulation while, at the same time, allowing the fast release of the cytotoxic payload after internalization of the construct inside the targeted cells.^3^ In this context, cleavable linkers, classified according to the stimulus triggering the payload release, play a central role in the clinical success of drug-conjugates. Linkers sensitive to acidic hydrolysis, such as hydrazone-containing linkers, and those responsive to reductive conditions, such as disulfide bridge linkers, take advantage of the environmental conditions of cellular compartments encountered after the conjugate is internalized. Specifically, the lower pH in lysosomes (around 4.6 compared to the neutral pH of 7.4 in human plasma)^4^ and the reductive environment in cancer cells, characterized by higher glutathione levels,^5^ enhance the effectiveness of these linkers. An alternative drug release strategy involves the use of protease-cleavable linkers, as demonstrated by the clinical success of several ADCs.^6^ This approach is advantageous when the protease that selectively recognizes the cleavable linker is specifically overexpressed at the target site; otherwise, premature drug release may occur. Significant attention has been gained by the lysosomal cysteine protease Cathepsin B due to its selective overexpression in tumor tissues.^7,8^ Furthermore, Cathepsin B isolated from many mammalian tissues shows no notable differences between species,^9^ enabling *in vivo* preclinical studies of prodrugs triggered by this lysosomal protease. Interestingly, these distinctive features have attracted the interest of the scientific community beyond ADCs, and many Cathepsin B-recognized peptide sequences have been included in a wide number of PDCs, SMDCs, prodrugs, and cancer-imaging agents.^10,11^ The Val-Cititrulline (VCit) dipeptide is the most established Cathepsin B-linker, recurring in 6 out of 9 FDA-approved cleavable ADCs.^12^

In addition to VCit,^11^ other dipeptide and tetrapeptide sequences have been commonly used in drug-conjugates, and the Val–Ala (VA)^13,14^ dipeptide, the Gly–Gly–Phe–Gly (GGFG),^15^ and the Gly–Phe–Leu–Gly (GFLG)^16^ tetrapeptide sequences are some of the most representative. For example, the VA linker was successfully inserted in loncastuximab tesirine, a recently approved ADC for the treatment of diffuse large B cell lymphoma,^17^ and is contained in other ADCs and SMDCs under preclinical and clinical development.^18–20^ Similarly, the tetrapeptide GGFG^10,15,21^ has been employed in several ADCs, including the clinically approved trastuzumab deruxtecan (Enhertu),^15^ but its use in smaller types of drug-conjugates has been less explored.^22^ Although all of these examples demonstrate the clinical success of conjugates bearing the Cathepsin B cleavable sequences, some limitations related to their application in the field have been highlighted. For instance, it has been reported that patients treated with VCit-based ADCs can experience off-target toxicities leading in some cases to neutropenia,^23^ probably due to a premature drug release from the conjugate mediated by the serine protease elastase.^24^ On the other hand, the use of the GFLG sequence as a cleavable linker revealed too slow drug release within the tumor environment.^25^

Thus, research efforts are currently focused on identifying new lysosomal cleavable linkers with superior performance in terms of kinetic drug release and of stability in both rat and human plasma.^26–28^ Indeed, a deep chemical characterization of the linker behavior in early *in vitro* studies can significantly help the development process of novel stable drug conjugates by minimizing the need for extensive *in vivo* studies while offering valuable insights to guide structural modifications and support the interpretation of both *in vitro* and *in vivo* efficacy data.

In a recent work, we reported the synthesis and biological evaluation of an SMDC containing the potent antimitotic agent monomethyl auristatin E (MMAE)^29^ and a cyclic peptidomimetic^30^ α_v_β_3_ integrin receptor ligand, connected through a novel lysosomally cleavable sequence, Gly–Pro–Leu–Gly (GPLG)^31^ bound to the self-immolative linker *p*-aminobenzyl alcohol (PABOH). Our findings demonstrated higher antitumor efficacy of the GPLG-containing SMDC with respect to the corresponding VA-based SMDC against U87MG, a brain tumor cell line overexpressing the α_v_β_3_ integrin receptor.^32^ Additionally, by using a short-contact time antiproliferative assay to simulate the rapid clearance of the drugs that occurs *in vivo* in the extracellular tumor environment,^33,34^ the GPLG-conjugate showed to be 3 times more potent than the free drug (IC_50_ values against U87MG cancer cell lines of 2.13 μM for MMAE, and 0.68 μM for GPLG-based MMAE conjugate). A final enzymatic cleavage assay demonstrated the ability of the conjugate to release free MMAE when incubated with rat liver lysosomal extract, rather than with matrix metalloproteinase-2 (MMP2), which is known to recognize and cleave GPLG-containing peptide sequences.^35,36^

Considering these promising results, this study aims to chemically characterize the GPLG tetrapeptide as a Cathepsin B-cleavable linker and to directly compare its cleavage kinetics and stability profile with those of three reference linkers: Val-Cit (VCit) and Val–Ala (VA), chosen for their widely employment that span across different conjugate classes, and GFLG, selected for its close structural resemblance to GPLG. Indeed, most studies tend to focus on the characterization of individual linkers,^37,38^ without directly testing them under identical experimental conditions, whereas comparative *in vitro* studies evaluating both release kinetics and plasma stability remain surprisingly scarce in the literature.

To specifically assess the contribution of the linker to the overall behavior of the conjugate, we designed and synthesized model compounds consisting solely of the peptide sequence, the self-immolative linker formed by PABC-*N*,*N*′-dimethylethylendiamine spacer, and the cytotoxic payload, without a targeting moiety. Paclitaxel (PTX),^39^ a clinically relevant microtubule stabilizer, was selected as the payload to ensure pharmacological relevance. Furthermore, the PTX bound to the self-immolative linker PABC and the ethylenediamine spacer is a well-established chemical system, previously validated in enzymatic cleavage assays.^40^ Compound **1**, bearing the GPLG-PABC linker, was used to investigate Cathepsin B-mediated cleavage. To validate GPLG as a substrate for Cathepsin B, its cleavage efficiency was compared to that of compounds **2–4**, incorporating GFLG, VCit, and VA sequences, respectively (Figure 1). Cleavage assays were performed using the UHPLC-Orbitrap technology to precisely characterize the fragmentation pathways. In addition, plasma- and pH-dependent stability studies were conducted to further evaluate and compare the robustness of the linkers. These results provide the chemical foundation for the potential application of GPLG as a novel Cathepsin B-sensitive linker in drug conjugates with promising therapeutic profiles.

**Figure 1:**
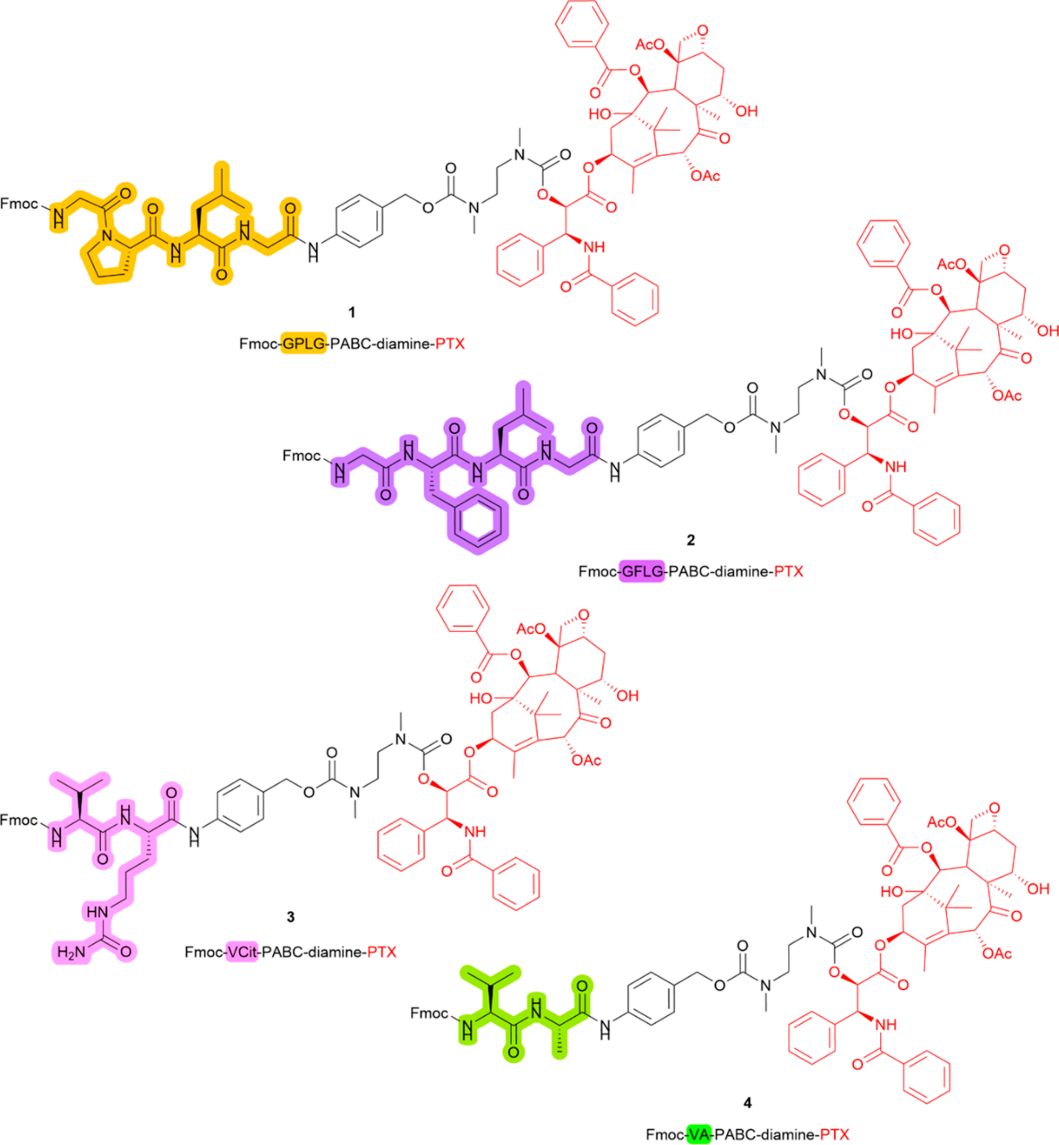
Chemical structures of compounds **1**, **2**, **3**, and **4**. PTX is colored in red, GPLG linker is highlighted in orange, GFLG in purple, VCit linker in pink, and VA linker in green.

## Materials and Methods

### General Information

All the general information on methods and instruments are reported in the Supporting Information

### Solid-Phase Peptide Synthesis: General Procedure

Fmoc-GPLG-OH **5a** and Fmoc-GFLG–OH **5b** were synthesized manually on a commercially available Fmoc-Gly-Wang resin (loading: 0.4–0.8 mmol/g) using the Fmoc protocol according to general procedure A. Fmoc-VCit-OH **5c** was synthesized on a commercially available 2-CTC resin using the Fmoc protocol according to general procedure B. All SPPS syntheses were performed using polypropylene syringes equipped with PTFE frits as reaction vessels; stirring was accomplished by a shaking plate. Dry Fmoc-Gly-Wang and 2-CTC resin were respectively swollen in DMF and DCM for 30 min. 2-CTC resin was then loaded by overnight stirring with a solution of Fmoc-Cit-OH dissolved in DMF/DCM mixture in the presence of DIPEA and subsequently washed using DMF and DCM alternately. Afterward, the capping step was performed by treatment of the loaded 2-CT resin with a DMF/MeOH/DIPEA 17:2:1 mixture for 30 min. Both resins were successively reacted for 3 min (3×) with a 20% piperidine solution in DMF to remove the Fmoc-protecting group. Then, Wang and 2-CT resins were washed by treatment with DMF and IPA alternately and DCM and DMF alternately. Reaction couplings were performed by stirring for 1 h at rt the deprotected resin with a solution of the Fmoc-protected AA previously activated as a reactive ester by treatment of COMU (for couplings on Wang resin) or HATU (for coupling on 2-CT resin) in the presence of DIPEA as the base. Each coupling step was checked through the LC–MS analysis of a small-cleavage sample. The peptide sequences were completed by repetitions of the deprotection-washing-coupling processes, and after a final washing, the final products of SPPS were cleaved from the Wang resin and 2-CT resin by treatment for 1 h (3×) with a TFA/TIS/water 95:2.5:2.5 mixture and for 15 min (3×) with 20% HFIP in DCM, respectively. Details on the complete synthetic procedures are described in the Supporting Information.

#### Fmoc-GPLG-OH (**5a**)

Synthesized by SPPS according to general procedure A. More specifically, 363 mg of Fmoc-Gly-Wang was used. The crude product was precipitated from cold diethyl ether and used in the next step without further purification. A white solid was obtained (69 mg, 0.122 mmol, 49%). *R*_f_ = 0.16 (DCM/MeOH 9:1). ^1^H NMR (400 MHz, methanol-*d*_4_) δ: 7.71 (d, *J* = 7.3 Hz, 2H), 7.59 (d, *J* = 6.9 Hz, 2H), 7.31 (t, *J* = 7.1 Hz, 2H), 7.24 (t, *J* = 7.1 Hz, 2H), 4.43–4.36 (m, 2H), 4.30–4.24 (m, 2H), 4.14 (t, *J* = 6.2 Hz, 1H), 3.92 (dd, *J* = 30.3, 16.8 Hz, 2H), 3.80–3.66 (m, 2H), 3.62–3.58 (m, 1H), 3.52–3.46 (m, 1H), 2.29–2.07 (m, 1H), 2.01–1.78 (m, 3H), 1.68–1.61 (m, 3H), 0.88 (d, *J* = 4.7 Hz, 3H), 0.85 (d, *J* = 4.1 Hz, 3H). ^13^C NMR (101 MHz, methanol-*d*_4_) δ: 174.73, 174.50, 171.21, 159.04, 158.91, 145.15, 145.07, 144.69, 142.43, 133.01, 132.91, 129.95, 129.82, 128.73, 128.09, 126.17, 120.87, 68.18, 62.32, 53.05, 49.85, 48.19, 47.92, 44.24, 41.02, 30.53, 25.94, 25.75, 23.59, 21.59. MS (ESI^+^) *m*/*z* calculated [C_30_H_37_N_4_O_7_]^+^, 565.2 [M + H]^+^; *m*/*z* obtained, 565.9.

#### Fmoc-GFLG-OH (**5b**)

Synthesized by SPPS according to general procedure A. More specifically, 1.159 g of Fmoc-Gly-Wang was used, and the peptide was precipitated from cold diethyl ether and used in the next step without further purification. A white solid was obtained (341 mg, 0.555 mmol, 80% yield). *R*_f_ = 0.16 (DCM/MeOH 9:1). ^1^H NMR (400 MHz, methanol-*d*_4_) δ: 8.09 (dd, *J* = 26.6, 7.8 Hz, 1H), 7.82 (d, *J* = 7.5 Hz, 3H), 7.68 (d, *J* = 3.8 Hz, 2H), 7.42 (t, *J* = 7.4 Hz, 2H), 7.33 (t, *J* = 7.5 Hz, 2H), 7.27–7.17 (m, 5H), 4.66 (d, *J* = 6.0 Hz, 1H), 4.45 (d, *J* = 7.2 Hz, 1H), 4.35 (s, 2H), 4.24 (s, 1H), 3.89 (d, *J* = 1.8 Hz, 2H), 3.76 (q, *J* = 16.7 Hz, 2H), 3.19 (dd, *J* = 14.0, 5.5 Hz, 1H), 2.98 (dd, *J* = 14.1, 8.5 Hz, 1H), 1.65 (d, *J* = 4.6 Hz, 3H), 1.04–0.75 (m, 6H). ^13^C NMR (101 MHz, methanol-*d*_4_) δ: 173.78, 172.96, 159.58, 145.71, 145.63, 143.02, 138.54, 130.75, 129.97, 129.24, 128.60, 128.25, 126.70, 121.36, 68.78, 67.31, 55.84, 53.56, 48.70, 45.42, 42.31, 42.20, 42.05, 38.82, 26.09, 24.50, 22.98, 17.82. MS (ESI^+^) *m*/*z* calculated for [C_34_H_38_N_4_O_7_]^+^, 615.3 [M + H]^+^; *m*/*z* obtained, 615.3.

#### Fmoc-VCit-OH (**5c**)

Synthesized by SPPS according to general procedure B. More specifically, 800 mg of 2-CTC resin was used and loaded with 890 mg of Fmoc-Cit-OH (2.24 mmol, 2 equiv); the calculated experimental loading was 1.2 mmol/g. A crude peptide was precipitated from cold diethyl ether and used in the next step without further purifications. A white solid was obtained (240 mg, 0.483 mmol, 66% yield). *R*_f_ = 0.14 (DCM/MeOH 9:1). ^1^H NMR (400 MHz, methanol-*d*_4_) δ: 7.80 (d, *J* = 7.5 Hz, 2H), 7.67 (t, *J* = 7.1 Hz, 2H), 7.35 (dt, *J* = 32.1, 7.4 Hz, 4H), 4.48–4.31 (m, 3H), 4.23 (s, 1H), 3.97 (s, 1H), 3.10 (s, 2H), 2.13–1.25 (m, 6H), 0.97 (dd, *J* = 11.5, 6.8 Hz, 6H) MS (ESI^+^) *m*/*z* calculated for [C_26_H_32_N_4_O_6_]^+^, 497.2 [M + H]^+^; *m*/*z* obtained, 497.4.

### Solution Phase Synthesis

#### Fmoc-VA-OH (**5d**)

To a solution of Fmoc-Val-OH (667 mg, 2 mmol, 1 equiv) dissolved in 100 mL of acetonitrile, TEA (0.31 mL, 2.2 mmol, 1.1 equiv) was added dropwise. The reaction mixture was cooled to 0 °C in an ice bath. Then, 4-NPC (440 mg, 2.2 mmol, 1.1 equiv) and DMAP (24 mg, 2.2 mmol, 1.1 equiv) were sequentially added and the reaction mixture was stirred for 1 h at 0 °C. When TLC (DCM/MeOH 10:1) showed complete consumption of the starting materials, the reaction was warmed to room temperature. A solution of l-alanine (713 mg, 8 mmol, 4 equiv) and TEA (1.12 mL, 8 mmol, 4 equiv) dissolved in 100 mL of water was added dropwise over 20 min. The reaction mixture was stirred for 2 h at room temperature (complete conversion of the intermediate mixed carbonate checked by TLC). Upon reaction completion, the organic solvent was removed under reduced pressure, and the aqueous phase was treated with an aqueous solution of HCl 2 mol/L giving a white suspension. The solid was recovered by filtration, washed with HCl 1 mol/L and water, dried under vacuum, and purified by flash chromatography (gradient elution from 100% DCM to DCM/MeOH 8:2) affording a white solid (412 mg, 1 mmol, 50% yield). *R*_f_ = 0.40 (DCM/MeOH 10:1). The ^1^H NMR spectrum (not reported) is in accordance with the literature.^34^ MS (ESI^+^) *m*/*z* calculated for [C_23_H_26_N_2_O_5_]^+^, 411.1 [M + H]^+^; *m*/*z* obtained, 411.1.

#### Fmoc-GPLG-*N*-4-(hydroxymethyl)­phenyl (**6a**)

Fmoc-GPLG-OH (**5a**) (560 mg, 0.992 mmol, 1 equiv) was dissolved in 800 μL of DCM and 600 μL of MeOH (2:1) under an argon atmosphere. Then, EEDQ (490 mg, 0.20 mmol, 2 equiv) and 4-aminobenzyl alcohol (240 mg, 0.20 mmol, 2 equiv) were added and the reaction mixture was stirred for 24 h at room temperature. The solvent was then removed under reduced pressure, the crude product was precipitated from cold diethyl ether and used in the next step without further purification. A yellow solid was obtained (540 mg, 0.806 mmol, 82% yield). *R*_f_: 0.55 (DCM/MeOH = 9:1). ^1^H NMR (400 MHz, acetone-*d*_6_) δ: 8.91 (s, 1H), 7.90 (s, 1H), 7.86 (d, *J* = 7.5 Hz, 2H), 7.79 (d, *J* = 8.2 Hz, 2H), 7.70 (d, *J* = 5.3 Hz, 2H), 7.41 (t, *J* = 7.4 Hz, 2H), 7.32 (t, *J* = 7.4 Hz, 2H), 7.27 (d, *J* = 8.3 Hz, 2H), 6.73 (s, 1H), 4.55 (s, 2H), 4.43 (dd, *J* = 8.1, 4.4 Hz, 1H), 4.32 (d, *J* = 7.1 Hz, 2H), 4.27–4.20 (m, 2H), 4.05 (s, 1H), 3.93 (d, *J* = 9.8 Hz, 4H), 3.70 (dd, *J* = 14.1, 7.0 Hz, 2H), 2.80 (d, *J* = 12.5 Hz, 2H), 2.32–2.21 (m, 1H), 2.14–2.08 (m, 1H), 1.76–1.65 (m, 3H), 0.92 (d, *J* = 5.6 Hz, 3H), 0.89 (d, *J* = 5.6 Hz, 3H). ^13^C NMR (101 MHz, acetone-*d*_6_) δ: 174.02, 173.94, 173.00, 172.90, 170.38, 168.53, 168.46, 157.78, 145.14, 144.93, 142.13, 142.10, 138.83, 138.74, 138.55, 128.57, 128.55, 127.98, 127.88, 127.79, 126.19, 126.13, 120.82, 120.09, 120.01, 67.52, 64.45, 64.33, 62.42, 62.39, 53.83, 53.73, 47.92, 47.58, 44.23, 44.13, 40.04, 39.99, 30.42, 30.22, 30.03, 29.94, 29.84, 29.65, 29.46, 29.26, 25.79, 25.71, 23.49, 21.65. MS (ESI^+^) *m*/*z* calculated [C_37_H_44_N_5_O_7_]^+^, 670.3 [M + H]^+^; *m*/*z* obtained, 671.4.

#### Fmoc-GFLG-*N*-4-(hydroxymethyl)­phenyl (**6b**)

Fmoc-GFLG-OH (**5b**) (249 mg, 0.405 mmol, 1 equiv) was dissolved in a mixture composed of 1.4 mL of dry MeOH and 5.5 mL of dry DCM under a nitrogen atmosphere. EEDQ (153 mg, 0.62 mmol, 1.5 equiv) and 4-aminobenzyl alcohol (60 mg, 0.49 mmol, 1.2 equiv) were then added and the reaction mixture was stirred overnight at room temperature. The solvents were then removed under reduced pressure, and the crude product was precipitated from cold diethyl ether, affording a white solid. The product was used in the next step without further purification (283 mg, 0.393 mmol, 96% yield). *R*_f_ = 0.53 (DCM/MeOH 9:1). ^1^H NMR (400 MHz, DMSO-*d*_6_) δ: 9.77 (s, 1H), 8.16 (q, *J* = 6.8 Hz, 2H), 8.03 (d, *J* = 8.0 Hz, 1H), 7.89 (d, *J* = 7.5 Hz, 2H), 7.70 (d, *J* = 7.5 Hz, 2H), 7.56 (d, *J* = 8.2 Hz, 2H), 7.51 (t, *J* = 6.0 Hz, 1H), 7.41 (t, *J* = 7.4 Hz, 2H), 7.32 (t, *J* = 7.4 Hz, 2H), 7.21 (s, 5H), 7.15 (q, *J* = 4.3 Hz, 1H), 4.56 (s, 1H), 4.43 (d, *J* = 5.6 Hz, 2H), 4.25 (s, 3H), 3.90–3.82 (m, 2H), 3.72–3.46 (m, 2H), 3.04 (dd, *J* = 13.9, 4.6 Hz, 1H), 2.79 (dd, *J* = 13.9, 9.1 Hz, 1H), 1.56 (s, 3H), 0.87 (dd, *J* = 18.0, 6.5 Hz, 6H). ^13^C NMR (101 MHz, DMSO-*d*_6_) δ: 172.21, 171.06, 168.97, 167.32, 156.43, 143.80, 140.68, 137.60, 137.39, 129.19, 127.98, 127.60, 126.46, 125.21, 120.07, 118.79, 65.75, 62.57, 53.69, 51.35, 46.58, 43.29, 42.63, 40.65, 37.39, 24.06, 22.96, 21.65. MS (ESI^+^) *m*/*z* calculated for [C_41_H_45_N_5_O_7_]^+^, 720.3 [M + H]^+^; *m*/*z* obtained, 720.5.

#### Fmoc-VCit-*N*-4-(hydroxymethyl)­phenyl (**6c**)

Fmoc-VCit-OH (**5c**) (240 mg, 0.483 mmol, 1 equiv) was dissolved in a mixture composed of 1.6 mL of dry MeOH and 6.4 mL of dry DCM under a nitrogen atmosphere. Then, EEDQ (178 mg, 0.72 mmol, 1.5 equiv) and 4-aminobenzyl alcohol (71 mg, 0.58 mmol, 1.2 equiv) were added and the reaction mixture was stirred overnight at room temperature. After 23 h, further EEDQ (84 mg, 0.34 mmol, 0.7 equiv) and 4-aminobenzyl-alcohol (30 mg, 0.24 mmol, 0.5 equiv) were added and the reaction mixture was stirred for 2 h. After 25 h overall the solvent was removed under reduced pressure, the crude product was precipitated from cold diethyl ether and used in the next step without further purification. A white solid was obtained (283 mg, 0.470 mmol, 96% yield). *R*_f_ = 0.71 (DCM/MeOH 9:1). ^1^H NMR (400 MHz, DMSO-*d*_6_) δ: 9.96 (s, 1H), 8.08 (d, *J* = 7.6 Hz, 1H), 7.89 (d, *J* = 7.6 Hz, 2H), 7.74 (t, *J* = 8.1 Hz, 2H), 7.54 (d, *J* = 8.2 Hz, 2H), 7.46–7.29 (m, 5H), 7.23 (d, *J* = 8.1 Hz, 2H), 5.95 (d, *J* = 5.9 Hz, 1H), 5.39 (s, 2H), 5.08 (t, *J* = 5.7 Hz, 1H), 4.43 (d, *J* = 5.4 Hz, 3H), 4.36–4.17 (m, 3H), 3.99–3.85 (m, 1H), 3.09–2.83 (m, 2H), 2.10–1.16 (m, 6H), 0.87 (dd, *J* = 10.8, 6.7 Hz, 6H). ^13^C NMR (101 MHz, DMSO-*d*_6_) δ: 171.19, 170.34, 158.82, 156.08, 143.88, 143.75, 140.68, 137.48, 137.42, 127.61, 127.04, 126,88, 125.33, 120.06, 118.84, 65.65, 62.36, 60.08, 53.03, 46.66, 30.41, 29.52, 26.74, 19.19, 18.22. MS (ESI^+^) *m*/*z* calculated for [C_33_H_39_N_5_O_6_]^+^, 602.3 [M + H]^+^; *m*/*z* obtained, 602.3.

#### Fmoc-VA-*N*-4-(hydroxymethyl)­phenyl (**6d**)

Fmoc-VA-OH (**5d**) (480 mg, 1.17 mmol, 1 equiv) and *p*-aminobenzyl alcohol (172 mg, 1.40 mmol, 1.2 equiv) were suspended in dry DCM (50 mL). Then EEDQ (435 mg, 1.76 mmol, 1.5 equiv) was added. Then, 20 mL of MeOH was added, obtaining a clear solution. The reaction mixture was stirred at room temperature overnight under a nitrogen atmosphere. The solvent was then removed under reduced pressure, and the crude product was suspended in cold diethyl ether and filtered, affording a pale yellow solid (397 mg, 0.770 mmol, 64% yield). *R*_f_ = 0.27 (*n*-hexane/EtOAc 4:6). The ^1^H NMR spectrum (not reported) is in accordance with the literature.^34^ MS (ESI^+^) *m*/*z* calculated for [C_30_H_33_N_3_O_5_]^+^, 516.2 [M + H]^+^; *m*/*z* obtained, 516.4.

#### Fmoc-GPLG-*N*-4-(methyl)­phenyl 4-Nitrophenyl Carbonate (**7a**)

Fmoc-GPLG-*N*-4-(hydroxymethyl)­phenyl (**6a**) (194 mg, 0.290 mmol, 1 equiv) was dissolved in 2.5 mL of dry THF under an argon atmosphere. Then, DIPEA (75 mg, 101 μL, 0.58 mmol, 2 equiv) was added and the reaction mixture was stirred at room temperature. After that, a solution of *bis* (4-nitrophenyl) carbonate (222 mg, 0.73 mmol, 2.5 equiv) dissolved in 2.9 mL of dry THF was added to the previous solution. Further additions of *bis* (4-nitrophenyl) carbonate (1 equiv after 1 h, 1 equiv after 3 h, 1 equiv after 4 h, and 0.5 equiv after 6 h) and DIPEA (1 equiv after 4 h and 0.7 equiv after 6 h) were performed. The reaction mixture was stirred at room temperature for 7 h overall and then THF was removed under reduced pressure. The crude product was purified by flash chromatography (eluent: DCM/acetone 7.5:2.5) affording a white solid (140 mg, 0.168 mmol, 58% yield). *R*_f_ = 0.36 (DCM/acetone 7.5:2.5). MS (ESI^+^) *m*/*z* calculated for [C_44_H_46_N_6_O_11_]^+^, 835.3 [M + H]^+^; *m*/*z* obtained, 835.3.

#### Fmoc-GFLG-*N*-4-(methyl)­phenyl 4-Nitrophenyl Carbonate (**7b**)

Fmoc-GFLG-*N*-4-(hydroxymethyl)­phenyl (**6b**) (100 mg, 0.139 mmol, 1 equiv) was dissolved in 2.6 mL of dry DMF under an Ar atmosphere. Then DIPEA (36 mg, 0.28 mmol, 50 μL, 2 equiv) and *bis* (4-nitrophenyl) carbonate (106 mg, 0.35 mmol, 2.5 equiv) were added, and the reaction was stirred at room temperature for 6 h. After 6 h, an additional amount of *bis* (4-nitrophenyl) carbonate (21 mg, 0.07 mmol, 0.5 equiv) was added, and the reaction was stirred for another hour. The solvent was then removed, affording the crude product as a white solid; it was used in the next step without further purification (97 mg, 0.110 mmol, 78% yield). *R*_f_ = 0.59 (DCM/Acetone 7:3). MS (ESI^+^) *m*/*z* calculated for [C_48_H_48_N_6_O_11_]^+^, 885.3 [M + H]^+^; *m*/*z* obtained, 885.2.

#### Fmoc-VCit-*N*-4-(methyl)­phenyl 4-Nitrophenyl Carbonate (**7c**)

Fmoc-VCit-*N*-4-(hydroxymethyl)­phenyl (**6c**) (60 mg, 0.100 mmol, 1 equiv) was initially suspended in 1.6 mL of dry THF under an argon atmosphere; 700 μL of dry DMF was then added to get a clear solution. DIPEA (26 mg, 35 μL, 0.2 mmol, 2 equiv) was added, followed by *bis* (4-nitrophenyl) carbonate (76 mg, 0.25 mmol, 2.5 equiv), and the reaction mixture was stirred at room temperature for 2 h. Further *bis* (4-nitrophenyl) carbonate (76 mg, 0.25 mmol, 2.5 equiv) was added to the reaction mixture, and after 6 h the solvent was removed under reduced pressure and the crude product was precipitated from DCM affording a white solid and used in the next step without further purification (63 mg, 0.0821 mmol, 83% yield). MS (ESI^+^) *m*/*z* calculated for [C_40_H_42_N_6_O_10_]^+^, 767.3 [M + H]^+^; *m*/*z* obtained, 767.3.

#### Fmoc-VA-*N*-4-(methyl)­phenyl 4-Nitrophenyl Carbonate (**7d**)

To a solution of Fmoc-VA-*N*-4-(hydroxymethyl)­phenyl (**6d**) (400 mg, 0.75 mmol, 1 equiv) dissolved in 20 mL of THF, 0.27 mL of pyridine (3.25 mmol, 2.5 equiv) was added dropwise. After the addition, the reaction mixture was cooled to 0 °C in an ice bath. Then, 4-NPC (681 mg, 3.38 mmol, 4.5 equiv) was added, and the reaction mixture was stirred at room temperature for 4 h. After the reaction completion, solvents were removed under reduced pressure and the crude product was purified by flash chromatography (gradient elution from 1:1 EtOAc/*n*-hexane to 100% EtOAc) affording a white solid (373 mg, 0.548 mmol, 71% yield). *R*_f_ = 0.45 (*n*-hexane/EtOAc 4:6).

#### Fmoc-GPLG-*N*-4-(methyloxycarbonyl-(*N*-Boc-*N*,*N*′-dimethylethylenediamine))­phenyl (**8a**)

Fmoc-GPLG-*p*NP (**7a**) (70 mg, 0.084 mmol, 1 equiv) was dissolved in 1.9 mL of THF under an argon atmosphere, and the solution was cooled to 0 °C. *N*-Boc*-N*,*N*′-dimethylethylenediamine (32 mg, 0.168 mg, 2 equiv) and pyridine (17 mg, 0.21 mmol, 2.5 equiv) were added, and the reaction mixture was stirred at room temperature for 2 h. The solvent was then removed under reduced pressure, and the crude product was dissolved in DCM and washed with a 1 mol/L solution of KHSO_4_ (2×), saturated solution of Na_2_CO_3_ (3×), and then with brine (2×). The organic phase was dried over anhydrous Na_2_SO_4_, filtered, and volatiles were removed under reduced pressure. The crude product was purified by flash chromatography (gradient elution from 97:3 to 96:4 DCM/MeOH) affording a white solid (51 mg, 0.058 mmol, 69% yield). *R*_f_ = 0.42 (DCM/MeOH 9:1). ^1^H NMR (400 MHz, CDCl_3_) δ: 7.66 (dd, *J* = 14.5, 7.8 Hz, 3H), 7.58 (s, 1H), 7.49 (t, *J* = 7.9 Hz, 2H), 7.37–7.06 (m, 6H), 5.94 (s, 1H), 4.97 (s, 2H), 4.35 (dd, *J* = 8.2, 4.6 Hz, 1H), 4.26 (dd, *J* = 10.3, 7.4 Hz, 1H), 4.19 (dd, *J* = 10.4, 6.7 Hz, 1H), 4.10 (t, *J* = 7.1 Hz, 1H), 4.04–3.85 (m, 2H), 3.72 (s, 2H), 3.54 (t, *J* = 8.0 Hz, 1H), 3.30 (s, 4H), 2.94–2.74 (m, 4H), 2.68 (s, 1H), 2.14 (s, 1H), 1.99 (s, 2H), 1.89 (s, 1H), 1.73 (s, 2H), 1.58 (s, 1H), 1.39 (d, *J* = 10.2 Hz, 9H), 1.30–1.09 (m, 2H), 0.83 (dd, *J* = 14.6, 6.7 Hz, 6H). ^13^C NMR (101 MHz, CDCl_3_) δ: 173.19, 169.96, 168.46, 157.55, 144.09, 141.91, 138.45, 132.72, 129.13, 128.18, 127.49, 125.46, 125.41, 120.39, 120.30, 68.06, 67.79, 61.80, 53.73, 47.52, 47.32, 44.24, 43.90, 39.35, 35.28, 30.07, 29.33, 28.84, 28.79, 25.59, 25.43, 24.88, 23.47, 21.72. MS (ESI^+^) *m*/*z* calculated for [C_47_H_61_N_7_O_10_]^+^, 884.5 [M + H]^+^; *m*/*z* obtained, 884.5.

#### Fmoc-GFLG-*N*-4-(methyloxycarbonyl-(*N*-Boc-*N*,*N*′-dimethylethylenediamine))­phenyl (**8b**)

Fmoc-GFLG-PAB-*p*NP (**7b**) (80 mg, 0.090 mmol, 1 equiv) was dissolved in 1 mL of dry THF under an argon atmosphere and the solution was cooled to 0 °C. Then, *N*-Boc-*N*,*N*′-dimethylethylenediamine (34 mg, 0.18 mmol, 2 equiv) and DIPEA (39 μL, 0.225 mmol, 2.5 equiv) were dissolved in the remaining 1 mL of THF, and the solution was added to the previous mixture slowly. The reaction mixture was stirred at room temperature for 2 h. The solvent was then removed under reduced pressure and the crude product was dissolved in DCM and washed with an 1 mol/L aqueous solution of KHSO_4_ (2×), saturated aqueous solution of Na_2_CO_3_ (3×), and with brine (2×). The organic phase was dried over anhydrous Na_2_SO_4_, filtered, and volatiles were removed under reduced pressure. The crude product was purified by flash chromatography (gradient elution from 97:3 to 95:5 DCM/MeOH) affording a white solid (64 mg, 0.069 mmol, 76% yield). *R*_f_ = 0.33 (DCM/MeOH 9:1). MS (ESI^+^) *m*/*z* calculated for [C_51_H_63_N_7_O_10_]^+^, 934.7 [M + H]^+^; *m*/*z* obtained, 934.8.

#### Fmoc-VCit-*N*-4-(methyloxycarbonyl-(*N*-Boc-*N*,*N*′-dimethylethylenediamine))­phenyl (**8c**)

Fmoc-Val-Cit-*p*NP (**7c**) (14 mg, 0.018 mmol, 1 equiv) was dissolved in 480 μL of THF under an argon atmosphere and the solution was cooled to 0 °C. Then, *N*-Boc*-N*,*N*′-dimethylethylenediamine (4 mg, 0.02 mmol, 1.1 equiv) was gradually added to the reaction mixture and it was stirred at room temperature for 1.5 h. The solvent was then removed under reduced pressure. The crude product was purified by flash chromatography (gradient elution from 97:3 to 94:6 DCM/MeOH) to afford a white solid (10 mg, 0.012 mmol, 67% yield). *R*_f_ = 0.42 (DCM/MeOH 94:6). MS (ESI^+^) *m*/*z* calculated for [C_43_H_57_N_7_O_9_]^+^, 816.4 [M + H]^+^; *m*/*z* obtained, 816.4.

#### Fmoc-VA-*N*-4-(methyloxycarbonyl-(*N*-Boc-*N*,*N*′-dimethylethylenediamine))­phenyl (**8d**)

Fmoc-VA-PAB-*p*NP (**7d**) (370 mg, 0.5 mmol, 1 equiv) was dissolved in 20 mL of dry THF under a N_2_ atmosphere. Then, the reaction mixture was cooled to 0 °C in an ice bath and *N*-Boc*-N*,*N*′-dimethylethylenediamine (188 mg, 1 mmol, 2 equiv) and DIPEA (0.22 mL, 1.25 mmol, 2.5 equiv) were sequentially added. The reaction mixture was stirred at room temperature for 48 h. The solvent was then removed under reduced pressure, and the crude product was diluted in EtOAc and washed with 1 mol/L KHSO_4_ (3×), saturated aqueous solution of NaHCO_3_ (3×), and with brine. The organic phase was dried with Na_2_SO_4_, filtered, and concentrated under reduced pressure to afford a pale-yellow solid product (379 mg, 0.519 mmol, 95% yield). *R*_f_ = 0.38 (EtOAc/*n*-hexane 7:3). HRMS (ESI^+^) *m*/*z* calculated for [C_40_H_51_N_5_O_8_]^+^, 730.3 [M + H]^+^; *m*/*z* obtained, 730.9.

#### Fmoc-GPLG-*N*-4-(methyloxycarbonyl-(*N*,*N*′-dimethylethylenediamine))­phenyl (**9a**)

Fmoc-GPLG-PABC-(Boc)­diamine (**8a**) (51 mg, 0.058 mmol, 1 equiv) was dissolved in 1.9 mL of dry DCM, and the solution was cooled to 0 °C. Then, 380 μL of TFA was added dropwise. The reaction mixture was stirred at room temperature for 1 h. Volatiles were then removed under reduced pressure, and TFA was azeotropically removed by additions of toluene and diethyl ether. The crude product was purified by preparative-HPLC (gradient: 90% (H_2_O + 10% ACN)/10% (ACN + 10% H_2_O + 0.1% TFA) to 10% (H_2_O + 10% ACN)/90% (ACN + 10% H_2_O + 0.1% TFA) in 10 min to 5% (H_2_O + 10% ACN)/95% (ACN + 10% H_2_O + 0.1% TFA) in 7 min to 90% (H_2_O + 10% ACN)/10% (ACN +10% H_2_O + 0.1% TFA) in 17 min). The pure fractions were concentrated under reduced pressure, and the remaining aqueous solution was freeze-dried affording a white solid (18 mg, 0.023 mmol, 40% yield). MS (ESI^+^) *m*/*z* calculated for [C_42_H_53_N_7_O_8_]^+^, 784.4 [M + H]^+^; *m*/*z* obtained, 784.7.

#### Fmoc-GFLG-*N*-4-(methyloxycarbonyl-(*N*,*N*′-dimethylethylenediamine))­phenyl (**9b**)

Fmoc-GFLG-PABC-(Boc)­diamine (**8b**) (64 mg, 0.068 mmol, 1eq ) was dissolved in 2.2 mL of dry DCM and the solution was cooled to 0 °C. Then, 500 μL of TFA was added dropwise. The reaction was stirred at room temperature for 1 h. Volatiles were removed under reduced pressure, and TFA was azeotropically removed through additions of toluene and diethyl ether, affording the product as a white solid (57 mg, 0.68 mmol, 88% yield). MS (ESI^+^) *m*/*z* calculated for [C_46_H_55_N_7_O_8_]^+^, 834.4 [M + H]^+^; *m*/*z* obtained, 834.3.

#### Fmoc-VCit-*N*-4-(methyloxycarbonyl-(*N*,*N*′-dimethylethylenediamine))­phenyl (**9c**)

Fmoc-VCit-PABC-(Boc)­diamine (**8c**) (24 mg, 0.029 mmol, 1 equiv) was dissolved in 800 μL of dry DCM and 70 μL of dry MeOH and the solution was cooled to 0 °C. Then, 800 μL of TFA was added dropwise, and the reaction mixture was stirred at room temperature for 5 h. Volatiles were then removed under reduced pressure and TFA was azeotropically removed by additions of toluene and diethyl ether, affording the crude product as a brown solid. The crude product was used in the next step without further purification (23 mg, 0.032 mmol, 96%). MS (ESI^+^) *m*/*z* calculated for [C_38_H_49_N_7_O_7_]^+^, 716.4 [M + H]^+^; *m*/*z* obtained, 716.4.

#### Fmoc-VA-*N*-4-(methyloxycarbonyl-(*N*,*N*′-dimethylethylenediamine))­phenyl (**9d**)

Fmoc-VA-PABC-(Boc)­diamine (**8d**) (45 mg, 0.062 mmol, 1 equiv) was dissolved in dry 2 mL of DCM, the solution was cooled to 0 °C and 400 μL of TFA was added dropwise. The reaction mixture was stirred at room temperature for 1 h. Volatiles were then removed under reduced pressure and TFA was azeotropically removed by additions of toluene and diethyl ether. The crude product was purified by preparative-HPLC (91 mg of the crude product) (gradient: 90% (H_2_O + 10% ACN)/10% (ACN + 10% H_2_O + 0.1% TFA) to 50% (H_2_O + 10% ACN)/50% (ACN + 10% H_2_O + 0.1% TFA) in 8 min to 40% (H_2_O + 10% ACN)/60% (ACN + 10% H_2_O + 0.1% TFA) 7 min to 25% (H_2_O + 10% ACN)/75% (ACN + 10% H_2_O + 0.1% TFA) in 3 min to 5% (H_2_O + 10% ACN)/95% (ACN + 10% H_2_O + 0.1% TFA) in 2 min to 90% (H_2_O + 10% ACN)/10% (ACN + 10% H_2_O + 0.1% TFA) in 8 min). The pure fractions were concentrated under reduced pressure, and the remaining aqueous solution was freeze-dried affording a white solid (32 mg, 0.043 mmol, 70% yield). MS (ESI^+^) *m*/*z* calculated for [C_35_H_44_N_5_O_6_]^+^, 631.3 [M + H]^+^; *m*/*z* obtained, 631.3. HRMS (ESI^+^) *m*/*z* calculated for [C_35_H_44_N_5_O_6_]^+^, 631.33644 [M + H]^+^; *m*/*z* obtained, 631.33608.

#### 2′-(*p*-Nitrophenyl)­PTX Carbonate (**10**)

PTX (50 mg, 0.059 mmol, 1 equiv) was dissolved in 1 mL of dry DCM under an argon atmosphere. Pyridine (23 mg, 24 μL, 0.295 mmol, 5 equiv) was then added, and the solution was cooled at 0 °C. A solution of 4-NPC (17.8 mg, 0.0885 mmol, 1.5 equiv) dissolved in 1 mL of dry DCM was added dropwise to the previous solution, and the reaction mixture was stirred for 1 h. Supplementary additions of 4-NPC (1 equiv) and pyridine (1 equiv) were added after 1, 2, and 3 h. Crude product was purified by flash chromatography (gradient elution from 7:3 to 5.5:4.5 *n*-hexane/AcOEt), affording a white solid (28 mg, 0.027 mmol, 47% yield). *R*_f_ = 0.29 (*n*-hexane/AcOEt 6:4). ^1^H NMR (400 MHz, CD_2_Cl_2_) δ: 8.32–8.22 (m, 2H), 8.19–8.13 (m, 2H), 7.81–7.72 (m, 2H), 7.63 (t, *J* = 7.4 Hz, 1H), 7.59–7.31 (m, 12H), 6.93 (d, *J* = 9.3 Hz, 1H), 6.37–6.22 (m, 2H), 6.08 (dd, *J* = 9.3, 2.8 Hz, 1H), 5.67 (d, *J* = 7.1 Hz, 1H), 5.55 (d, *J* = 2.8 Hz, 1H), 4.98 (d, *J* = 9.4 Hz, 1H), 4.42 (s, 1H), 4.31 (d, *J* = 8.4 Hz, 1H), 4.17 (d, *J* = 8.4 Hz, 1H), 3.81 (d, *J* = 7.1 Hz, 1H), 2.49 (s, 4H), 2.46–2.37 (m, 2H), 2.20 (s, 3H), 1.91 (d, *J* = 1.3 Hz, 3H), 1.87–1.76 (m, 2H), 1.64 (s, 3H), 1.24 (s, 3H), 1.13 (s, 3H). MS (ESI^+^) *m*/*z* calculated for [C_54_H_54_N_2_O_18_]^+^, 1019.3 [M + H]^+^; *m*/*z* obtained, 1020.2. HRMS (ESI^+^) *m*/*z* calculated for [C_54_H_54_N_2_O_18_]^+^, 1019.34444 [M + H]^+^; *m*/*z* obtained, 1019.3442.

#### Fmoc-GPLG-PABC-diamine-PTX (**1**)

Fmoc-GPLG-PABC-diamine (**9a**) (14 mg, 0.018 mmol, 1 equiv) was dissolved in 175 μL of dry DMF, previously degassed for 15 min through nitrogen flow, and cooled to 0 °C under an Ar atmosphere. Then, DIPEA (10 μL, 0.057 mmol, 3.1 equiv), 2′-*p*NP-PTX (**10**) (23 mg, 0.022 mmol, 1.2 equiv), and HOBt (0.3 mg, 0.002 mmol, 0.1 equiv) were added. The reaction mixture was stirred at room temperature for 1.5 h and DMF was removed under vacuum. The crude product was dissolved in 20 mL of ethyl acetate and washed two times with 5 mL of a 1 mol/L aqueous solution of KHSO_4_ and one time with 5 mL of a saturated aqueous solution of NH_4_Cl. The organic phase was dried over anhydrous Na_2_SO_4_, filtered, and the solvent was removed under reduced pressure. The crude product was purified by flash chromatography (eluent: DCM/MeOH 97:3), affording a white solid (18 mg, 0.011 mmol, 60%). *R*_f_ = 0.46 (DCM/MeOH 97:3). MS (ESI^+^) *m*/*z* calculated for [C_86_H_98_N_8_O_22_]^+^, 1663.7 [M + H]^+^; *m*/*z* obtained, 1663.7. HRMS (ESI^+^) *m*/*z* calculated for [C_54_H_54_N_2_O_18_]^+^, 1663.71306 [M + H]^+^; *m*/*z* obtained, 1663.7113.

#### Fmoc-GFLG-PABC-diamine-PTX (**2**)

Fmoc-GFLG-PABC-diamine (**9b**) (25 mg, 0.03 mmol, 1.5 equiv) was dissolved in 150 μL of dry DMF under an Ar atmosphere and the solution was cooled to 0 °C. Then, DIPEA (10.3 μL, 0.059 mmol, 3 equiv) was added dropwise followed by 2′-*p*NP-PTX (**10**) (20 mg, 0.02 mmol, 1 equiv) and HOBt (0.27 mg, 0.002 mmol, 0.1 equiv), and the reaction mixture was stirred at room temperature. Additionally, 2′-*p*NP-PTX (6 mg, 0,0072 mmol, 0.36 equiv) was added after 1 h. The reaction mixture was stirred for 3.5 h overall and DMF was removed under vacuum. The crude product was dissolved in 20 mL of ethyl acetate and washed two times with 5 mL of a 1 mol/L aqueous solution of KHSO_4_ and one time with 5 mL of a saturated aqueous solution of NH_4_Cl. The organic phase was dried over anhydrous Na_2_SO_4_, filtered, and the solvent was removed under reduced pressure. The crude product was purified by flash chromatography (eluent: DCM/MeOH 97:3), affording the pure product as a white solid (9.6 mg, 0.0056 mmol, 28% yield). *R*_f_ = 0.51 (DCM/MeOH 97:2). MS (ESI^+^) *m*/*z* calculated for [C_94_H_104_N_8_O_23_]^+^, 1713.7 [M + H]^+^; *m*/*z* obtained, 1714.4. HRMS (ESI^+^) *m*/*z* calculated for [C_54_H_54_N_2_O_18_]^+^, 1713.72871 [M + H]^+^; *m*/*z* obtained, 1713.7284.

#### Fmoc-VCit-PABC-diamine-PTX (**3**)

Fmoc-VCit-PABC-diamine (**9c**) (29 mg, 0.04 mmol, 1.5 equiv) was dissolved under an argon atmosphere in 188 μL of dry DMF, previously degassed for 15 min through nitrogen flow, and the solution was cooled to 0 °C. Then, DIPEA (13 μL, 0.075 mmol, 3 equiv) was added dropwise followed by 2′-*p*NP-PTX (**10**) (25 mg, 0.025 mmol, 1 equiv) and HOBt (0.4 mg, 0.003 mmol, 0.1 equiv). The reaction mixture was stirred at rt for 1.5 h, and DMF was removed under vacuum. The crude product was purified by flash chromatography (gradient elution from 97:3 to 95.5:4.5 DCM/MeOH). The product obtained from flash chromatography was further purified by preparative HPLC (gradient: 90% (H_2_O + 10% ACN)/10% (ACN + 10% H_2_O + 0.1% TFA) to 10% (H_2_O + 10% ACN)/90% (ACN + 10% H_2_O + 0.1% TFA) in 10 min to 5% (H_2_O + 10% ACN)/95% (ACN + 10% H_2_O + 0.1% TFA) in 7 min to 90% (H_2_O + 10% ACN)/10% (ACN + 10% H_2_O + 0.1% TFA) in 17 min). The pure fractions were concentrated under reduced pressure, and the remaining aqueous solution was freeze-dried, affording a white solid (8 mg, 0.005 mmol, 20% yield). *R*_f_ = 0.55 (DCM/MeOH 9:1). MS (ESI^+^) *m*/*z* calculated for [C_86_H_98_N_8_O_22_]^+^, 1595.7 [M + H]^+^; *m*/*z* obtained, 1596.7. HRMS (ESI^+^) *m*/*z* calculated for [C_54_H_54_N_2_O_18_]^+^, 1595.68684 [M + H]^+^; *m*/*z* obtained, 1595.6869.

#### Fmoc-VA-PABC-diamine-PTX (**4**)

Fmoc-Val-Ala-PABC-diamine (**9d**) (18 mg, 0.029 mmol, 1.5 equiv) was dissolved in 300 μL of dry DMF under an argon atmosphere and cooled to 0 °C. Then DIPEA (10 μL, 0.057 mmol, 3 equiv) and 2′-*p*NP-PTX (**10**) (20 mg, 0.019 mmol, 1 equiv) were sequentially added followed by HOBt (0.3 mg, 0.002 mmol, 0.1 equiv). After 1.5 h, DMF was removed under vacuum. The crude product was dissolved in 20 mL of ethyl acetate and washed two times with 5 mL of 1 mol/L solution of KHSO_4_ and with 5 mL of a saturated solution of NH_4_Cl. The organic phase was then dried over anhydrous Na_2_SO_4_, filtered, and the solvent was removed under reduced pressure. The crude product was purified by flash chromatography (eluent DCM/MeOH 97:3), affording a white solid (32 mg, 0.021 mmol, 73% yield). *R*_f_ = 0.40 (DCM/MeOH 97:3). MS (ESI^+^) *m*/*z* calculated for [C_86_H_98_N_8_O_22_]^+^, 1509.6 [M + H]^+^; *m*/*z* obtained, 1510.8. HRMS (ESI^+^) *m*/*z* calculated for [C_54_H_54_N_2_O_18_]^+^, 1509.63883 [M + H]^+^; *m*/*z* obtained, 1595.63834.

### Cathepsin B Cleavage Assay

The Cathepsin B cleavage assay was performed by incubating compounds **1–4** at 37 °C under gently shaking conditions in the presence of the active form of Cathepsin B. More specifically, 122.4 μL of an enzyme-activating solution (composed of 7.48 mg of l-cysteine dissolved in 2 mL of a buffer solution containing 20 mM NaOAc, 1 mM EDTA, pH 5.4 to give a final cysteine concentration of 30.88 mM) was mixed with 12.6 μL of enzyme stock solution, previously prepared by solubilization of bovine spleen Cathepsin B (SigmaAldrich C6286, ≥10 units/mg protein) in 1 mL of buffer solution (without cysteine) to give a concentration of enzyme stock solution of 12 units/mL. After the dilution, the final concentration of Cathepsin B was 1.12 u/mL. Then, the diluted enzyme solution was incubated at 37 °C for 15 min to activate Cathepsin B. After 15 min, 6 μL of 117 μM compound solution, previously prepared by diluting 5.85 μL of 1 mM stock solution of the compound in 44.15 μL of dry DMSO (giving a 117 μM solution), was added to the test tube and the cleavage assay solution was incubated at 37 °C under gentle shaking conditions. Sample aliquots (10 μL) were taken out at 15, 30 min, and 1, 2, 4, and 24 h and quenched with 10 μL of MeOH+1% formic acid. The solutions were then centrifuged for 10 min at 6000 rpm. Blank solutions were prepared for each compound by dilution of 6 μL of a 117 μM compound solution into 135 μL of an enzyme-activating solution. UHPLC-MS analyses were carried out using a Vanquish UHPLC System (Thermo Fisher Scientific) and Orbitrap Exploris 120 (Thermo Fisher Scientific). Thermo Scientific Xcalibur software was used to control the instruments and acquire the data. Freestyle 1.8 software was used for processing the data. UHPLC chromatographic separations were performed on a reversed-phase column Accucore C18 50 × 2.1 mm, 2.6 μm (Thermo Fisher Scientific). A gradient system was used with the mobile phase consisting of solvent A: H_2_O + 0.05% formic acid and solvent B: acetonitrile. The areas of the peak of compounds **1–4** in the extracted ion chromatogram were used to evaluate the kinetics of the cleavage process. The percentage of residual compound was calculated as the ratio between the areas of the peak of the full compound at the desired time point and in the blank analysis.

$$\%\;Residual\;compound=\frac{area\;compound\;at\;the\;desired\;time\;point}{area\;compound\;in\;the\;blank\;solution}\times 100$$

### pH Stability Assay

The pH stability assay was performed by incubating compounds **1–4** at 37 °C in buffers at pH 5.4 and 7.4 under gentle shaking conditions. More specifically, a solution of 20 mM AcONa and 1 mM EDTA in mQ water was used as a pH 5.4 buffer. A solution of PBS tablets in mQ water was used as a pH 7.4 buffer. A 1 mM stock solution of compounds **1–4** in dry DMSO was prepared and a 117 mM solution was then obtained by dilution of 5.85 μL of the stock solution in 44.15 μL of dry DMSO. Then, 6 μL of the 117 mM compound solution was diluted into 135 μL of the desired buffer, and the solution was incubated at 37 °C under gently shaking conditions. Samples aliquots (10 μL) were taken out at 15, 30 min and 1, 2, and 4 h and quenched with 10 μL of acetonitrile. The solutions were then injected in UHPLC-HRMS to monitor the stability of compounds **1–4** under different pH conditions. UHPLC-MS analyses were carried out as previously described for the Cathepsin B cleavage assay. The percentage of the residual compound was calculated as described above.

### Human and Rat Plasma Stability Assay

The plasma stability assays were performed by Selvita SA (Krakow, Poland) following the protocol described in the Supporting Information. For the use of human plasma, consent was obtained from the subject or from the next of kin.

## Results and Discussion

### Chemistry

Compounds **1**, **2**, **3**, and **4** were obtained through a straightforward synthetic pathway starting from the assembly of the respective Fmoc-protected peptide sequences GPLG, GFLG, VCit, and VA. Coupling reactions were performed activating the carboxyl function of Fmoc-protected amino acids using the uronium-type reagent COMU^41^ or the guanidinium-type reagent HATU.^42,43^ Cleavage of the Fmoc-protected peptide sequences from Wang resin was accomplished by treatment with the trifluoroacetic acid (TFA)/H_2_O/tri*iso*propyl silane (TIS) 95:2.5:2.5 mixture while to cleave Fmoc-VCit-OH **5c** from 2-CT resin, a 20% solution of hexafluoro*iso*propanol (HFIP) in dichloromethane was used (Scheme 1A). Conversely, the peptide sequence Fmoc-VA–OH **5d** was synthesized in solution in a two step-one pot procedure (Scheme 1B). All of the Fmoc-protected peptide intermediates were obtained in moderate to high yields (49–80%). The derivatization of **5a–d** at the C-*termini* is shown in Scheme 2. In more detail, the synthesized Fmoc-protected peptide sequences **5a–d** were condensed to *p*-aminobenzyl alcohol in good to excellent yields (64–96%) in the presence of *N*-ethoxycarbonyl-2-ethoxy-1,2-dihydroquinoline (EEDQ), a particularly useful reagent for the coupling of aromatic amines in amide bond formation.^44^ Finally, the complete installation of the self-immolative spacer PABC-*N*,*N*′-dimethylethylendiamine was accomplished in two steps. At first, the activation of the benzyl alcohol functionality in **6a-d** as *p*-nitrophenyl carbonate was performed using *bis*(*p*-nitrophenyl)­carbonate in the presence of pyridine, affording the reactive carbonates **7a–d** in 58%, 78%, 83% and 71% yields, respectively. Then, alkoxycarbonylation of commercially available Boc-protected-*N*,*N*′-dimethylethylendiamine was achieved through nucleophilic acyl substitution of the intermediate carbonates **7a–d**. In particular, carbonate **7a**,**b**,**d** were reacted with 2 equiv of Boc-monoprotected diamine and in the presence of DIPEA as base, affording compounds **8a**,**b**,**d** in very good yields. By contrast, under the same conditions, a competition between the carbamate formation and the Fmoc removal from the valine residue was observed in compound **7c** (data not shown). The reaction was thus tested on **7c** using only a slight excess of Boc-monoprotected diamine (1.1 equiv), finally obtaining the desired carbamate **8c** as the sole product in 67% yield. Removal of the Boc-protecting group from **8a–d** under acidic conditions yielded the deprotected compounds **9a–d** (in 40%–88%–96%, and 70% yield, respectively), which were reacted with PTX activated as *p*-nitrophenylcarbonate **10** at the 2′ position.

**Scheme 1:**
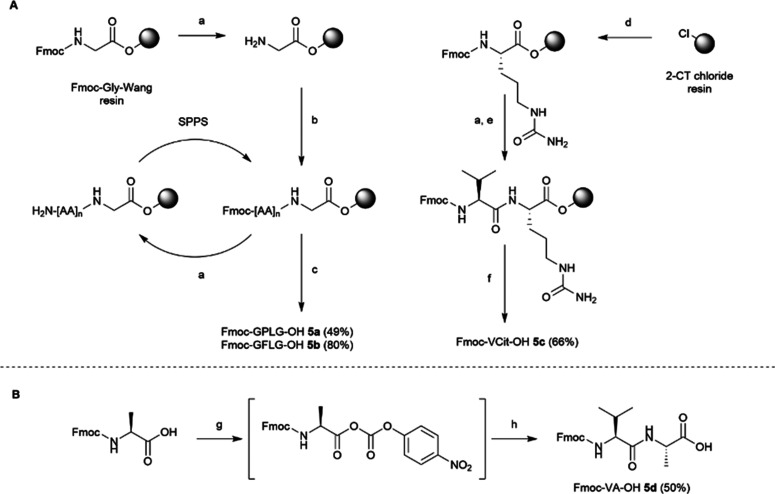
Synthesis of the Fmoc-Protected Sequences**5a–c** by SPPS (A) and Synthesis of Fmoc-VA–OH **5d** (B). (a) Piperidine in DMF (20% v/v); (b) Fmoc-AA–OH, COMU, DIPEA, DMF, r.t.; (c) TFA:H_2_O:TIS (95:2.5:2.5); (d) Fmoc-Cit–OH, DIPEA, DMF; (e) Fmoc-Val-OH, HATU, DIPEA, DMF/DCM, r.t.; (f) HFIP in DCM (20% v/v); (g) (*p*-Nitrophenyl)­carbonate, TEA, DMAP, MeCN; 1 h, 0 °C; h) (l)-Valine, TEA, MeCN/H_2_O, 1 h, r.t

**Scheme 2:**
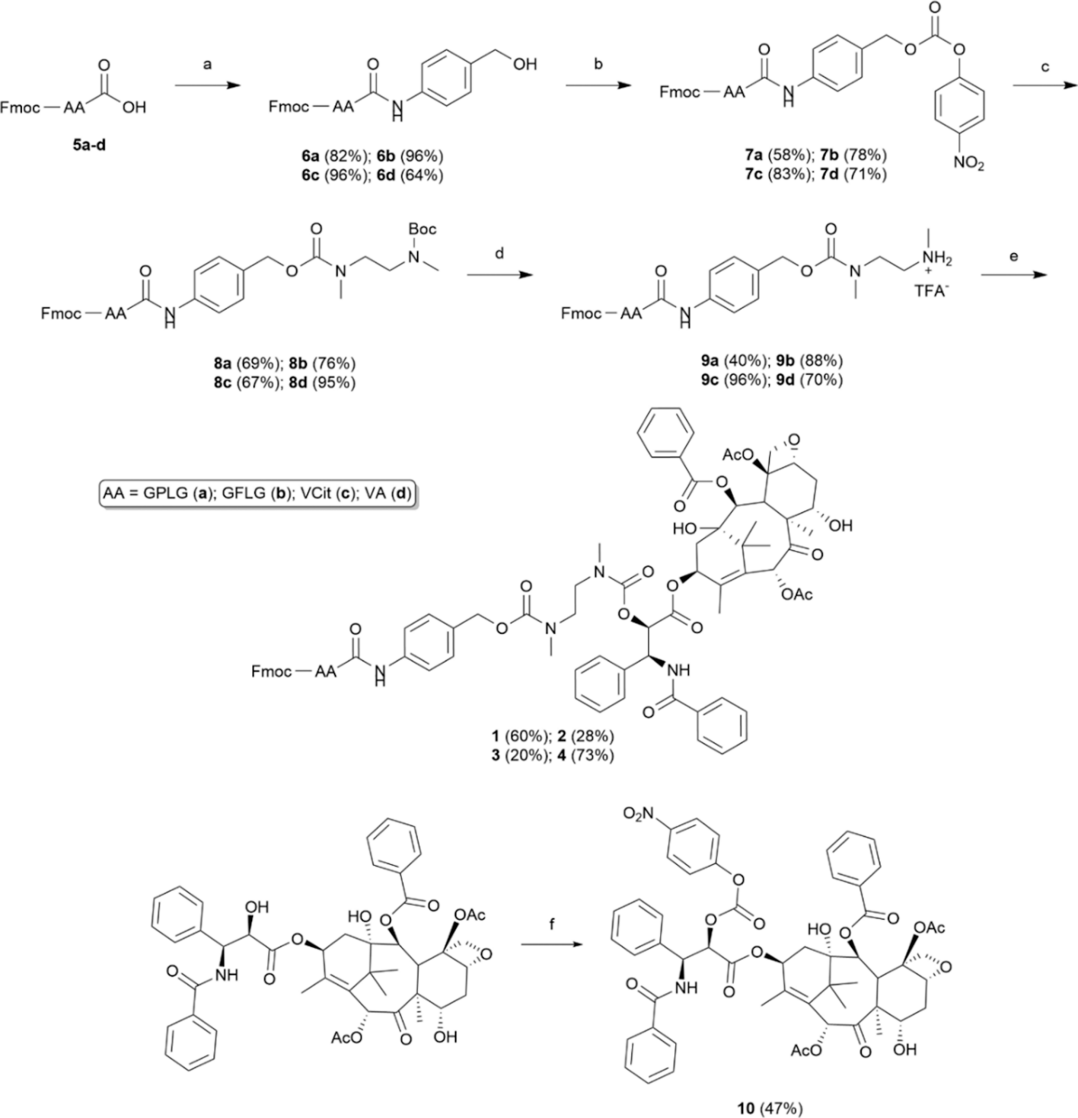
Synthetic Pathway for Preparing Final Compounds **1**, **2**, **3,** and **4** from Fmoc-Protected Peptides **5a–d**. (a) PABOH, EEDQ, DCM/MeOH, r.t.; (b) Bis­(*p*-Nitrophenyl)­carbonate, Pyridine, THF/DMF, 0 °C to r.t.; (c) *N*-Boc-*N*,*N*′-Dimethylethylenediamine, DIPEA, THF, 0 °C to r.t.; (d) TFA (20% v/v); DCM, 0 °C to r.t.; (e) **10**, DIPEA, HOBt, DMF, 0 °C to r.t.; f) *p*-Nitrophenylchloroformate, Pyridine, DCM, 0 °C to r.t

The latter was prepared from PTX in the presence of *p*-nitrophenylchloroformate and pyridine at 0 °C to ensure regioselectivity.^45^ The final nucleophilic acylation of the obtained PTX derivative **10** with the secondary amine of compounds **9a–d** was performed in the presence of DIPEA and of a substoichiometric amount of 1-hydroxybenzotriazole (HOBt), affording the desired purified compounds **1–4** in 60%, 28%, 20% and 73% yields with HPLC purity above 95%. Lower yields were obtained for compounds **2** and **3** due to a difficult final purification to obtain the high purity required for the enzymatic tests.

#### The Cathepsin B Enzyme Recognizes the GPLG-PABC Linker

Based on the similarity of the Cathepsin B recognized sequence GFLG to GPLG, we inferred that the latter could also be recognized by the same lysosomal enzyme. Therefore, a Cathepsin B cleavage assay for compound **1** was planned, and the experiment was designed to focus on a short incubation time (up to 4 h) to better mimic the fast transient and dynamic environment of drug clearance and activation generally occurring in *in vivo* conditions. An Orbitrap mass analyzer was chosen to analyze the final incubated solution due to its higher sensitivity compared to traditional HPLC methods, thereby allowing precise evaluation of the drug release kinetics even at low compound concentrations. At first, to validate the designed Orbitrap-protocol over the HPLC protocols generally reported in the literature, we initially performed the cleavage assay on compound **4** containing the VA sequence, for which the recognition by the Cathepsin B enzyme is well documented.^13,14,17–19^ For instance, compound **4** was incubated at 37 °C with a sodium acetate buffer solution (final concentration of 4: 5 μM) containing the active form of the Cathepsin B enzyme, and diluted aliquots at 0–30–60–120 and 240 min were collected and analyzed by a UHPLC-Orbitrap instrument and, in parallel, by analytical HPLC. As shown in Figure S26 of the Supporting Information, the degradation of the VA-based compound **4** can be appreciated from both analyses; however, an expected remarkable difference between the two methods was noted regarding the final concentration of the compound **4** at the end of the experiment. Indeed, according to the HPLC chromatogram, the residual compound **4** was almost fully degraded (about 7% of the initial concentration) after 4 h of incubation with the enzyme, whereas the Orbitrap instrument detected 47.4% of residual intact VA-based molecule, which was calculated as the ratio between the peak area of intact compound **4** at a given time point and its peak area in the control analysis (for details see the Supporting Information, Figure S26, Table S1). Thus, our direct comparison between the two detectors indicates that the high sensitivity of the Orbitrap instrument, in contrast to the less sensitive HPLC analysis, enables the detection of residual intact compound, an effect that could be attributed to the documented enzyme instability over time.^31,46–49^

After confirming the validity of our Orbitrap protocol, compound **1** was incubated with the Cathepsin B enzyme using the procedure described above, and aliquots at 0–2–15–30–60–120 and 240 min were collected and subsequently analyzed. As shown in Figure 2, the HRMS analysis clearly demonstrated that the concentration of compound **1** in the incubated solution decreased over time in the presence of the Cathepsin B enzyme, whereas no significant degradation was observed in the control solution (Figure 2). The HRMS spectra were then analyzed to identify the fragments resulting from enzymatic hydrolysis. As shown in Figure 3, the proteolytic activity of Cathepsin B should at first hydrolyze the amide bond between the *p*-aminobenzyl carbamate moiety and the C-terminal amino acid of the GPLG sequence (fragment I), with a subsequent 1,6-rearrangement mechanism with subsequent elimination of the PABC portion, giving rise to fragment II.

**Figure 2:**
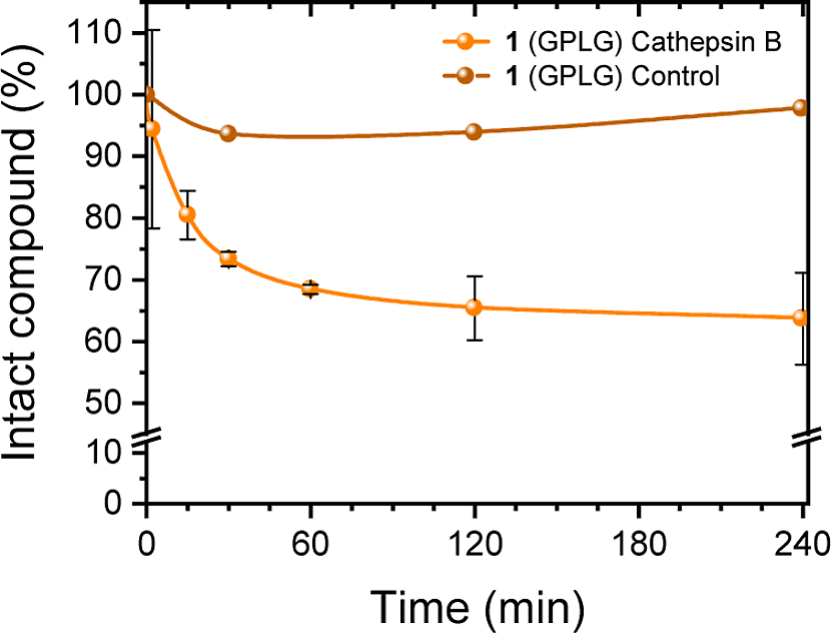
*In vitro* Cathepsin B enzymatic assay of compound **1** compared to its stability in the control solution without the enzyme. The analysis was repeated in triplicate for the cleavage assay and in duplicate for the stability assay. Full data are available in the Supporting Information, Tables S2 and S6.

**Figure 3:**
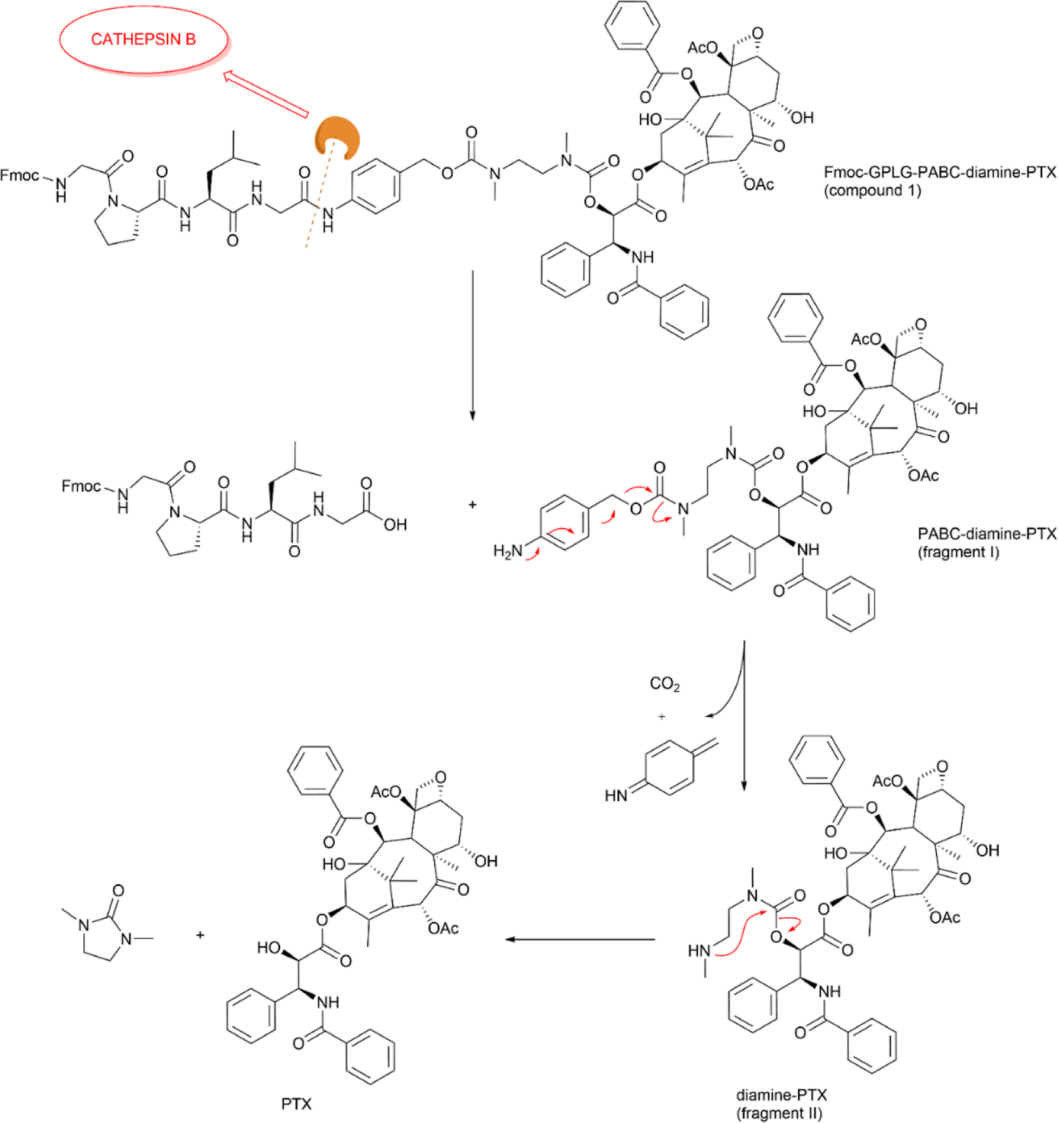
Mechanism of the release of PTX upon GPLG linker cleavage by Cathepsin B.

Finally, the intramolecular cyclization of the *N*,*N*′-dimethylethylenediamine should lead to the release of the PTX without any further derivatization (Figure 3). Interestingly, the PABC-diamine-PTX fragment (fragment **I**, Figure 3) and the diamine-PTX fragment (fragment II, Figure 3) were already observed at low intensity after 2 min of incubation by extracting the respective *m*/*z* from the TIC spectra, with fragment **II** detected with increasing abundance over the 4 h of the assay (Figure S33). A similar result was not obtained in the control sample incubated without the enzyme, thus clearly demonstrating that the GPLG sequence is effectively recognized and cleaved by Cathepsin B. On the other hand, at 4 h it was still not possible to observe the peak related to the free Paclitaxel (Figure S33). The absence of the free drug in the incubated solution at this time could be attributed to the reported slow kinetics of the amine cyclization, which represents the rate-limiting step in the drug release process, and that can be further slowed down by amine protonation under acidic pH.^50^ However, to experimentally demonstrate the occurrence of the cyclization of ethylenediamine moiety and the resulting release of the drug, a further analysis was performed after 24 h of incubation, which effectively confirmed the presence of the peak related to the free PTX (Figure S33). Finally, it appears that, also in this case, the initial compound **1** is not completely degraded, as can be inferred from the curve in Figure 2.

#### Comparison of the Drug-Release Kinetics of Compounds **1–4**

To evaluate the efficiency of the new GPLG sequence in comparison to the established Cathepsin B substrates GFLG, VCit, and VA, enzymatic assays were conducted for compounds **2**, **3**, and **4**, followed by analysis of respective aliquots at 0–2–15–30–60–120 and 240 min using HRMS. In all these cases, a reduction of the initial amounts of compounds **2**–**4** over time was observed, accompanied by the detection of the expected fragmentation patterns (fragment **I** and fragment **II** in Figure 3, and related HRMS spectra in Figures S34–S36), thus confirming the proteolytic activity of Cathepsin B. Remarkably, the cleavage kinetics of the GPLG-based compound **1** was the fastest during the initial time points (2 min to 1 h) and then became comparable to those of the VA-based compound **4**, with both reaching approximately 64% of residual starting compound after 4 h of incubation (Figure 4). In comparison to the GPLG- and VA-containing compounds, compound **3**, featuring the VCit linker, widely used for Cathepsin B-sensitive ADCs,^51^ showed a slower release process, with 76% of the residual compound after 4 h of incubation with the enzyme. Finally, the slowest cleavage kinetics among this series of derivatives was observed with the GFLG-based compound **2**, with approximately 80% of the intact starting material remaining after 4 h of incubation with the enzyme. Since in the literature the GFLG sequence is found mainly in SMDCs and PDCs with the payload conjugated either via the N-terminal or the C-terminal glycine residue of the tetrapeptide,^46,52,53^ we supposed that Cathepsin B can cleave the sequence at both Gly residues and that this fact could negatively affect the final kinetics of the drug release. The peak corresponding to compound **2** lacking the N-terminal Gly residue (fragment **IA**, Figure 5; MS (ESI^+^) *m*/*z* calculated for [C_77_H_91_N_7_O_20_]^+^: 1434.6392 [M + H]^+^; *m*/*z* observed: 1434.6395) was identified by the HRMS analysis extract after only 2 min of incubation with the enzyme (Figure 5). Analogously, the peak related to the PABC-diamine-PTX fragment (fragment **I**, Figure 3) was detected, suggesting that Cathepsin B cleaves both the peptide bond between the N-terminal Gly and Phe residues as well as the bond between the C-terminal Gly and the PABC moiety. A similar analysis was performed with the GPLG sequence; however, in this case no cleavage between Gly and Pro residues was observed throughout the experiment time.

**Figure 4:**
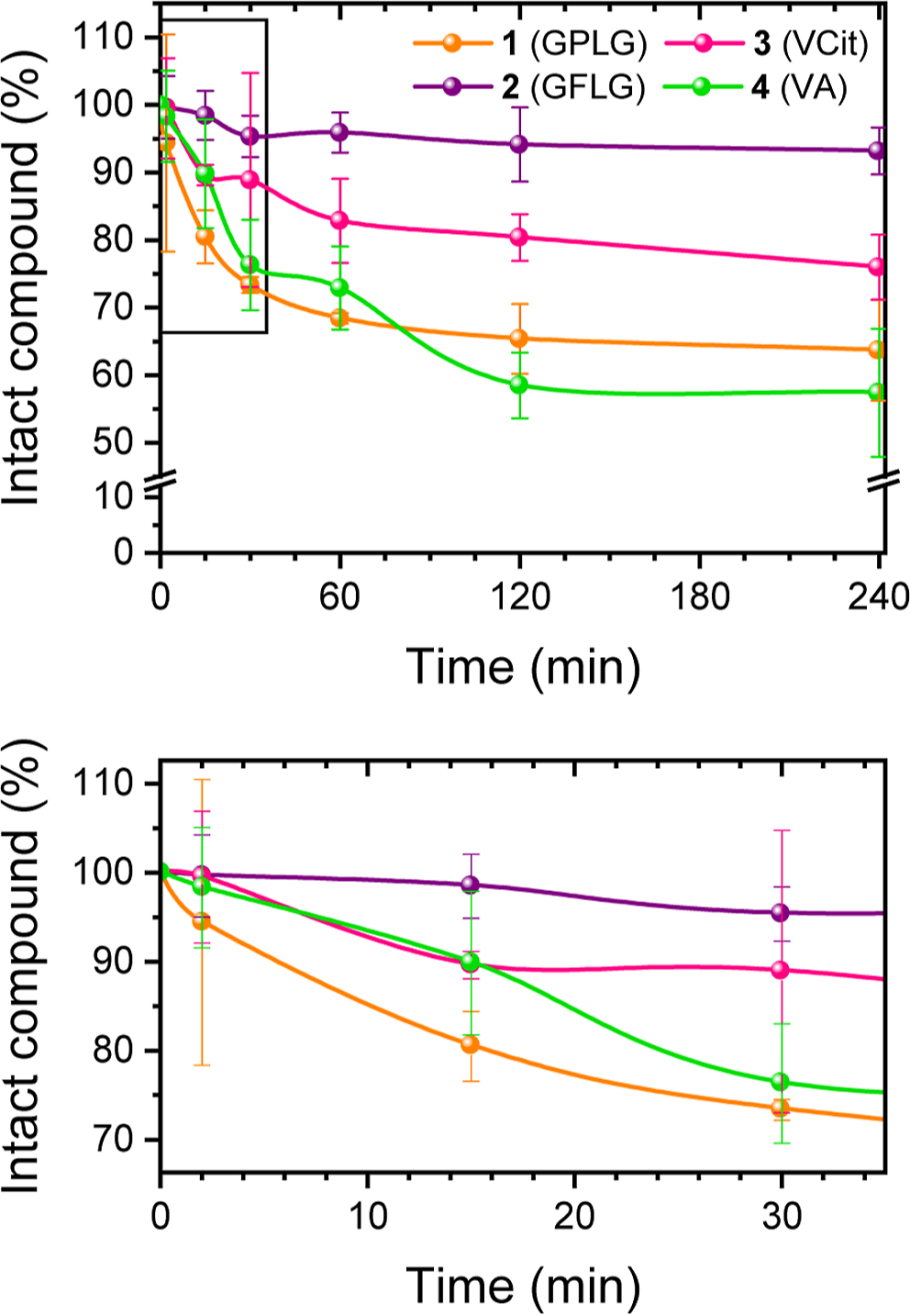
*In vitro* Cathepsin B enzymatic assay of compounds **1–4** in the time range 0–4 h (upper panel) and 0–35 min (the lower panel, corresponding to selected area in the upper panel). Final concentration of compounds **1–4** in the incubated solutions (pH = 5.4): 5 μM. The analysis was repeated in triplicate. Full data are available in the Supporting Information, Tables S2–S5.

**Figure 5:**
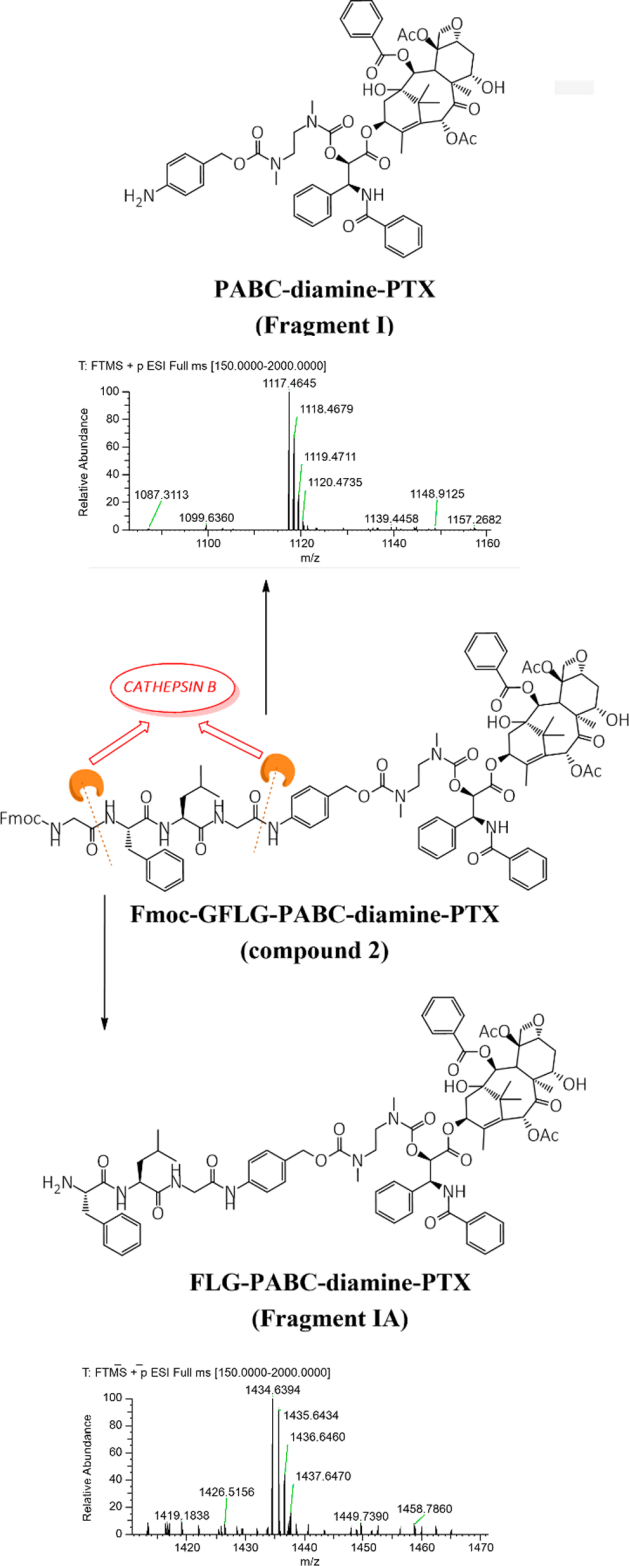
Two different cleavage mechanisms of the GFLG linker by Cathepsin B.

#### Comparison of the Stability Profile of Compounds **1–4**

The stability of compounds **1–4** and of the free drug PTX was evaluated *in vitro* by UHPLC-Orbitrap analysis incubating the samples at 37 °C in buffer solutions at pH 5.4, to simulate the pH inside the cancer cells, and at physiological pH 7.4. The same buffer of the Cathepsin B cleavage assay was used for the test at pH 5.4. The percentage of residual compounds **1–4** was measured over time by calculating the ratio between the areas of the peak of the full compound at the desired time point and at time zero, and the results were compared with the percentage of residual compounds **1–4** calculated during the Cathepsin B cleavage assay (Tables S6).

All tested compounds remained fully stable under physiological conditions (pH 7.4) for up to 4 h (Figure 6). However, distinct stability profiles emerged at pH 5.4. Among the tested compounds, the GPLG-based compound **1** exhibited the highest stability, with 97.8% of the starting material remaining after 4 h of incubation (Figure 6).

**Figure 6:**
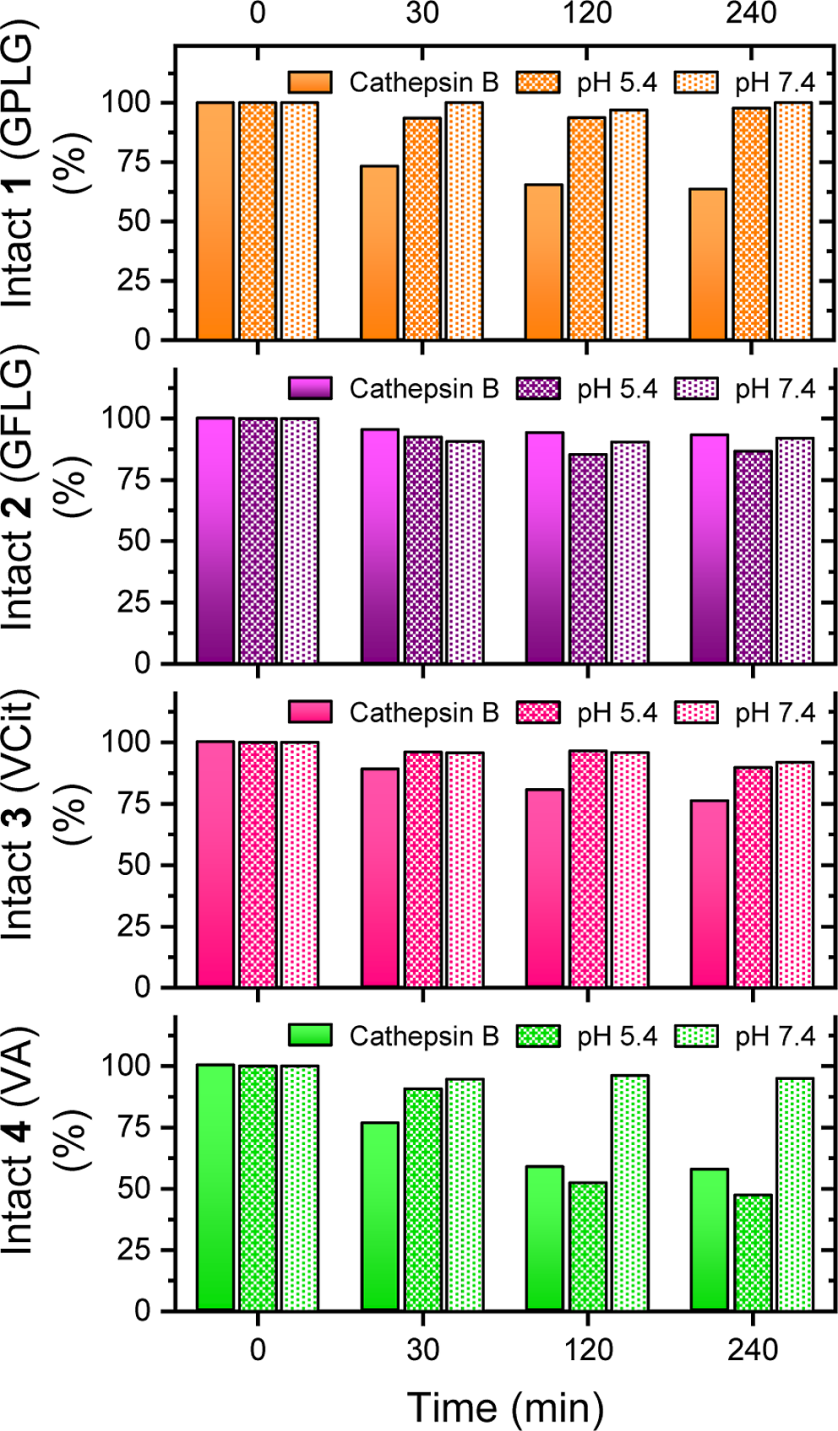
*In vitro* stability assay of compounds **1–4** at pH 5.4 and pH 7.4 compared to the Cathepsin B enzymatic assay results. The tests were performed by incubating the compound in the same aqueous buffer, and the analysis was repeated in duplicate. Full data are available in the Supporting Information, Table S6.

Similarly, VCit-based compound **3** demonstrated notable stability, retaining 90.9% of the starting material over the same period. Interestingly, the VA-based compound **2** and the GFLG-based compound **4** followed a stability trend comparable to their behavior in the enzymatic cleavage assay, with 86.76 and 47.4% of the starting material remaining, respectively, after 4 h (Figure 6). This suggests that the observed digestion of compounds **2** and **4** in the enzymatic assay may be partially due to degradation of the conjugates rather than specific cleavage by Cathepsin B. However, it is important to highlight that the stability profiles observed for compounds **1–4** may be specific to these constructs, and different trends could emerge when varying the payload attached to the peptide sequences.^11,54^

These findings, along with the faster cleavage of the GPLG peptide sequence than the VA-sequence, align with the previously observed higher antiproliferative potency of the GPLG-based MMAE conjugate compared to its VA-based counterpart.^31,32^

Indeed, the higher stability of the GPLG linker in a weak acidic environment is probably a key factor in this difference, as it allows the whole conjugate to bound to the integrin receptor and to remain intact long enough to facilitate drug internalization and exert its cytotoxic effects. Finally, plasma stability assays were conducted to evaluate the stability profiles of compounds **1–4** in whole plasma, accounting for both enzymatic and nonenzymatic degradation, as well as in the liver S9 fraction to assess metabolic degradation mediated by phase I and phase II liver enzymes (Figure 7).^55^ As shown in Figure 7, the GPLG-based compound **1** demonstrated great stability in both human and rat plasma samples, outperforming the other compounds, particularly in the S9 fraction assays.

**Figure 7:**
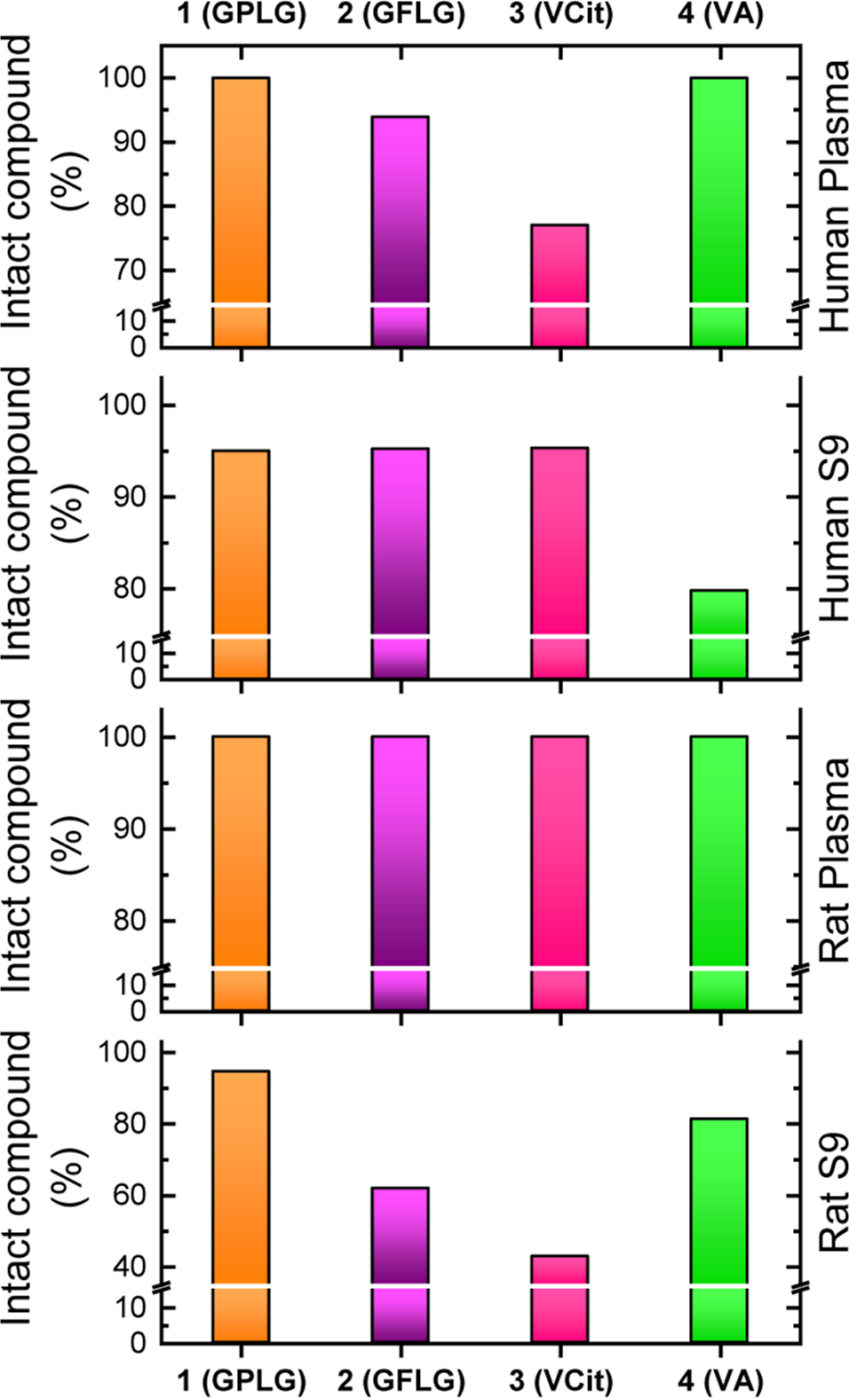
*In vitro* stability assay of compounds **1–4** in rat and human plasma and liver S9 fraction. The analysis were repeated in duplicate. The *y*-axes are broken to highlight the differences between the percentages. Full data are available in the Supporting Information, Tables S7–S10, and Figure S41.

It remained completely stable in full plasma samples (100% of residual compound after 4 h) and exhibited remarkable stability in the S9 fraction assays, with more than 94% of the compound remaining intact after 1.5 h of incubation. In comparison, the VA-based compound **4** displayed similar stability to compound **1** in full human and rat plasma but showed a significantly lower stability profile in the S9 fraction analysis, with residual compound levels of 79.75% in human S9 and 81.17% in rat S9 after 1.5 h. Finally, the VCit-based compound **3** exhibited the lowest stability in rat S9 fraction assays, retaining only 42.65% of the starting material after 1.5 h, and this result is in alignment with its known susceptibility to extracellular carboxylesterases, particularly in murine plasma.^51^

## Conclusions

Despite the central role of cleavable linkers in the design of tumor-targeting drug conjugates, systematic *in vitro* comparisons of their enzymatic cleavage efficiency, mass spectrometry fragmentation behavior, and plasma stability remain scarce in the literature. This gap highlights the need for comparative studies that can inform the rational selection of linker structures with optimal stability and release profiles. We previously reported that the replacement of the VA linker with the Gly-Pro-Leu-Gly (GPLG) sequence in a SMDC containing the potent antimitotic agent MMAE led to a significant enhancement of the final potency of the conjugate on different cancer cell lines.^26^ Herein, we explored in detail the Cathepsin B enzymatic susceptibility, cleavage kinetics, and metabolic stability of the GPLG tetrapeptide, which had not been addressed yet. Furthermore, we performed an *in vitro* comparative analysis with three well-known Cathepsin B-recognized sequences commonly used as cleavable linkers to release the conjugate’s payload at the site of interest. For instance, we synthesized compounds **1–4** bearing the GPLG, GFLG, VCit, and VA linker respectively, connected to the cytotoxic agent PTX as the model payload through the self-immolative spacer PABC-*N*,*N*′-dimethylethylendiamine. We then tested their ability to release the free payload upon Cathepsin B proteolytic activity by the UHPLC-Orbitrap mass spectrometer, ensuring high sensitivity compared to traditional HPLC methods. Our results demonstrate that GPLG is efficiently recognized and cleaved by Cathepsin B, exhibiting the fastest cleavage kinetics within the first 30 min of the assay. Remarkably, the GPLG-based compound **1** also showed higher chemical stability at pH 5.4 compared to compounds **2**–**4** as well as excellent stability in both human and rat plasma samples. We believe that our findings could be relevant for the rational design of future drug conjugates. With this aim, further studies are currently underway in our laboratories to validate the general efficacy of GPLG-based full conjugates, considering their short cleavage time and higher stability compared to the most used VA and VCit linkers, as well as to assess the influence of different payloads and targeting ligands on drug release, pharmacokinetics, and therapeutic efficacy *in vivo*.

## Supplementary Material



## Notes

General procedure for solid phase synthesis, NMR spectra, HPLC purity analysis, HRMS/LC–MS spectra, Cathepsin B cleavage assay protocol and HRMS analysis, pH stability assay protocol and HRMS analysis, and plasma stability assay (PDF)
