# Supplementary material for: Comparative Enzymatic and Stability Assays Reveal GPLG as an Effective Cathepsin B Cleavable Linker for Tumor-Targeting Drug Conjugates
Source: ACS Omega. 2025 Sep 4;10(36):41783–98. doi: 10.1021/acsomega.5c05758 (PMC12444563; doi:10.1021/acsomega.5c05758)
Supplement: Supplementary file 1 [file ao5c05758_si_001.pdf]

Supplementary Information

Comparative enzymatic and stability assays reveal GPLG as an effective Cathepsin B cleavable linker for tumor targeting drug conjugates

Giulia Cazzaniga‡[1], Marco Zambra‡[1], Samuele Bongiolo[1], Helena Prpic[1], Elettra Fasola[1], Federico Arrigoni[1], Umberto Piarulli*[,1] and Silvia Gazzola*[,1]

[1]Department of Science and High Technology, University of Insubria, via Valleggio 9, 22100, Como, Italy

1. General Information	S1
2. General procedure for solid phase peptide synthesis	S1
3. NMR spectra	S4
4. HPLC purity analysis	S11
5. HRMS / LC-MS spectra	S13
6. Validation of the Cathepsin B cleavage assay protocol – HPLC vs UHPLC-HRMS analysis	S17
7. Cathepsin B cleavage assay – UHPLC-HRMS analysis	S22
8. pH stability assay – UHPLC-HRMS analysis	S31
9. Plasma stability assay	S32

1. General Information

All commercially available reagents were purchased from Sigma-Aldrich, Fluorochem, Tokyo Chemical Industries, Alfa Aesar, Carlo Erba, VWR Chemicals and BLD Pharmatech and used as received without further purifications. All solution-phase reactions requiring anhydrous conditions were carried out under nitrogen or argon atmosphere with magnetic stirring. Anhydrous solvents were withdrawn from the container by syringe, under a slight positive pressure of nitrogen or argon. Reactions were checked by analytical thin-layer chromatography (TLC) using silica gel pre-coated ALUGRAM Xtra SIL G/UV254 plates (0.60 mm thickness) purchased from Macherey-Nagel. TLCs were examined by visualization under UV light (λ=254 nm) and/or staining with ninhydrin or ceric ammonium-molybdate. Purifications by flash-chromatography were performed using 60 Å, 230-400 mesh, 40-63 μm silica gel. Purifications by preparative HPLC were carried using a SHIMADZU LC-20AP prominence apparatus equipped with a FRC-10A fraction collector, SPD-M20A diode-array detector, CBM-20A system controller and a Sepachrom Robusta 100 Å C18 5 μm 250x21.2 mm column (flow 15 mL/min). All HPLC solvents were degassed for 90 minutes under ultrasonic treatment. Pure freeze-dried compounds were obtained from frozen aqueous solutions using a Telstar Lyo Quest -55 lyophiliser. Purities of synthetized compounds were analyzed by analytical HPLC SHIMADZU LC-20AP equipped with diode array UV detector and by Waters 600 HPLC System coupled with MS Waters Micromass ZQ, ESI source (flow 1 mL/min); both the instruments were equipped with Phenomenex LC column 150x4.6 mm Synergi 4 μm Fusion RP 80 Å. All employed HPLC-MS solvents were degassed for 20 minutes under a 100 mL/min helium flow. Solution 1H and 13C NMR spectra were recorded using a Bruker Avance 400 spectrometer operating respectively at 400.16 MHz and at 100.63 MHz. 1H and 13C chemical shifts are reported in ppm (δ) relative to TMS (internal standard). Coupling constants are reported in Hz and spin multiplicity is described as follow: s = singlet, d = doublet, t = triplet, dd = doublet of doublets, m = multiplet. High-resolution mass spectra (HRMS) were obtained with Thermo Fisher Scientific Orbitrap Exploris 120 equipped with Vanquish UHPLC System (Thermo Fisher Scientific) and reversed-phase column AccucoreTM C18 50x2.1 mm, 2.6 μm (Thermo Fisher Scientific).

2. General procedure for solid phase peptide synthesis

Fmoc-GPLG-OH (**5a**) and Fmoc-GFLG-OH (**5b**) and were synthetized manually on commercially available Fmoc-Gly-Wang resin (loading: 0.4 – 0.8 mmol/g) using the Fmoc protocol according to general procedure A. Fmoc-VCit-OH (**5c**) was synthetized on commercially available 2-CTC resin using the Fmoc protocol according to general procedure B.

General procedure A:

– Resin swelling: Fmoc-Gly-Wang resin ($L_{s(exp.)}$ 0.6 mmol/g) was swollen in DMF (enough volume to wet the resin completely) at room temperature for 30 minutes, then the solvent was discarded.

- Resin washing: washings were performed by alternating DMF (approximately 7 mL) and IPA (approximately 7 mL) stirring the syringe for 1 minute each time; the liquid was then discarded. The washing procedure was repeated 3 times (or until the discarded liquid is transparent). A last washing in DMF was performed. In case a HPLC-MS check is required after washing, an additional treatment with DCM is required to remove all traces of DMF.
- Deprotection: the resin was stirred for 3 minutes with a solution of 20% piperidine in DMF. The liquid was then discarded and the process was repeated 3 times in total.
- Resin washing.
- Fmoc-AA activation and coupling: the Fmoc-amino acid to be coupled (3 eq.) was solubilized in 5 mL of DMF, DIPEA (8.5 eq.) was added and the solution was stirred for 3 minutes. COMU (3 eq.) was then added and the reaction mixture was stirred until a bright red solution was obtained. This mixture was added to the resin and stirred for 1 h at room temperature. The coupling solution was then discarded and the resin was washed as described before. The completion of the coupling was verified by HPLC-MS. The reaction was repeated in case a partial coupling was detected.
- The procedures for deprotection and Fmoc-AA activation and coupling were repeated to complete the peptide sequence.
- Resin cleavage: after the completion of peptide sequence, the resin was washed two times (1 minute each) with DCM to remove traces of DMF. Then, 3 mL of a solution composed by TFA/TIS/water 95:2.5:2.5 was added and the resin was swollen for 1 h at room temperature. The solution was collected, and the procedure was repeated by swelling the resin for 30 minutes with fresh cleavage solution. The obtained solution was concentrated under reduced pressure (no heating above 35 °C) and the crude peptide was precipitated from cold diethyl ether (10 mL of diethyl ether per mL of concentrated cleavage solution). The suspension was centrifugated (5000 rpm for 5 minutes) and the supernatant was discarded. Fresh cold diethyl ether was added, and the step was repeated 4 times in total. The crude peptide was then transferred to a flask, residual diethyl ether was removed under reduced pressure and the product was dried under high vacuum.

General procedure B

- Resin swelling: 2-CTC resin ($L_{s(exp.)}$ 0.87) was swollen in DCM (enough volume to wet the resin completely) at room temperature for 30 minutes, then the solvent was discarded.
- Resin loading: 2-CTC resin was stirred overnight with a solution of Fmoc-amino acid (2 eq.) previously dissolved in a 1:2 solution of DMF/DCM and DIPEA (4 eq.).
- Resin washing: washings were performed by alternating DMF (approximately 7 mL) and DCM (approximately 7 mL) stirring the syringe for 1 minute each time; the liquid was then discarded. The washing procedure was repeated 3 times (or until the discarded liquid is transparent). A last washing in DMF was performed. In case a HPLC-MS check is required after washing, an additional treatment with DCM is required to remove all traces of DMF.

- Resin capping: the resin was stirred for 30 minutes with a solution of DMF/MeOH/DIPEA 17:2:1. The capping solution was then discarded and the resin was washed.
- Deprotection: the resin was stirred for 3 minutes with a solution of 20% piperidine in DMF. The liquid was then discarded and the process was repeated 3 times in total.
- Resin washing.
- Fmoc-AAs activation and coupling: the Fmoc-amino acid to be coupled (3 eq.) was solubilized in 5 mL of DMF/DCM 1:2, DIPEA (6 eq.) was added and the mixture was stirred until a clear solution is obtained. HATU (3 eq.) was then added and the reaction mixture was stirred until complete solubilization (no more than 10 minutes). This mixture was added to the resin and stirred for 1 h at room temperature. The coupling solution was then discarded and the resin was washed as described before. The completion of the coupling was verified by HPLC-MS. The reaction was repeated in case a partial coupling was detected.
- The procedures for deprotection and Fmoc-AA activation and coupling were repeated to complete the peptide sequence.
- Resin cleavage: after the completion of peptide sequence, the resin was washed two times (1 minute each) with DCM to remove traces of DMF. Then, 3 mL of a solution composed by 20% HFIP in DCM was added to the syringe, and the resin was swollen for 15 minutes at room temperature. The liquid was collected, and the step was repeated 3 times. Then, the solution was concentrated by evaporation under reduced pressure and the crude peptide (white) was precipitated from cold diethyl ether. The suspension was centrifugated (5000 rpm for 5 minutes) and the supernatant was discarded. Fresh cold diethyl ether was added, and this step was repeated 3 times in total. The crude peptide was then transferred to a flask, residual diethyl ether was removed under reduced pressure and the product was dried under high vacuum.

3. NMR spectra

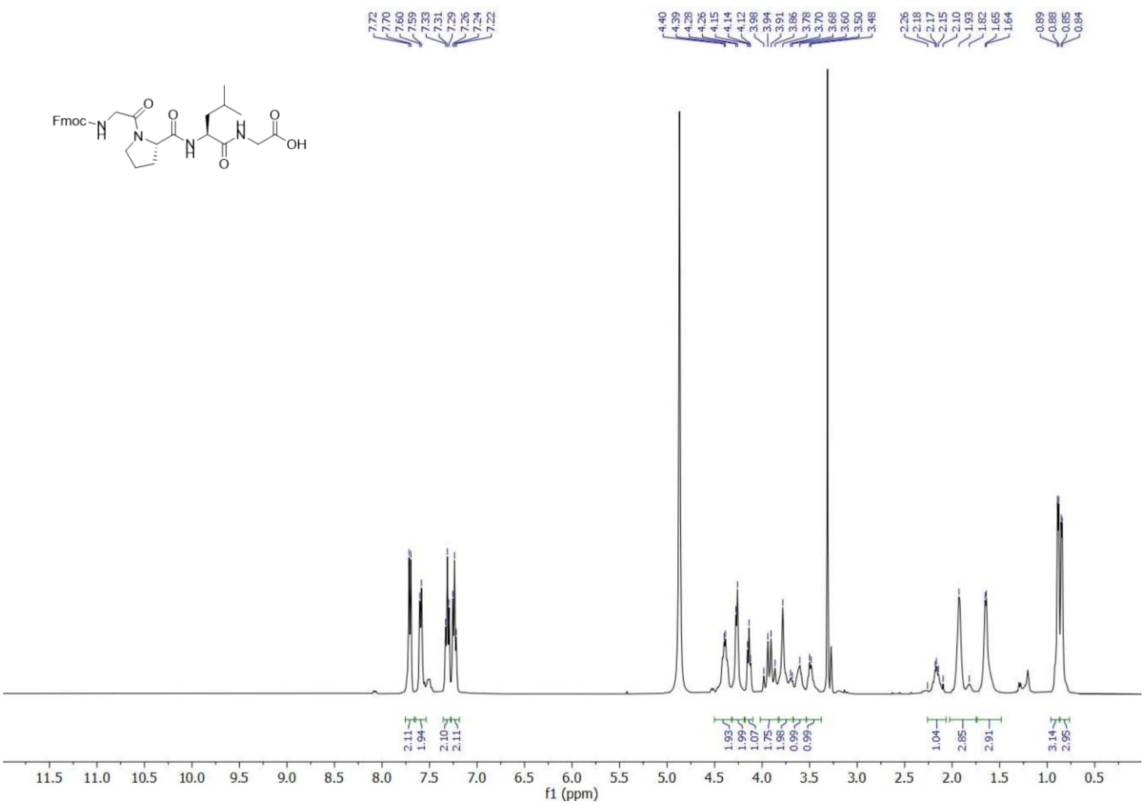

Figure S1. ^{1}H-NMR in Methanol-d_4 of Fmoc-GPLG-OH [**5a**]

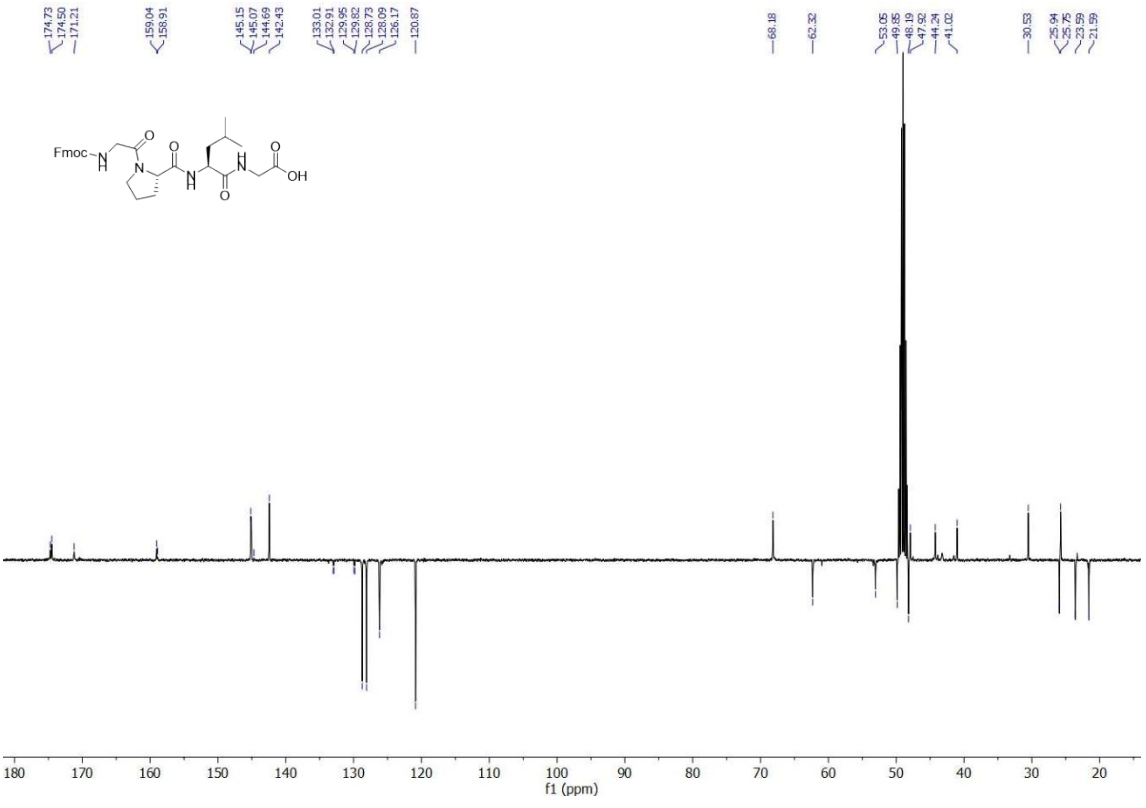

Figure S2. ^{13}C-NMR in Methanol-d_4 of Fmoc-GPLG-OH [**5a**]

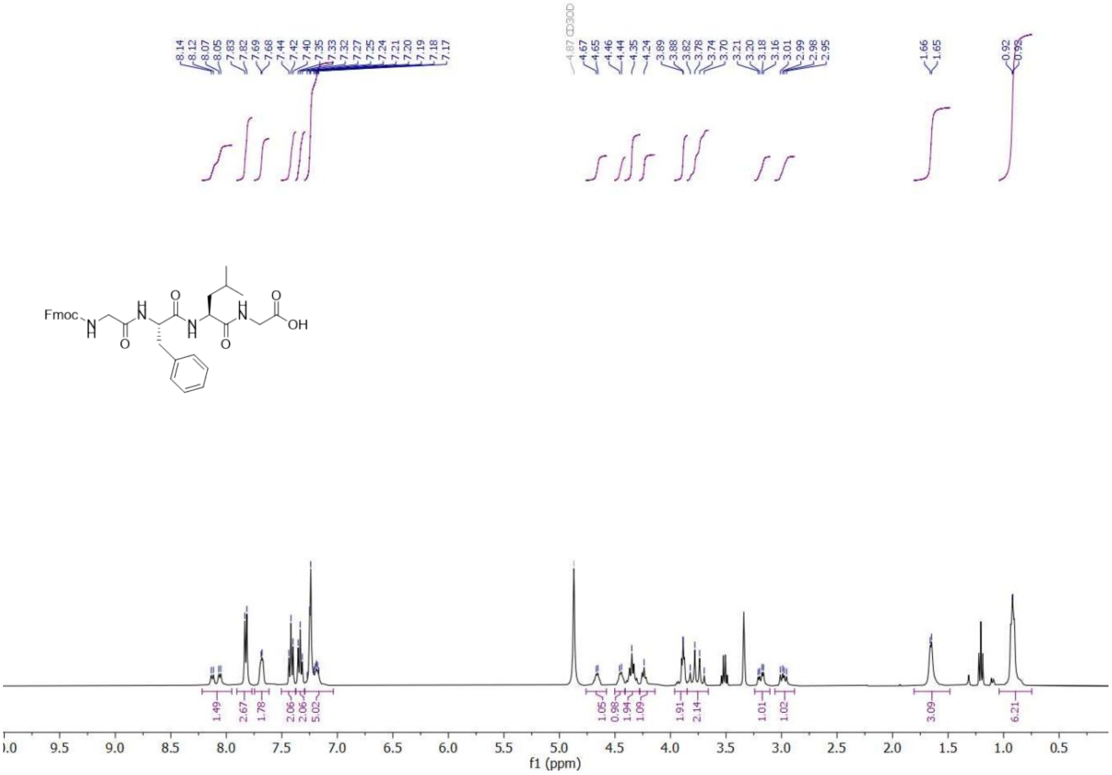

Figure S3. ^{1}H-NMR in Methanol-d_4 of Fmoc-GFLG-OH [**5b**]

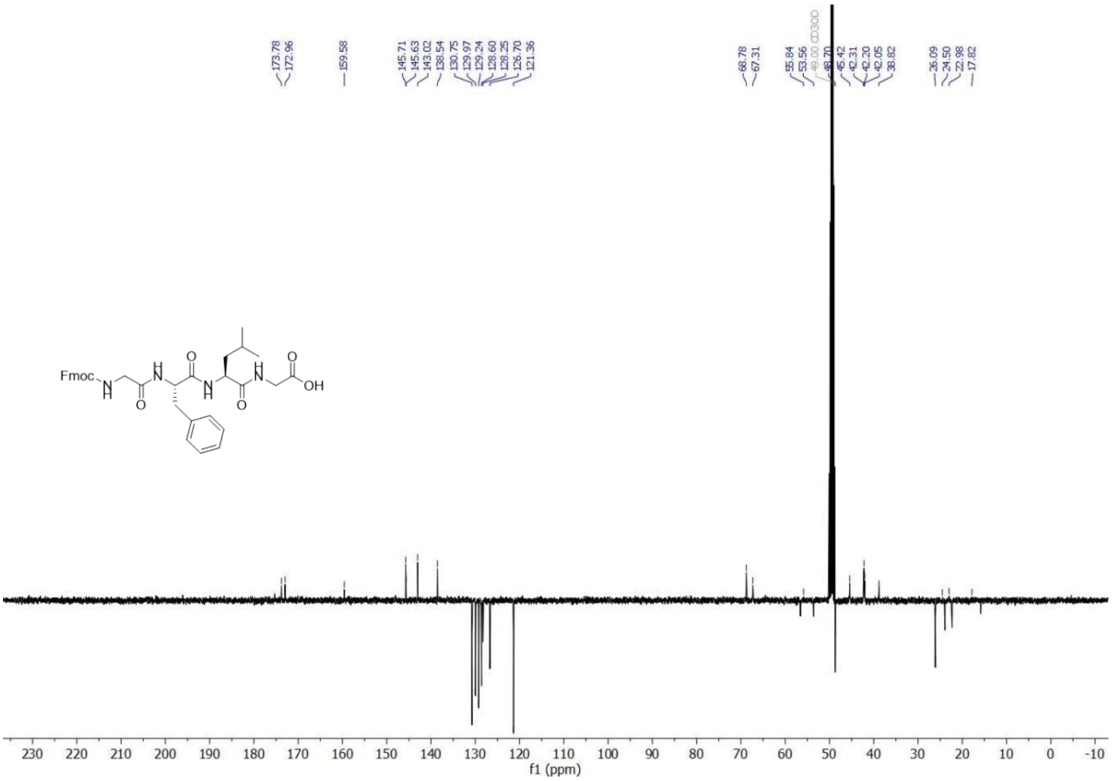

Figure S4. ^{13}C-NMR in Methanol-d_4 of Fmoc-GFLG-OH [**5b**]

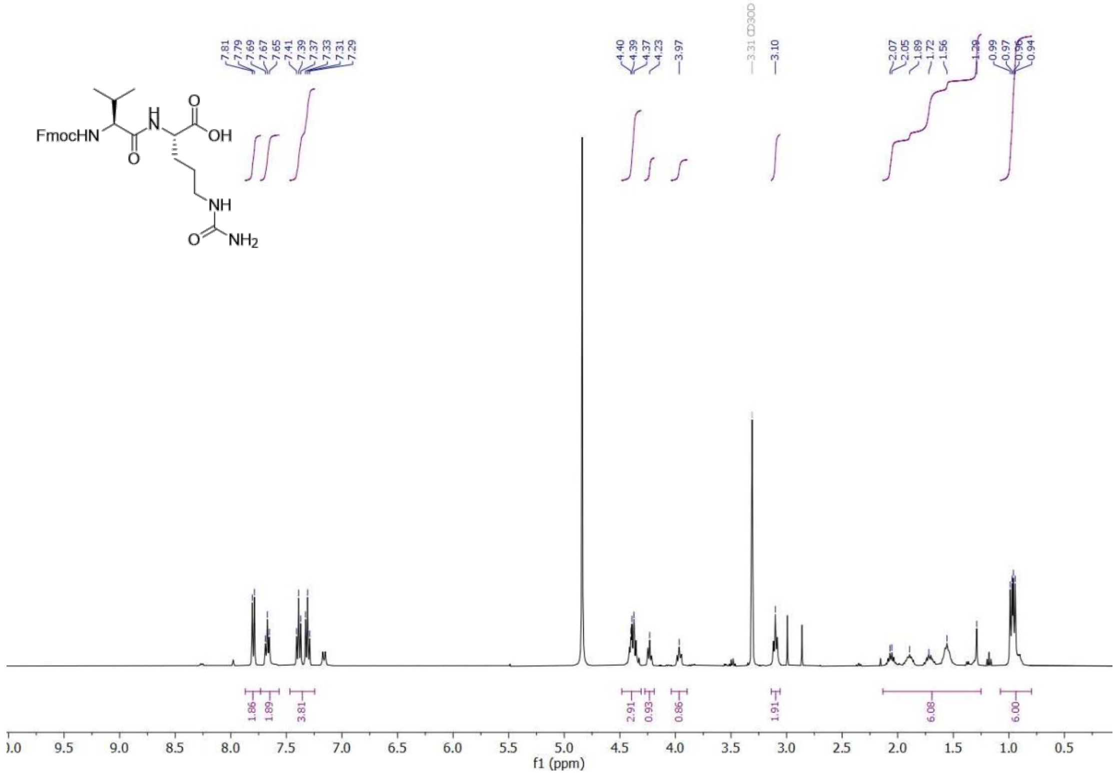

Figure S5. ^{1}H-NMR in Methanol-d_4 of Fmoc-VCit-OH [**5c**]

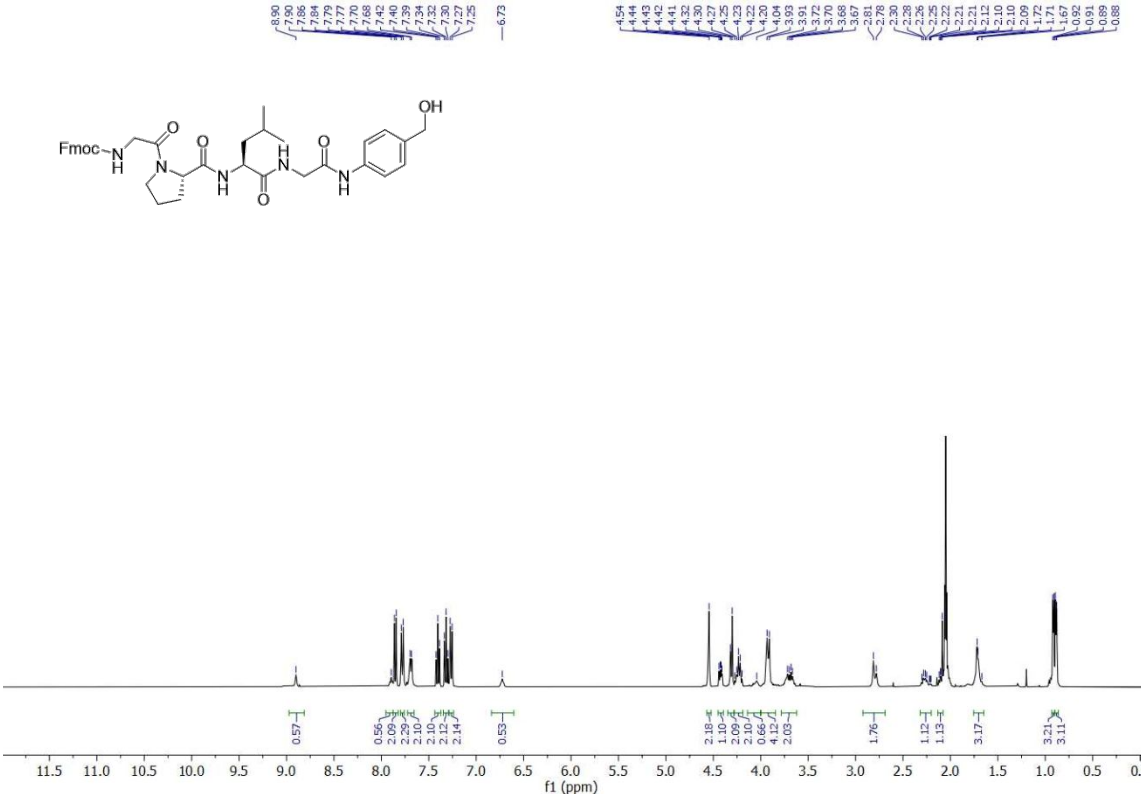

Figure S6. ^{1}H-NMR in Acetone-d_6 of Fmoc-GPLG-*N*-4-(hydroxymethyl)phenyl [**6a**]

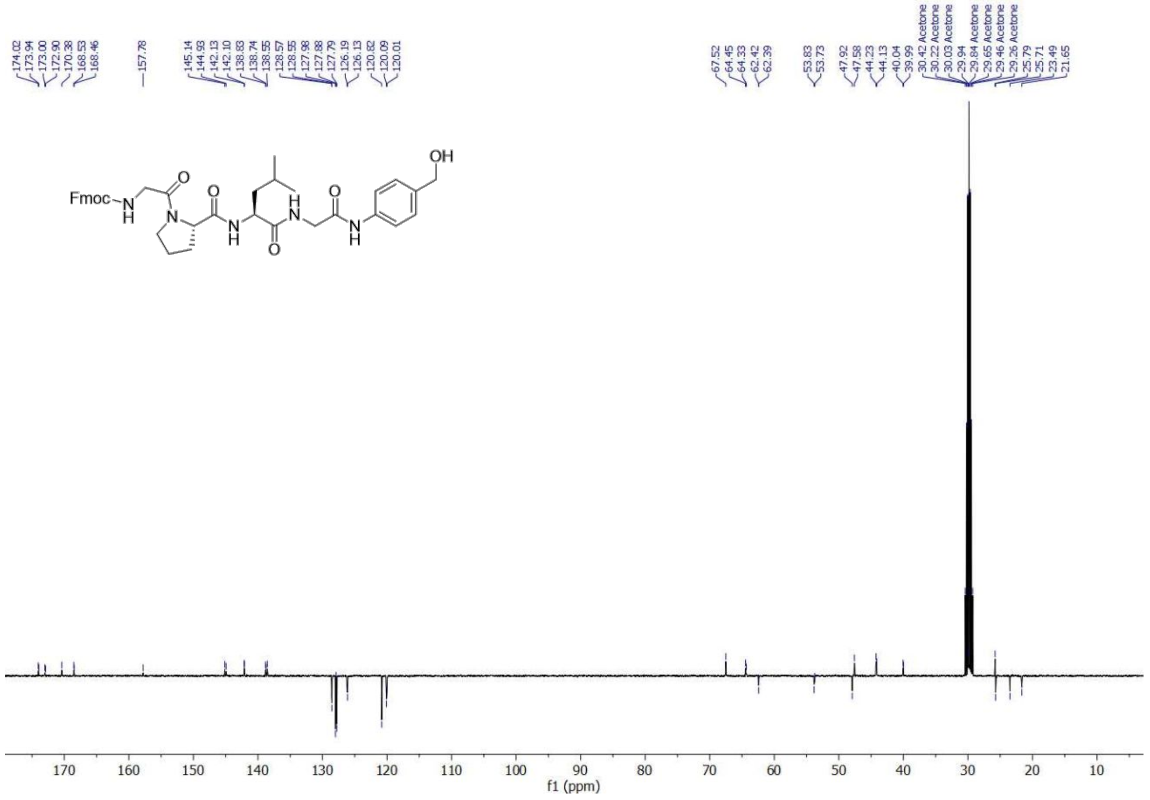

Figure S7. ^{13}C-NMR in Acetone-d_6 of Fmoc-GPLG-*N*-4-(hydroxymethyl)phenyl [**6a**]

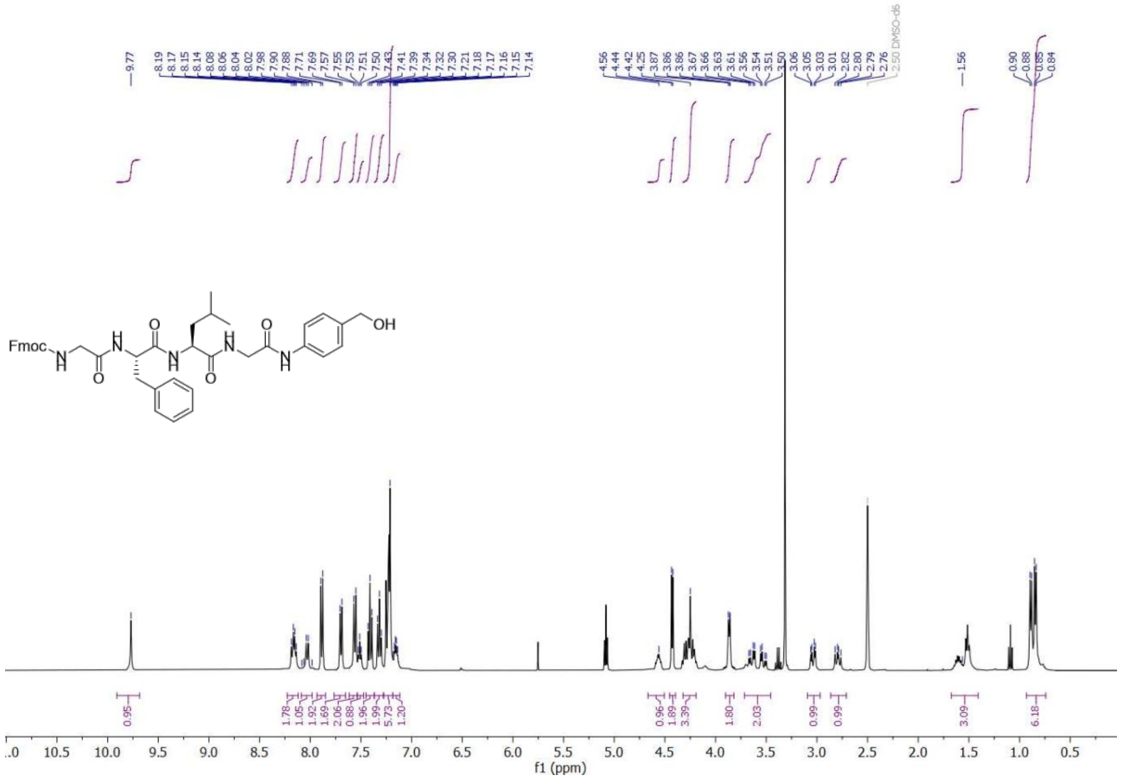

Figure S8. ^{1}H-NMR in DMSO-d_6 of Fmoc-GFLG-*N*-4-(hydroxymethyl)phenyl [**6b**]

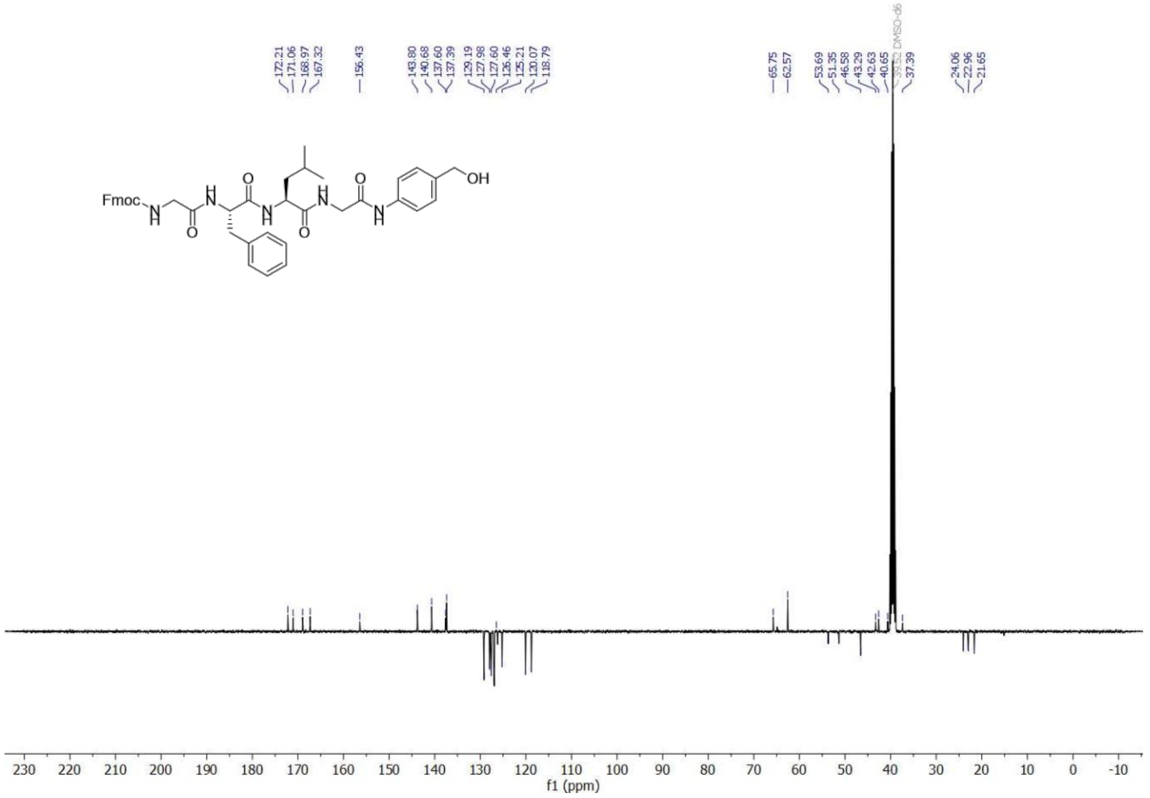

Figure S9. ^{13}C-NMR in DMSO-d_6 of Fmoc-GFLG-*N*-4-(hydroxymethyl)phenyl [**6b**]

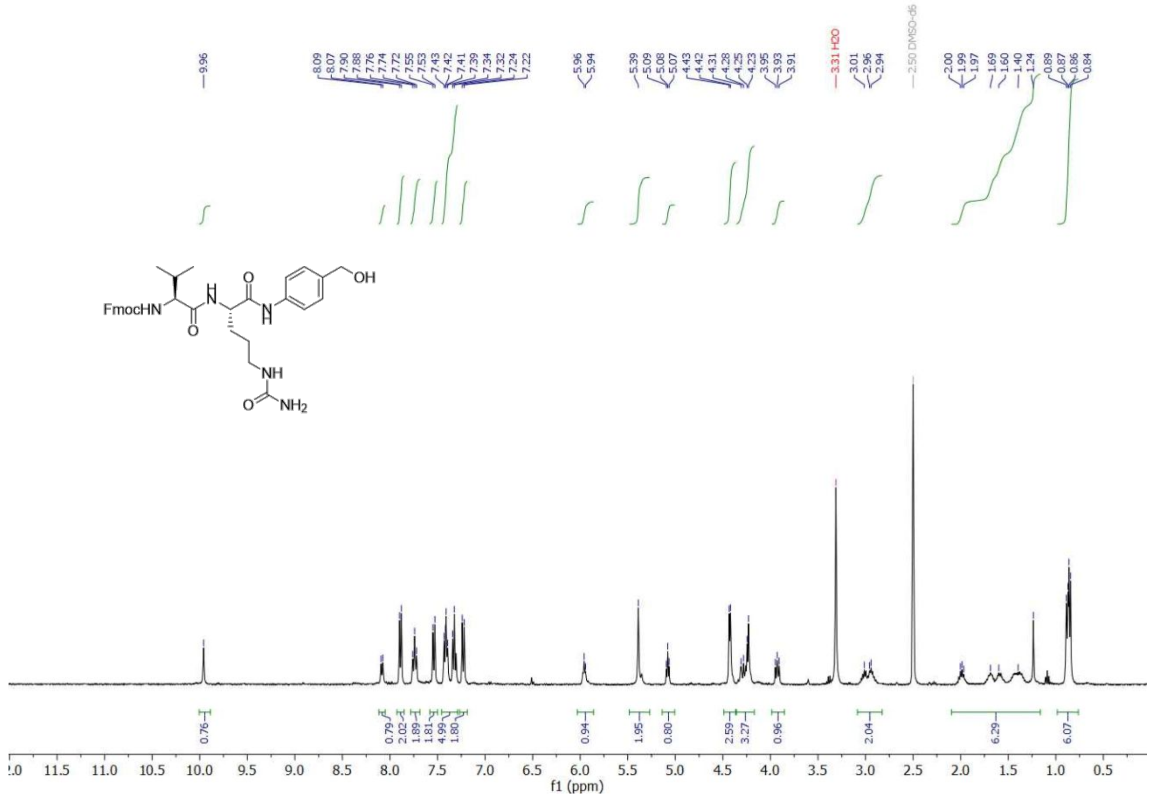

Figure S10. ^{1}H-NMR in DMSO-d_6 of Fmoc-VCit-*N*-4-(hydroxymethyl)phenyl [**6c**]

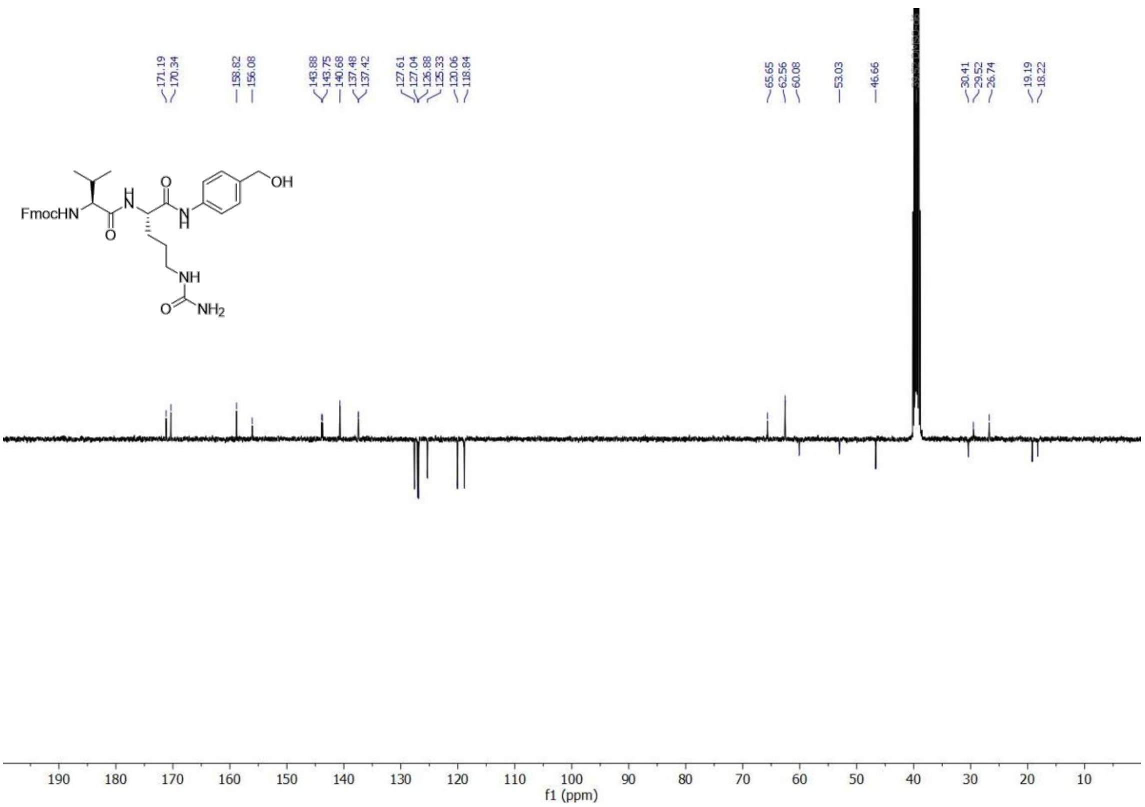

Figure S11. ^{13}C-NMR in DMSO-d_6 of Fmoc-VCit-*N*-4-(hydroxymethyl)phenyl [**6c**]

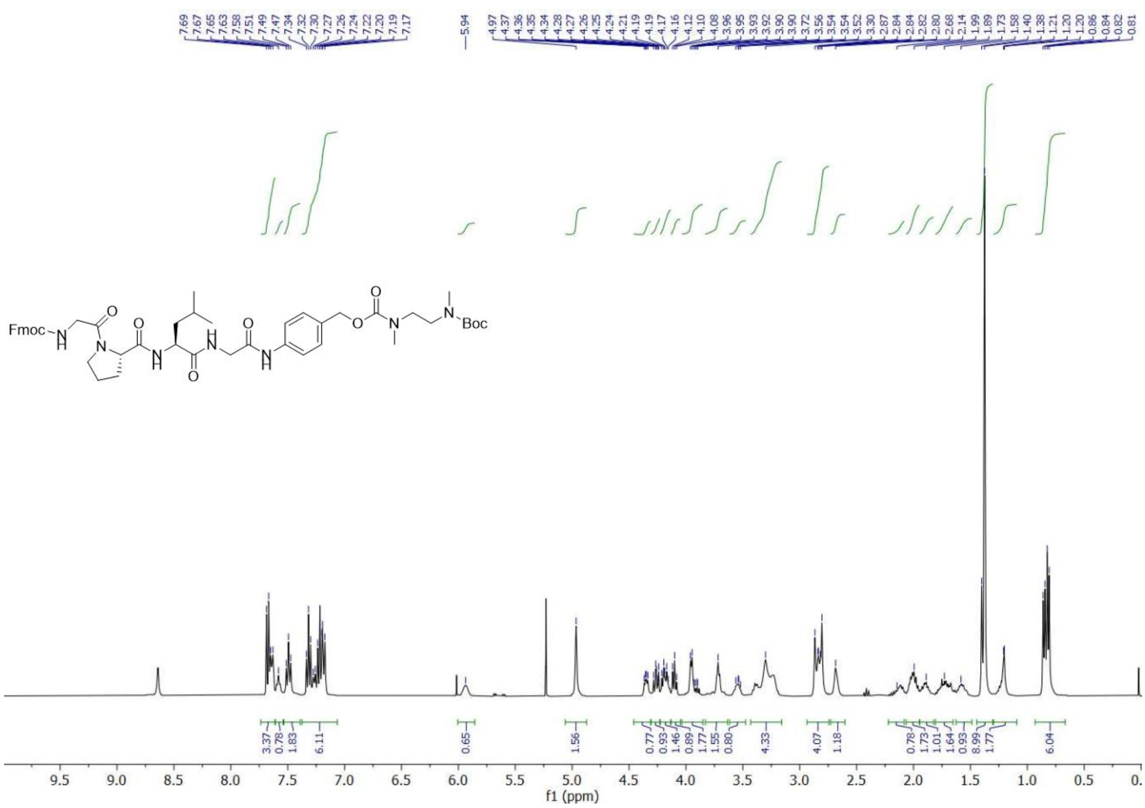

Figure S12. ^{1}H-NMR in $CDCl_3$ of Fmoc-GPLG-*N*-4-(methyloxycarbonyl-(*N*-Boc-*N,N'* dimethylethylenediamine))phenyl [**8a**]

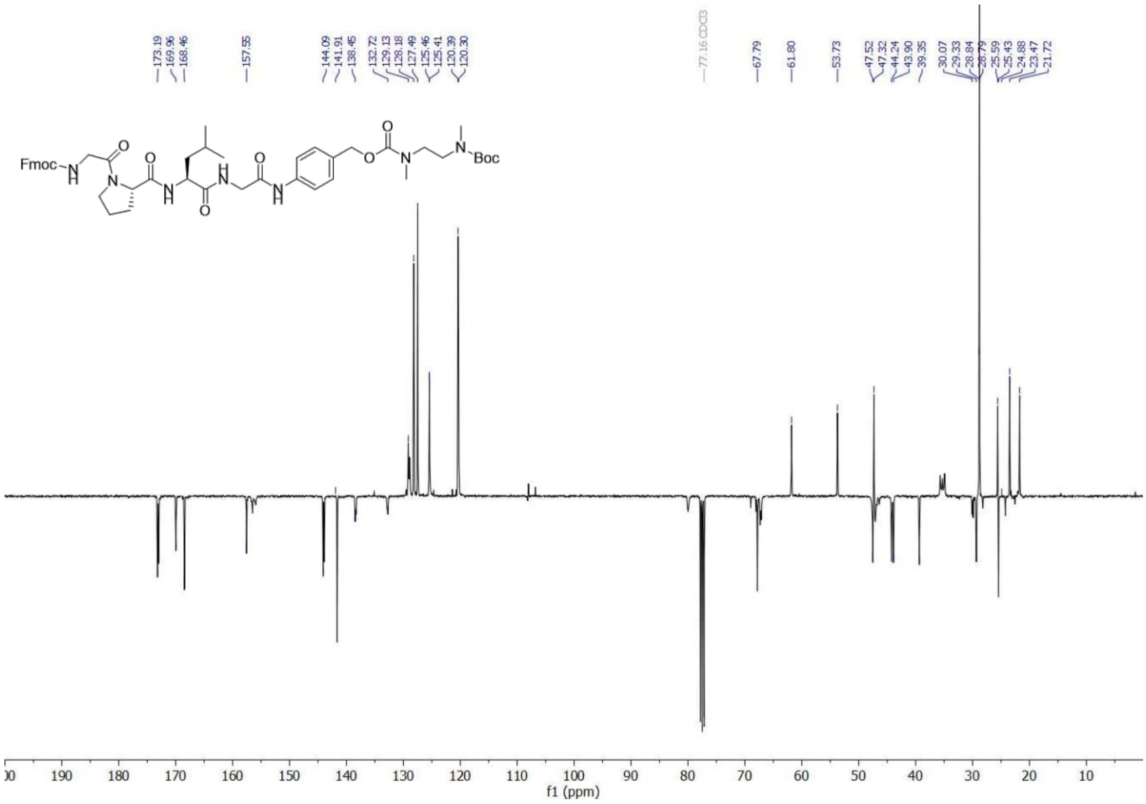

Figure S13. ^{13}C-NMR in $CDCl_3$ of Fmoc-GPLG-*N*-4-(methyloxycarbonyl-(*N*-Boc-*N,N'* dimethylethylenediamine))phenyl [**8a**]

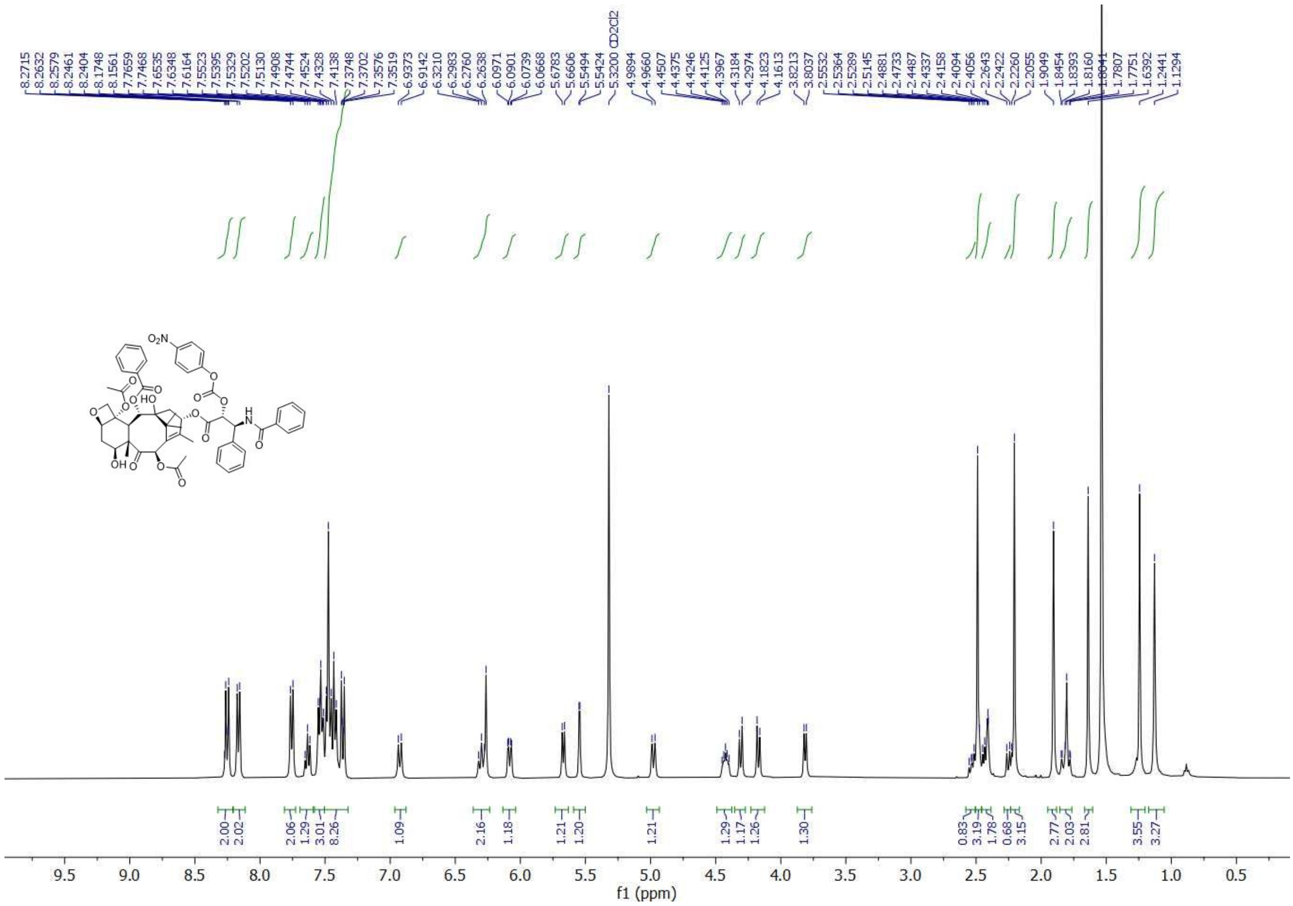

Figure S14. ^{1}H-NMR in CD_2Cl_2 of 2'-*p*NP-PTX [**10**]

4. HPLC purity analyses

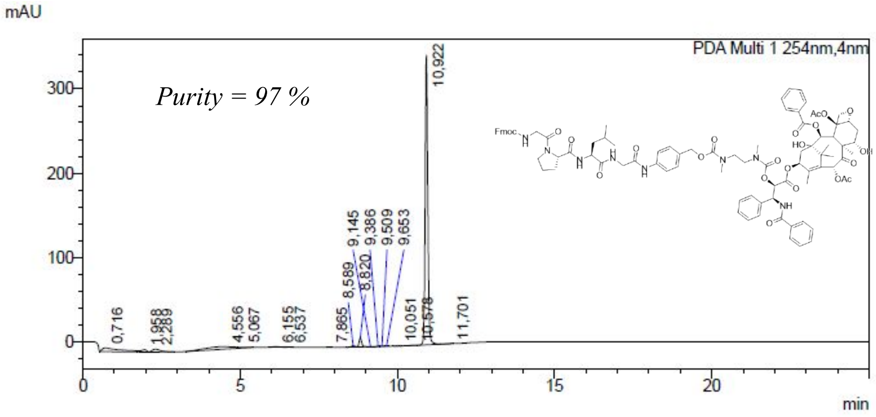

Figure S15. HPLC purity analysis of Fmoc-GPLG-PABC-*N,N'*-dimethyl-ethylene-diamine-PTX [**1**]

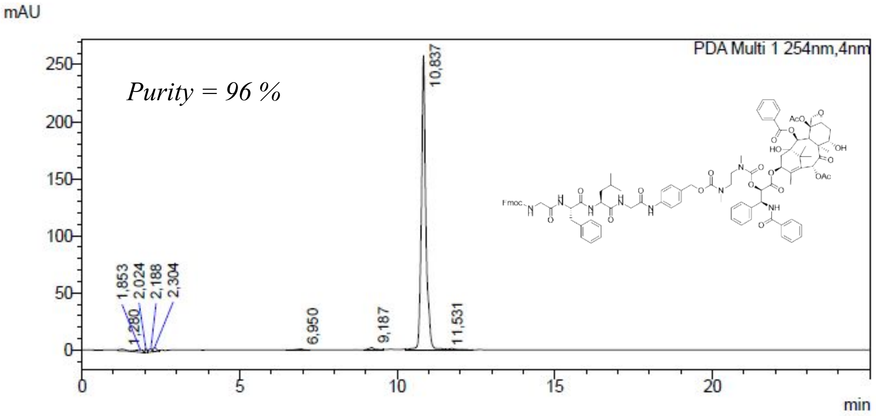

Figure S16. HPLC purity analysis of Fmoc-GFLG-PABC-*N,N'*-dimethyl-ethylene-diamine-PTX [**2**]

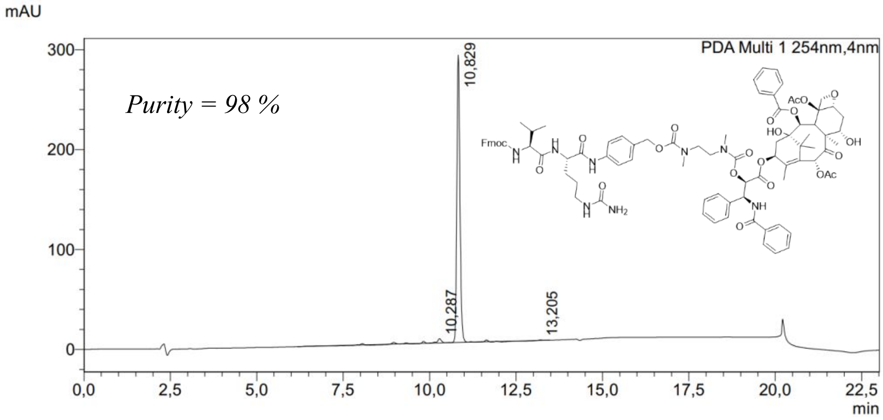

Figure S17. HPLC purity analysis of Fmoc-VCit-PABC-*N,N'*-dimethyl-ethylene-diamine-PTX [**3**]

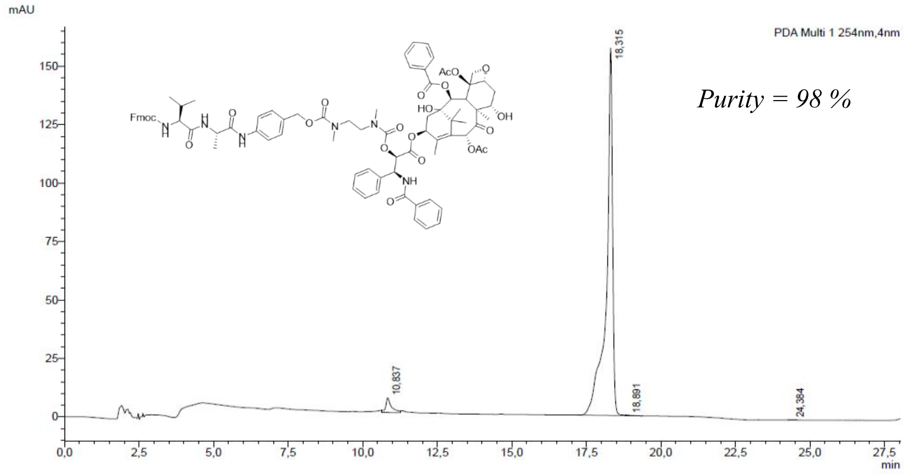

Figure S18. HPLC purity analysis of Fmoc-VA-PABC-*N,N'*-dimethyl-ethylene-diamine-PTX [**4**]

5. HRMS / LC-MS spectra

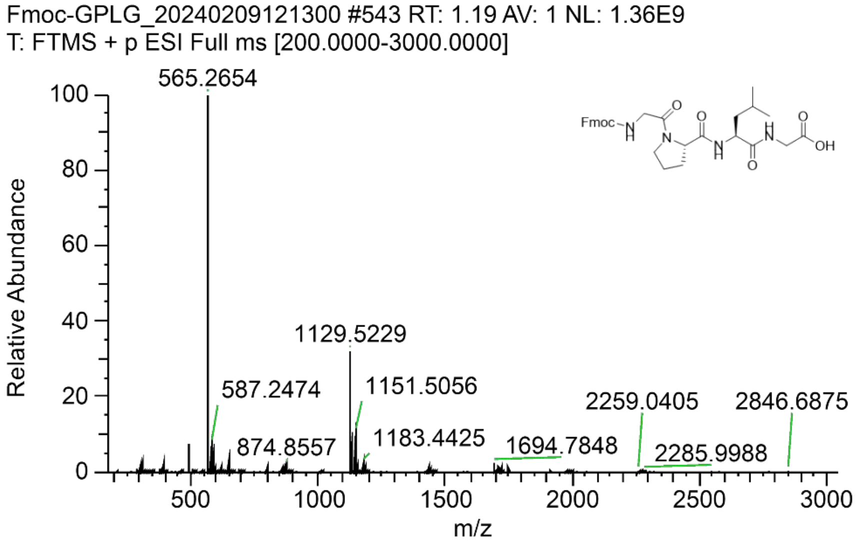

Figure S19. HRMS spectra of Fmoc-GPLG-OH [**5a**]

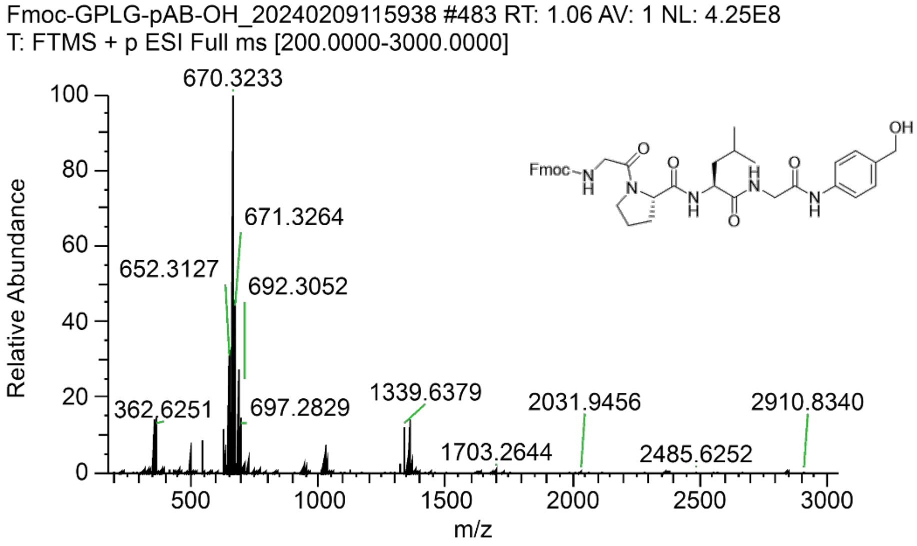

Figure S20. HRMS spectra of Fmoc-GPLG-PABOH [**6a**]

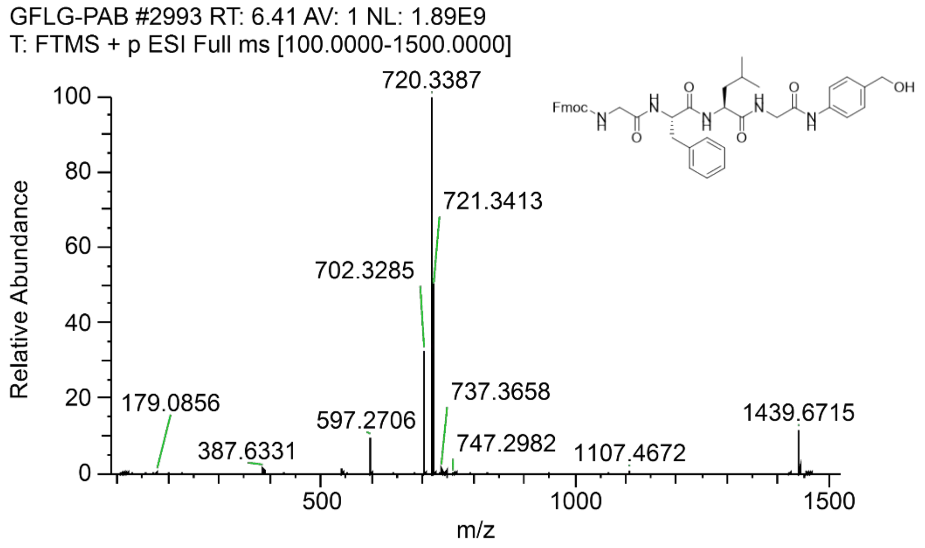

Figure S21. HRMS spectra of Fmoc-GFLG-PABOH [**6b**]

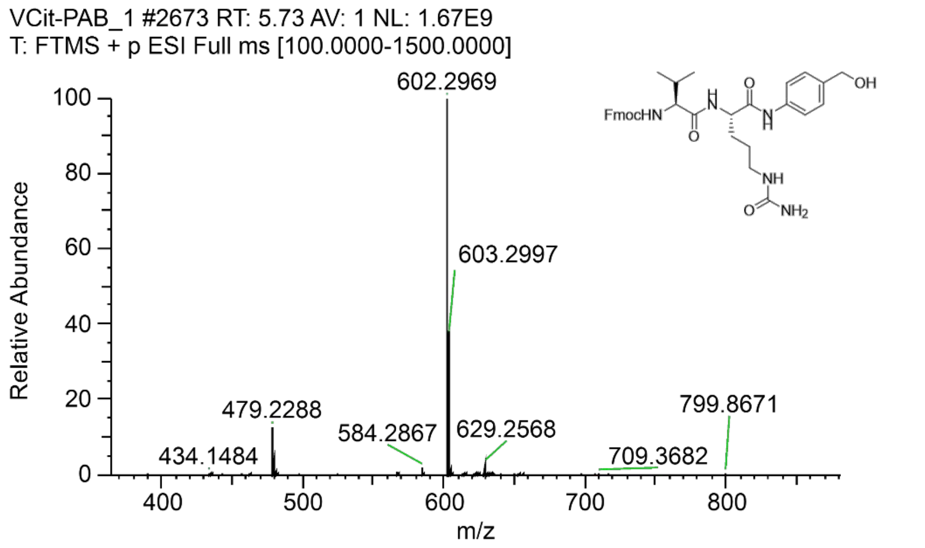

Figure S22. HRMS spectra of Fmoc-VCit-PABOH [**6c**]

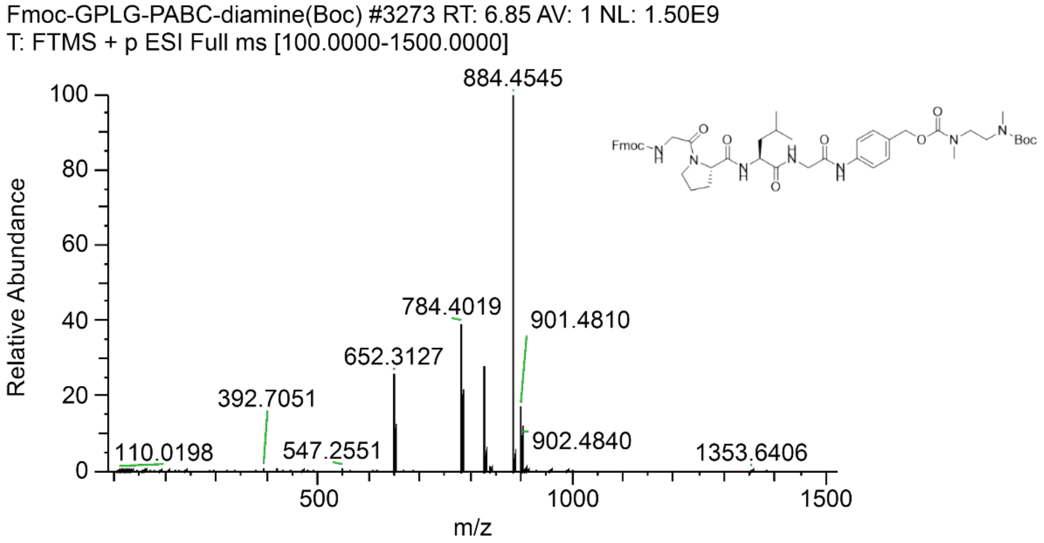

Figure S23. HRMS spectra of Fmoc-GPLG-PABC-*N*-Boc-*N,N'*-dimethyl-ethylene-diamine [**8a**]

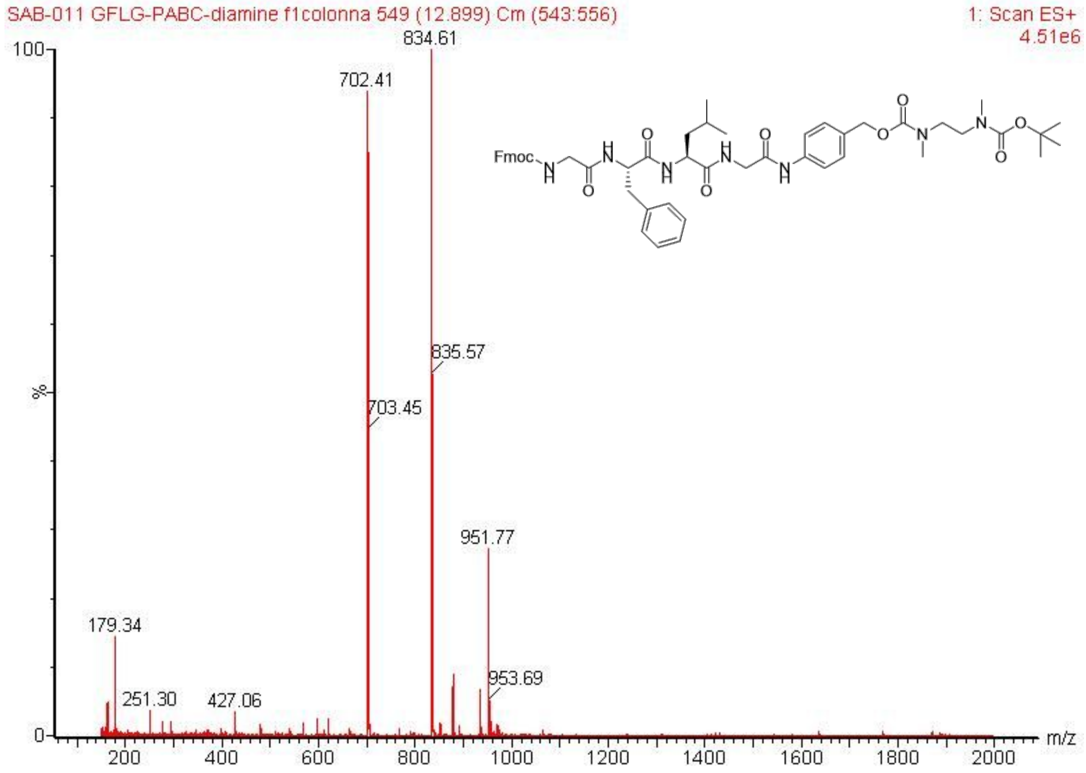

Figure S24. LC-MS spectra of Fmoc-GFLG-PABC-*N*-Boc-*N,N'*-dimethyl-ethylene-diamine [**8b**]

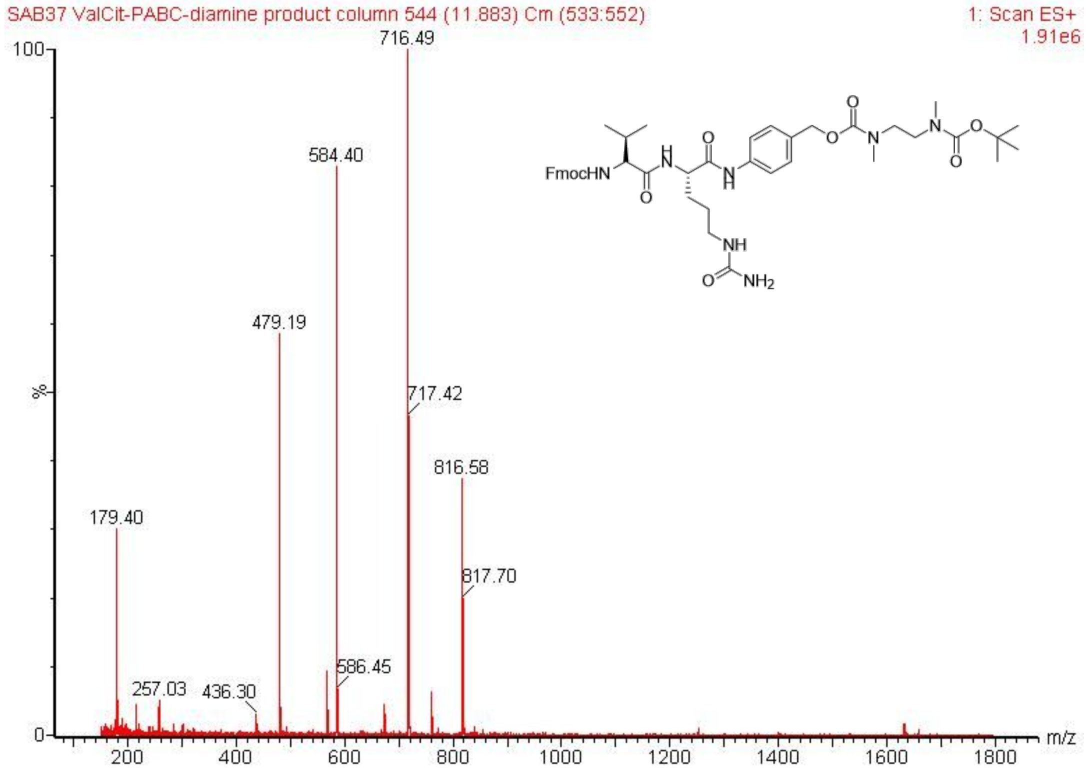

Figure S25. LC-MS spectra of Fmoc-VCit-PABC-*N*-Boc-*N,N'*-dimethyl-ethylene-diamine [**8c**]

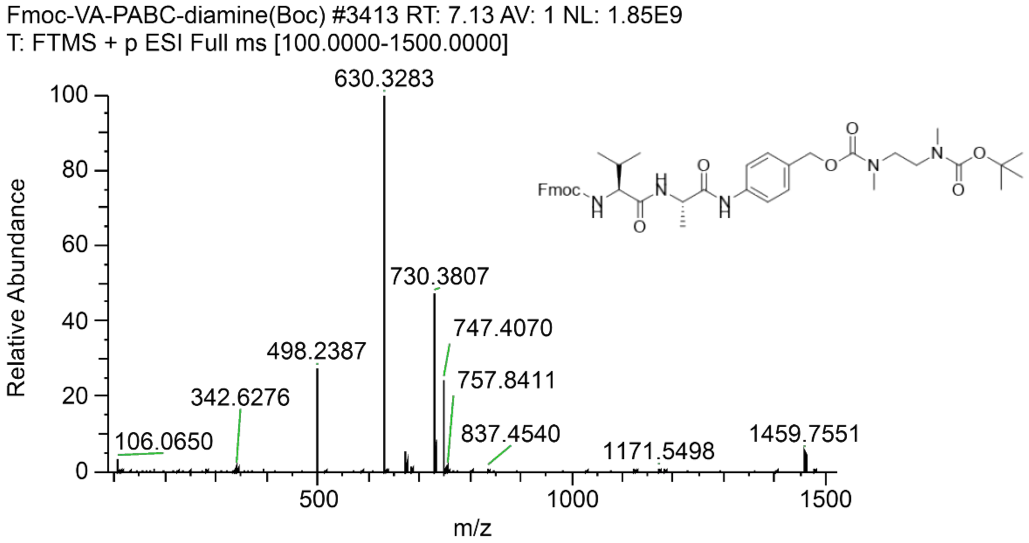

Figure S26. HRMS spectra of Fmoc-VA-PABC-*N*-Boc-*N*,*N'*-dimethyl-ethylene-diamine [**8d**]

6. Validation of the Cathepsin B cleavage assay protocol – HPLC vs UHPLC-HRMS analysis

HPLC analyses were carried out using an analytical HPLC SHIMADZU LC-20AP equipped with a diode array UV detector and a Phenomenex LC column 150x4.6 mm Synergi 4 μm Fusion RP 80 Å. A gradient system was used with the mobile phase consisting of solvent A: 90% H_20 + 10% acetonitrile + 0.1% TFA , and solvent B: 90% acetonitrile + 10% H_20. The gradient program used is shown in the following table:

Time (min)	Flow rate (mL/min)	A (%, v/v)	B (%, v/v)	Curve
Initial	1	90	10	Linear
12	1	5	95	Linear
23	1	5	95	Linear
25	1	90	10	Linear

UHPLC-MS analyses were carried out using Vanquish UHPLC System (Thermo Fisher Scientific) and Orbitrap Exploris 120 (Thermo Fisher Scientific). Thermo Scientific Xcalibur™ software was used to control the instruments and to acquire data. Freestyle 1.8™ software was used for processing the data. UHPLC chromatographic separations were performed on a reversed-phase column Accucore™ C18 50x2.1 mm, 2.6 μm (Thermo Fisher Scientific). A gradient system was used with the mobile phase consisting of solvent A: H_20 + 0.05% formic acid, and solvent B: acetonitrile. The gradient program used is shown in the following table.

Time (min)	Flow rate (mL/min)	A (%, v/v)	B (%, v/v)	Curve
Initial	0.3	90	10	Linear
2	0.3	90	10	Linear
7	0.3	0	100	Linear
12	0.3	0	100	Linear
18	0.3	90	10	Linear
19	0.3	90	10	Linear

The column was thermostated at 30 °C and the sample temperature was set at 25 °C. The MS parameters are shown in the following table:

Ion Source Type	H-ESI
Spray Voltage	Static
Positive Ion (V)	3300
Negative Ion (V)	3200
Sheat Gas (arb)	40
Aux Gas (arb)	10
Ion Transfer Tube (°C)	280
Vaporizer Temp (°C)	150

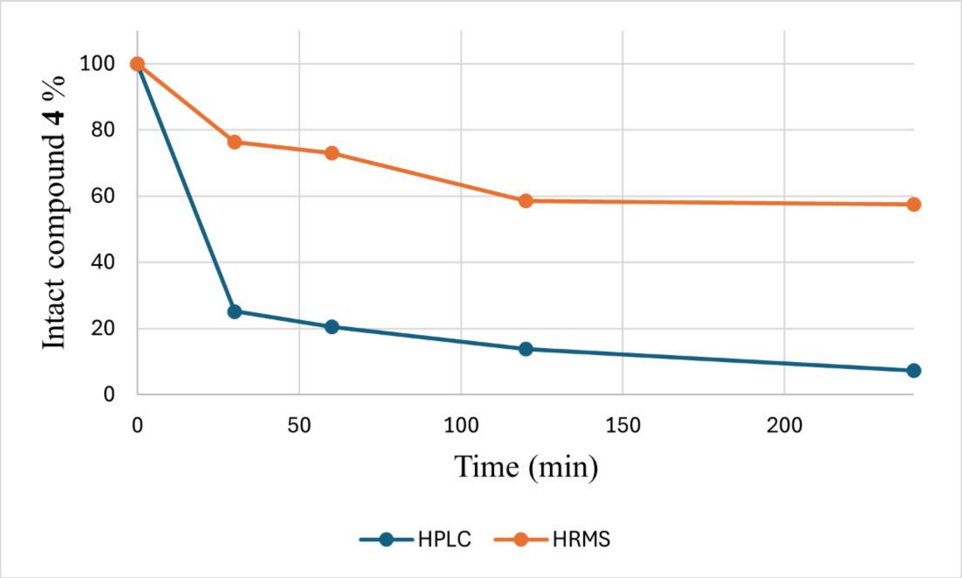

Figure S27. Comparison of the preliminary Cathepsin B cleavage assay of compound **4** performed analyzing the same assay buffer by UHPLC-HRMS and HPLC

Table S1. HRMS and HPLC data of the preliminary Cathepsin B cleavage assays of compound **4**.

	**% residual compound 2**	
**Time (min.)**	**HRMS**	**HPLC**
0	100	100
30	73.35	25.11
60	68.45	20.48
120	65.40	13.68
240	63.70	7.26

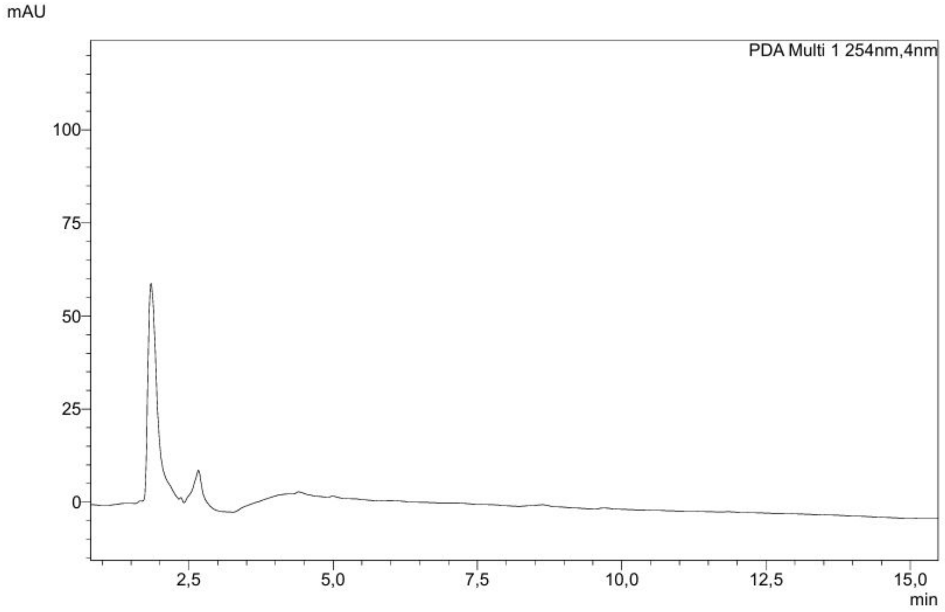

Figure S28. HPLC chromatogram of the black sample containing only the sodium acetate buffer solution.

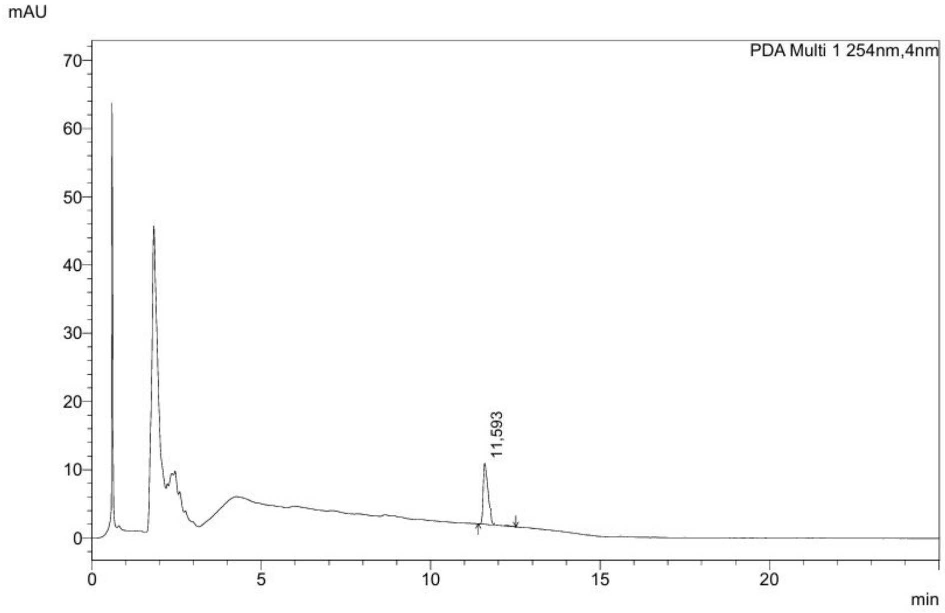

PDA Ch1 254nm

Peak#	Ret. Time	Area	Height	Conc.	Unit	Mark	Name
1	11,593	112432	8954	0,000		M	
Total		112432	8954				

Figure S29. HPLC chromatogram and the corresponding peak table of the cathepsin B cleavage assay of compound **4** after 30 min of incubation.

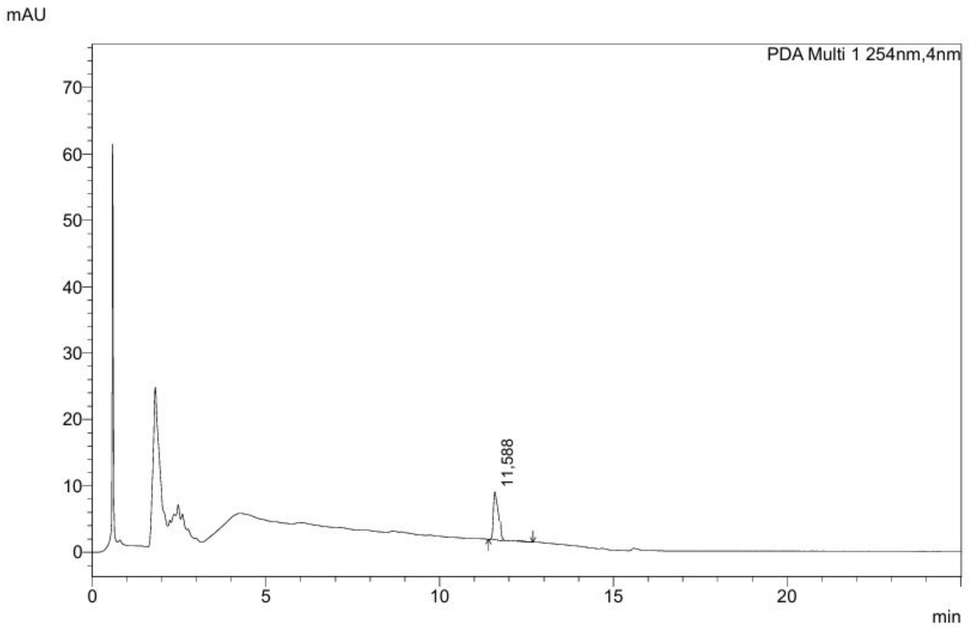

PDA Ch1 254nm

Peak#	Ret. Time	Area	Height	Conc.	Unit	Mark	Name
1	11,588	91689	7212	0,000		M	
Total		91689	7212				

Figure S30. HPLC chromatogram and the corresponding peak table of the cathepsin B cleavage assay of compound **4** after 1 hour of incubation.

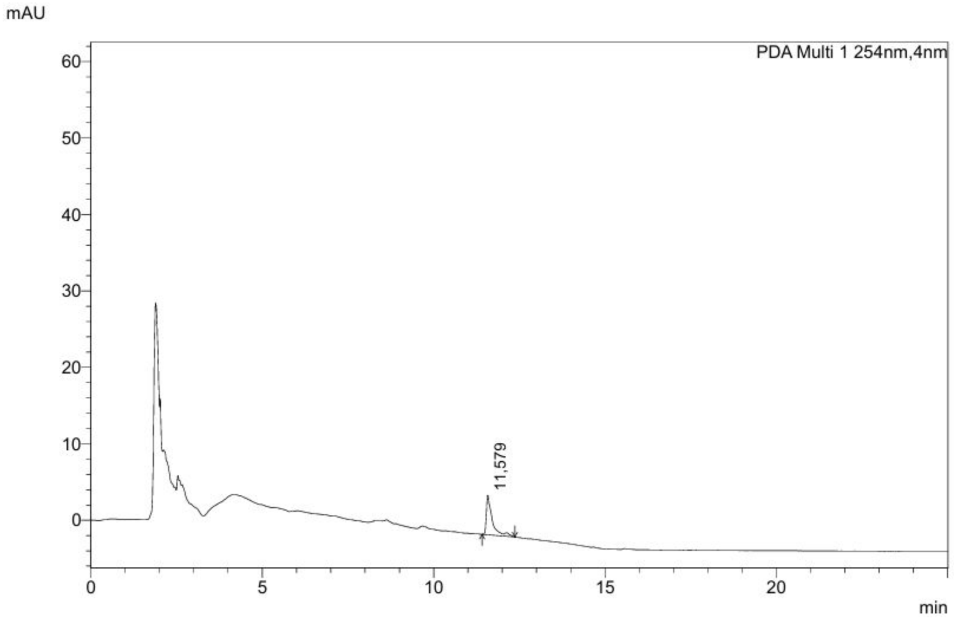

PDA Ch1 254nm

Peak#	Ret. Time	Area	Height	Conc.	Unit	Mark	Name
1	11,579	61251	5162	0,000		M	
Total		61251	5162				

Figure S31. HPLC chromatogram and the corresponding peak table of the cathepsin B cleavage assay of compound **4** after 2 hours of incubation.

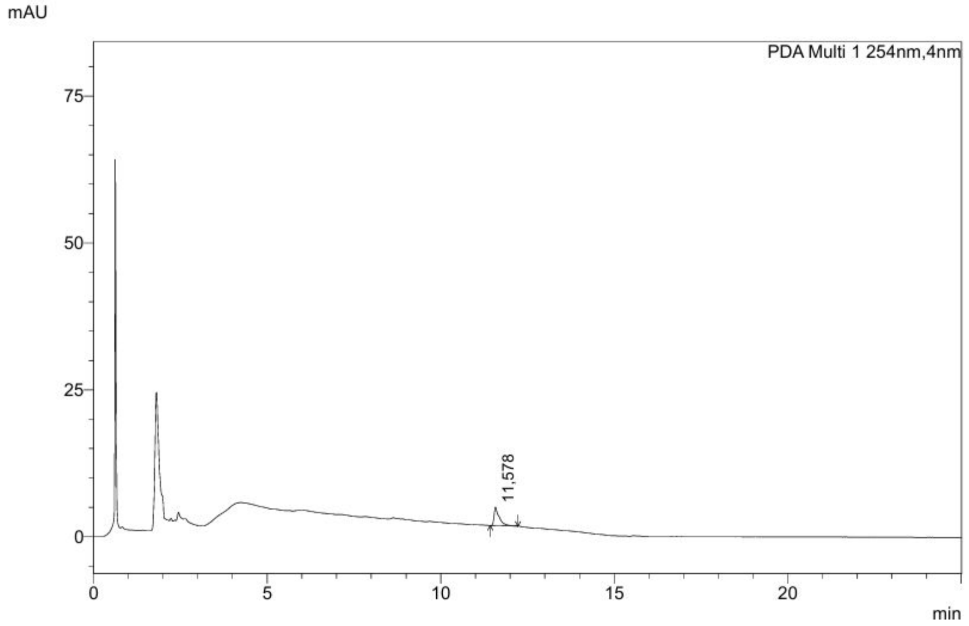

PDA Ch1 254nm

Peak#	Ret. Time	Area	Height	Conc.	Unit	Mark	Name
1	11,578	32479	3110	0,000		M	
Total		32479	3110				

Figure S32. HPLC chromatogram and the corresponding peak table of the cathepsin B cleavage assay of compound **4** after 4 hours of incubation.

Figure S33. Cathepsin B cleavage assay – UPLC-HRMS analysis

7. Cathepsin B cleavage assay – UHPLC-HRMS analysis

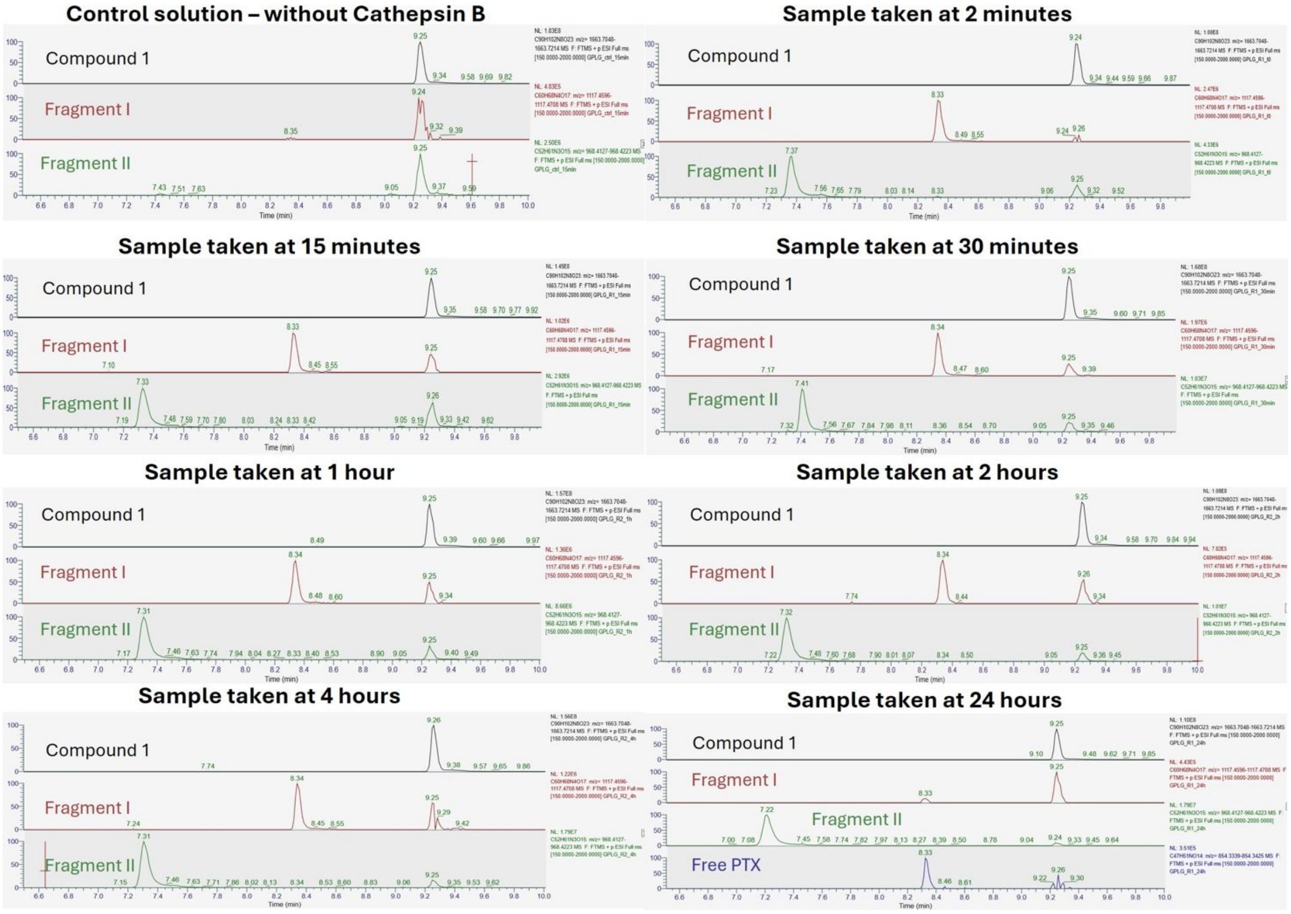

Figure S34. EICs obtained from Cathepsin B assay - compound **1**

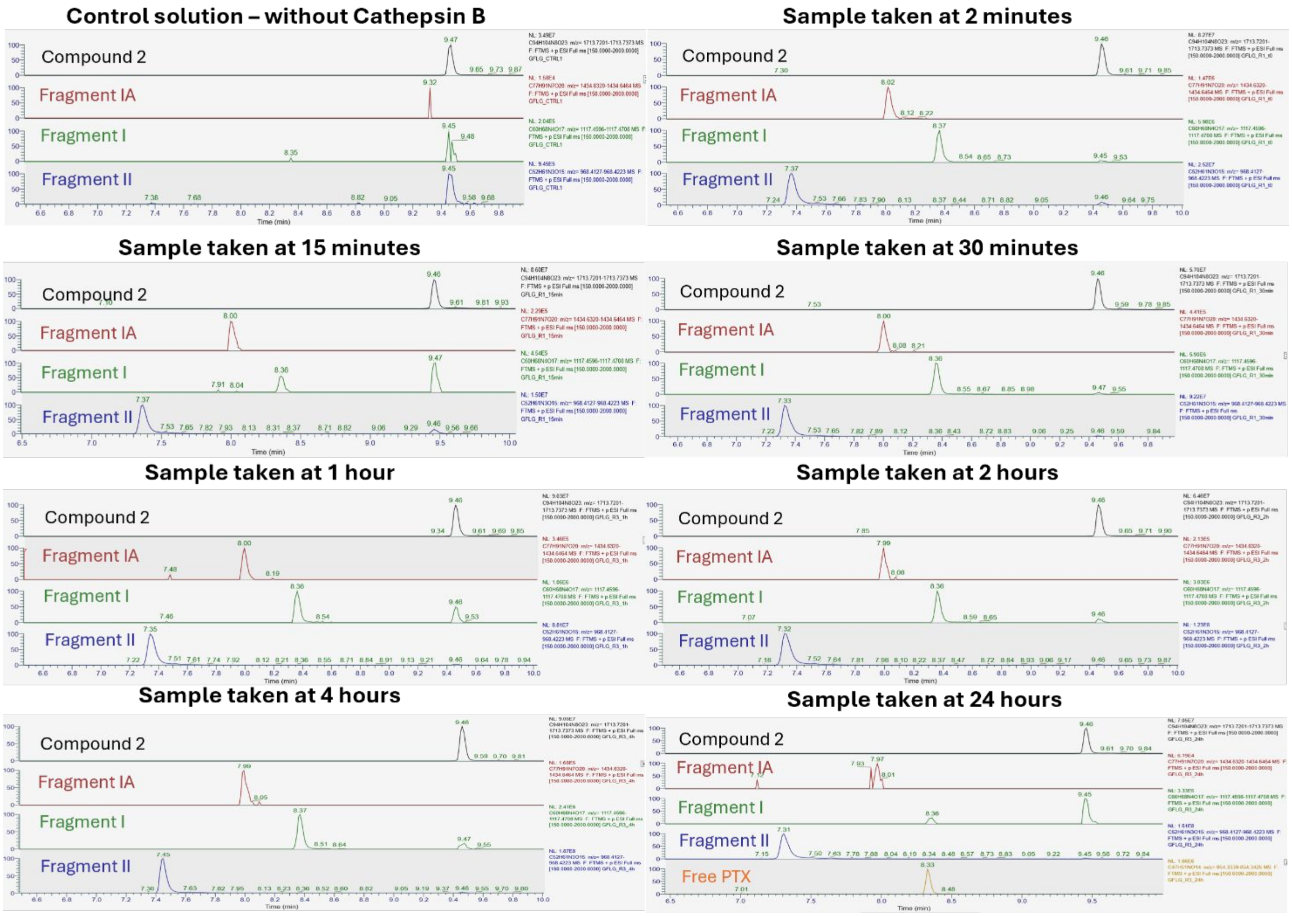

Figure S35. EICs obtained from Cathepsin B assay - compound **2**

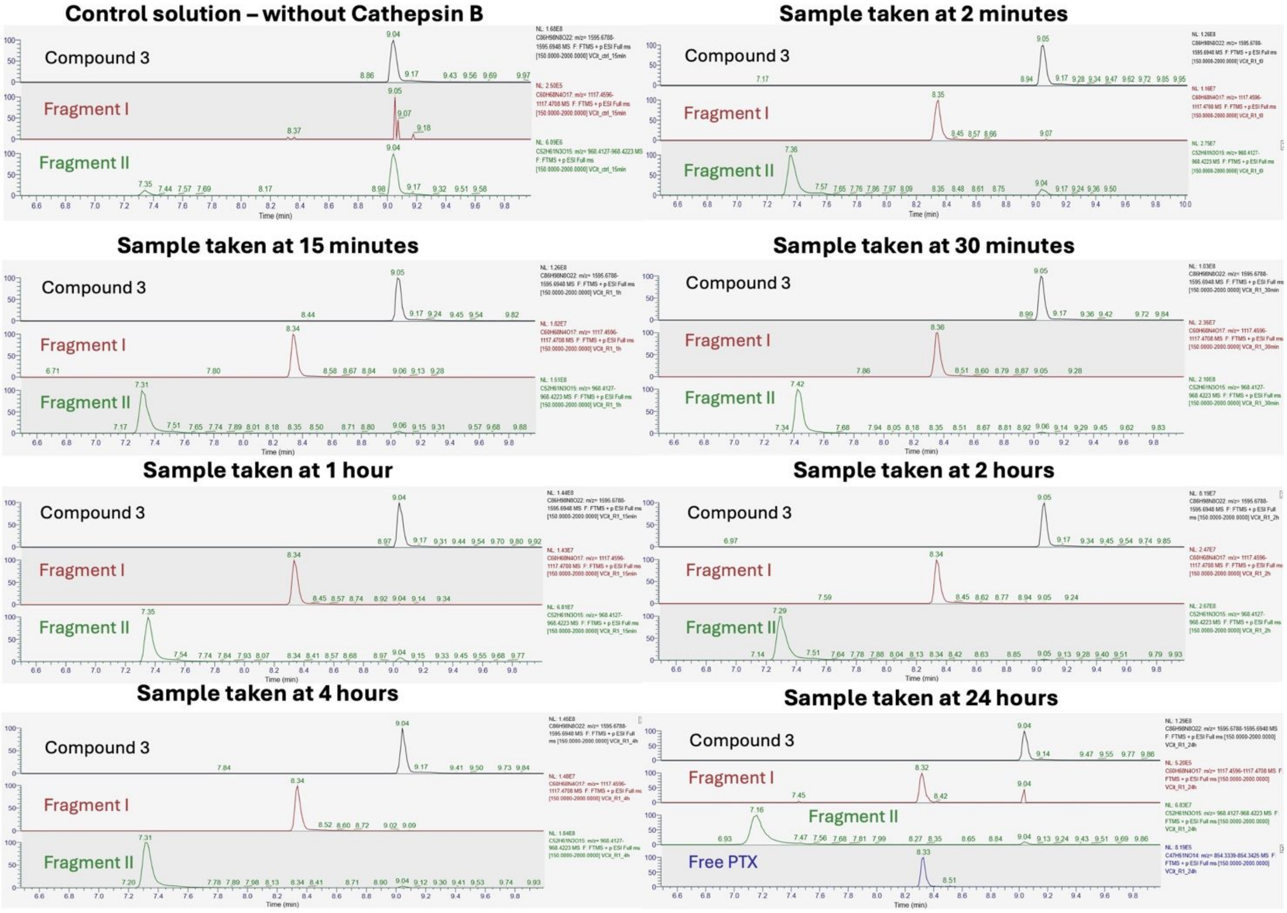

Figure S36. EICs obtained from Cathepsin B assay - compound **3**

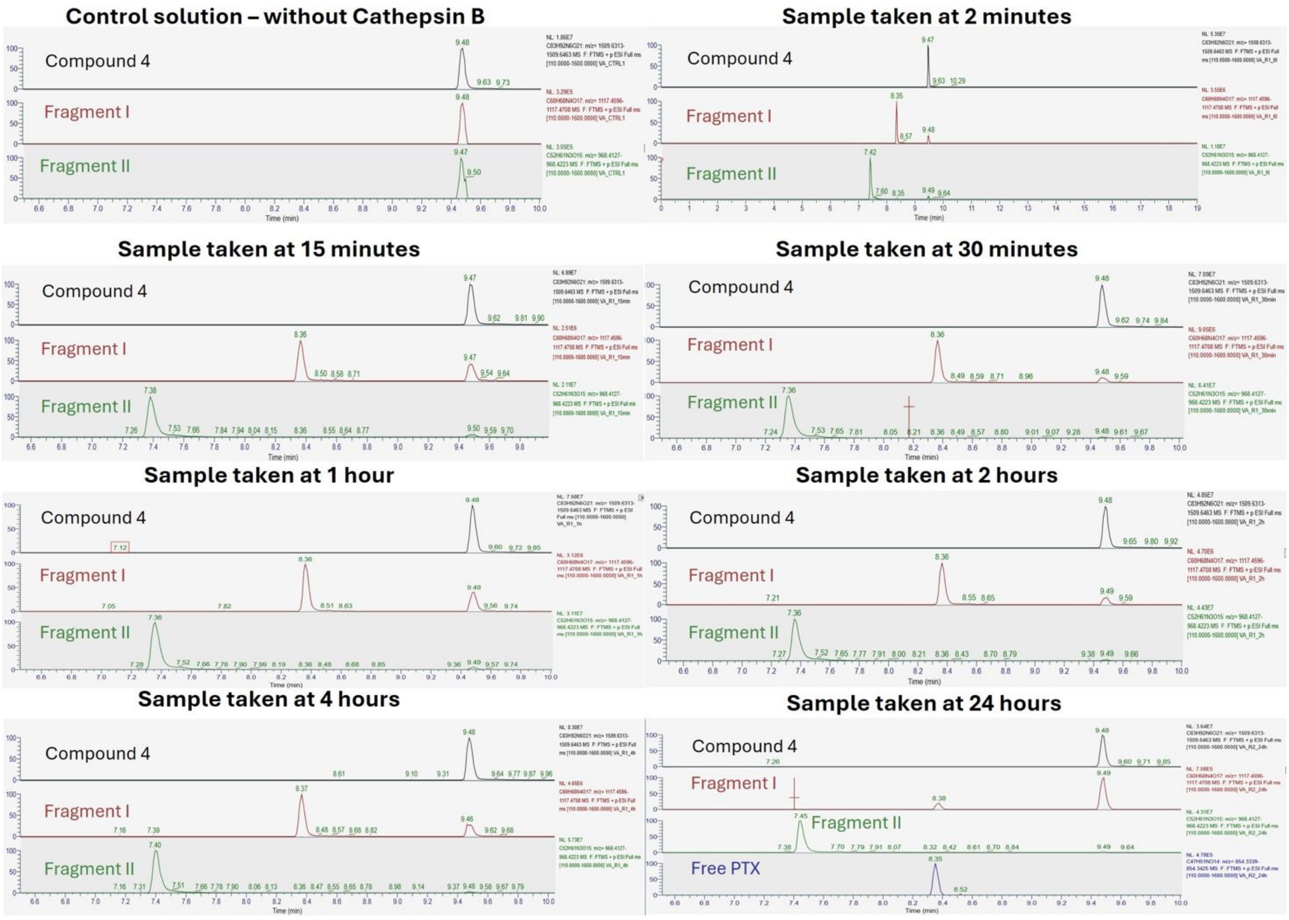

Figure S37. EICs obtained from Cathepsin B assay - compound **4**

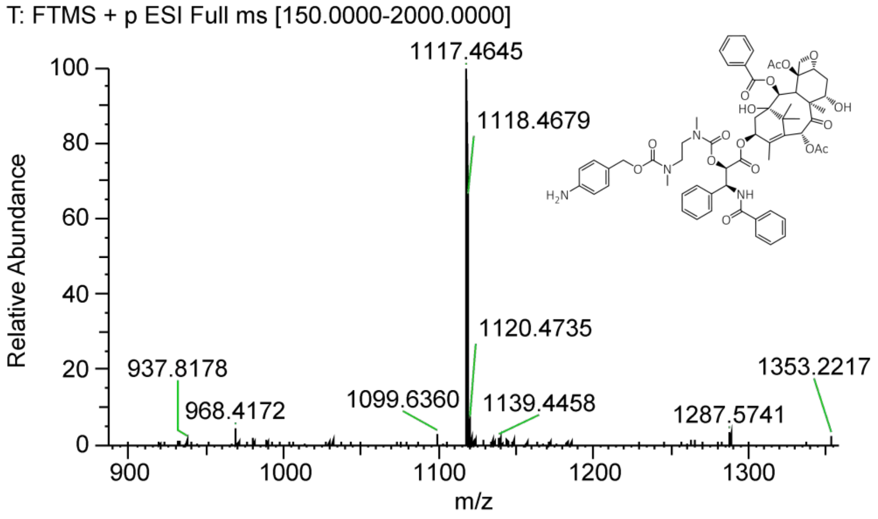

Figure S38. HRMS spectrum of Fragment **I**

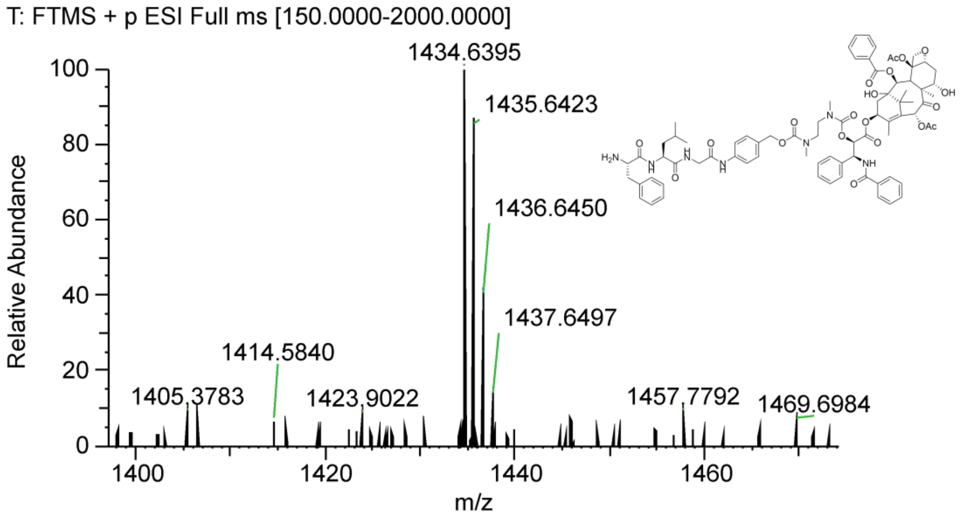

Figure S39. HRMS spectrum of Fragment **IA**

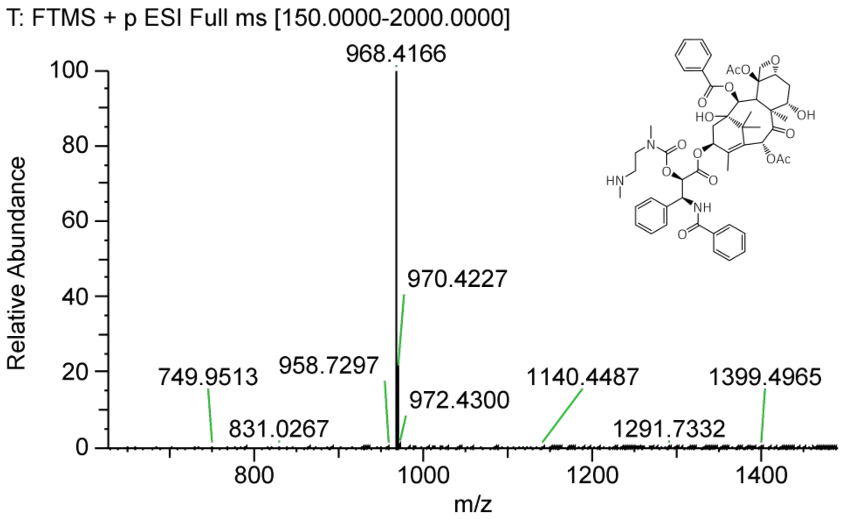

Figure S40. HRMS spectrum of Fragment **II**

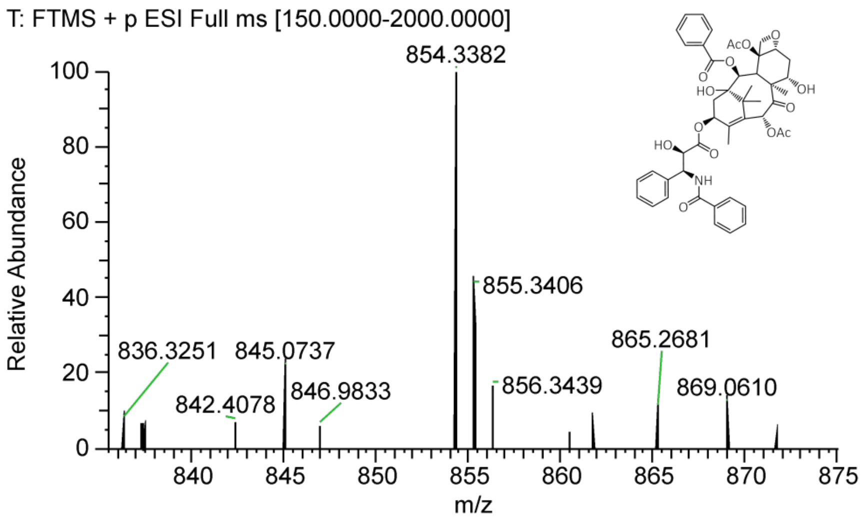

Figure S41. HRMS spectrum of free PTX

Conjugate **1**	
m/z calculated for $[C_{54}H_{54}N_2O_{18}]^+$: 1663.71306 $[M+H]^+$	*m/z* obtained: 1663.7113 $[M+H]^+$
Conjugate **2**	
m/z calculated for $[C_{54}H_{54}N_2O_{18}]^+$: 1713.72871 $[M+H]^+$	*m/z* obtained: 1713.7284 $[M+H]^+$
Conjugate **3**	
m/z calculated for $[C_{54}H_{54}N_2O_{18}]^+$: 1595.68684 $[M+H]^+$	*m/z* obtained: 1595.6869 $[M+H]^+$
Conjugate **4**	

m/z calculated for $[C_{54}H_{54}N_2O_{18}]^+$: 1509.63883 $[M+H]^+$	*m/z* obtained: 1595.63834 $[M+H]^+$
Fragment **I**	
m/z calculated for $[C_{60}H_{68}N_4O_{17}]^+$: 1117.46522 $[M+H]^+$	*m/z* obtained: 1117.4642 $[M+H]^+$
Fragment **IA**	
m/z calculated for $[C_{77}H_{91}N_7O_{20}]^+$: 1434.63916 $[M+H]^+$	*m/z* obtained: 1434.6395 $[M+H]^+$
Fragment **II**	
m/z calculated for $[C_{52}H_{61}N_3O_{15}]^+$: 968.41754 $[M+H]^+$	*m/z* obtained: 968.4166 $[M+H]^+$
Free PTX	
m/z calculated for $[C_{47}H_{51}NO_{14}]^+$: 854.3382 $[M+H]^+$	*m/z* obtained: 854.3382 $[M+H]^+$

Table S2. HRMS data of the Cathepsin B cleavage assays of compound **1**.

Pick Area Compound 1					
**Time (min.)**	**R1**	**R2**	**R3**		
2	5560326708	3939288116	4966171856		
15	3766992334	3995417944	3881205139		
30	3778568504	3677746343	3674353313		
60	3508675152	3453127791	3527320915		
120	3550542293	3045568588	3425930473		
240	2865212096	3270768639	3625382412		
**Area Ctrl T0**	5108201948				
**Time (min.)**	**% R1**	**% R2**	**% R3**	**Average %**	**Dv. St.**
0	100	100	100	100	0
2	108.85	77.12	97.22	94.40	16.05
15	85.04	78.22	78.25	80.50	3.93
30	73.97	72.00	74.08	73.35	1.17
60	68.69	67.60	69.05	68.45	0.76
120	69.51	59.62	67.07	65.40	5.15
240	56.09	64.03	70.97	63.70	7.45

Table S3. HRMS data of the Cathepsin B cleavage assays of compound **2**.

Pick Area Compound 2					
**Time (min.)**	**R1**	**R2**	**R3**		
2	2515268257	2409938709	2643519958		
15	2423324505	2401127908	2561696540		
30	2336301000	2492037518	2394820727		
60	2274035691	2471272649	2451785752		
120	2441883643	2336807460	2300848784		
240	2155189932	2372006445	2147939591		
**Area Ctrl T0**	2532301106				
**Time (min.)**	**% R1**	**% R2**	**% R3**	**Average %**	**Dv. St.**
0	100	100	100	100	0
2	99.33	95.17	104.39	99.63	4.62
15	98.52	94.82	102.02	98.45	3.60
30	92.26	98.41	95.37	95.35	3.07
60	92.45	97.59	97.64	95.89	2.98
120	99.27	92.28	90.86	94.14	5.50
240	96.36	93.67	89.52	93.18	3.44

Table S4. HRMS data of the Cathepsin B cleavage assays of compound **3**.

Pick Area Compound 3					
**Time (min.)**	**R1**	**R2**	**R3**		
2	4403219615	4862072070	5105619591		
15	4229359558	4353088991	4360050853		
30	3398761906	4724629672	4718152541		
60	3879030601	3762354302	4330486759		
120	3687637127	4010079166	3915519230		
240	3910246976	3616437185	3452333149		
**Area Ctrl T0**	4815156619				
**Time (min.)**	**% R1**	**% R2**	**% R3**	**Average %**	**Dv. St.**
0	100	100	100	100	0
2	91.44	100.97	106.03	99.48	7.41
15	87.83	90.40	90.55	89.60	1.53
30	70.58	98.12	97.99	88.90	15.86
60	80.56	78.14	89.93	82.88	6.23
120	76.58	83.28	81.32	80.39	3.44
240	81.21	75.11	71.70	76.00	4.82

Table S5. HRMS data of the Cathepsin B cleavage assays of compound **4**.

Pick Area Compound 4					
**Time (min.)**	**R1**	**R2**	**R3**		
2	2196999543	1914166293	2055582918		
15	2069742454	1814967509	1750554424		
30	1669428156	1433939083	1684007685		
60	1608958072	1376645397	1587750505		
120	1292719494	1270453224	1106255078		
240	1422045304	1136193233	1041356527		
**Area Ctrl T0**	2090842173.43				
**Time (min.)**	**% R1**	**% R2**	**% R3**	**Average %**	**Dv. St.**
0	100.00	100.00	100.00	100.00	0
2	105.08	91.55	98.31	98.31	6.76
15	98.99	86.81	83.72	89.84	8.07
30	79.84	68.58	80.54	76.32	6.71
60	76.95	65.84	75.94	72.91	6.14
120	61.83	60.76	52.91	58.50	4.87
240	68.01	54.34	49.81	57.39	9.48

8. pH stability assay – UHPLC-HRMS analysis

Table S6. HRMS data of the stability assays of compound **1-4** at pH 5.4 and 7.4. These data are the average of two replicates.

Stability compound 1 pH 5.4		
Time (min.)	Pick Area	%
0	2807425825	100.00
30	2626327362	93.60
120	2635474588	93.87
240	2632803938	93.78

Stability compound 1 pH 7.4		
Time (min.)	Pick Area	%
0	520093588	100.00
30	594837055	114.59
120	504490780	97.00
240	644070478	113.04

Stability compound 2 pH 5.4		
Time (min.)	Pick Area	%
0	1346583468	100.00
30	1346849861	92.49
120	1151098132	85.41
240	1168237690	86.76

Stability compound 2 pH 7.4		
Time (min.)	Pick Area	%
0	994309686	100.00
30	880034937	90.61
120	899353110	90.45
240	914665480	91.99

Stability compound 3 pH 5.4		
Time (min.)	Pick Area	%
0	2091363923	100.00
30	2013068626	96.15
120	2020516766	96.58
240	1829283018	89.91

Stability compound 3 pH 7.4		
Time (min.)	Pick Area	%
0	1631858280	100.00
30	1562621136	95.79
120	1565830997	95.93
240	1489445425	91.99

Stability compound 4 pH 5.4		
Time (min.)	Pick Area	%
0	997528676	100.00
30	905879117	90.83771
120	523303544	52.46537
240	470644998	47.47434

Stability compound 4 pH 7.4		
Time (min.)	Pick Area	%
0	152872508.8	100.00
30	144724364.1	94.66
120	147075775.5	96.21
240	145228883.4	95.00

.

9. Plasma stability assays

Protocol for full plasma assay: a stock solution of compounds 1-4 in DMSO was incubated in human (Anticoagulants: Na Heparin; Gender: Pooled; Cat. No: IPLANAH100ML; Lot: 37921; Supplier: Innovative Research) and rat (Strain: Sprague Dawley; Anticoagulants: Na Heparin; Cat. No: IGRTSDPLANAH100ML; Supplier: Innovative Research) plasma at a final concentration of 1 µM (final concentration of DMSO = 1 %). The samples were incubated at 37 °C with a shaking of 450 rpm. Aliquots of the samples were analyzed at time 0, 30 minutes, 2 and 4 hours by LC-MS/MS analysis.

Protocol for S9 fraction: a stock solution of compounds 1-4 in DMSO was incubated in human (male, pooled, commercially obtained from Xeno-tech lot: 2310104) and rat (male, pooled, commercially obtained from Xeno-tech (lot: 2210317)) S9 fraction at a final concentration of 1 µM (final concentration of DMSO = 0.5 %). The samples were incubated at 37 °C with a shaking of 450 rpm. Aliquots of the samples were analyzed at time 0, 30 and 90 minutes by LC-MS/MS analysis.

The areas of the peak of the compounds 1-4 in the LC chromatogram were used to evaluate stability profile. The percentage of residual compounds 1-4 was calculated as the ratio between the area of the peak of the analyte and the area of the internal standard.

$$\%\, \boldsymbol{residual\ compound} = \frac{\boldsymbol{Area\ analyte}}{\boldsymbol{Area\ internal\ standard}} \cdot \mathbf{100}$$

Table S7. LC-MS data of the stability assays in plasma extracts of compound **1**. These data are the average of two replicates.

**Stability assays compound 1**					
**Time (min.)**	**Plasma stability (matrix: rat) % remaining**	**Plasma stability (matrix: human) % remaining**	**Time (min.)**	**S9 stability (matrix: rat) % remaining**	**S9 stability (matrix: human) % remaining**
0	100.0	100.0	0	100.000	100.000
30	98.1	98.9	30	88.751	104.314
120	111.4	117.0	90	94.464	94.986
240	103.0	112.1	-	-	-

Table S8. LC-MS data of the stability assays in plasma extracts of compound **2**. These data are the average of two replicates.

**Stability assays compound 2**

Time (min.)	Plasma stability (matrix: rat) % remaining	Plasma stability (matrix: human) % remaining	Time (min.)	S9 stability (matrix: rat) % remaining	S9 stability (matrix: human) % remaining
0	100.0	100.0	0	100.000	100.000
30	92.1	102.5	30	72.843	133.447
120	99.0	104.0	90	61.754	95.232
240	103.3	93.9	-	-	-

Table S9. LC-MS data of the stability assays in plasma extracts of compound **3**. These data are the average of two replicates.

Stability assays compound 3					
**Time (min.)**	**Plasma stability (matrix: rat) % remaining**	**Plasma stability (matrix: human) % remaining**	**Time (min.)**	**S9 stability (matrix: rat) % remaining**	**S9 stability (matrix: human) % remaining**
0	100.0	100.00	0	100.000	100.000
30	122.7	101.95	30	86.541	133.447
120	112.8	98.33	90	42.650	95.232
240	118.1	77.06	-	-	-

Table S10. LC-MS data of the stability assays in plasma extracts of compound **4**. These data are the average of two replicates.

Stability assays compound 4					
**Time (min.)**	**Plasma stability (matrix: rat) % remaining**	**Plasma stability (matrix: human) % remaining**	**Time (min.)**	**S9 stability (matrix: rat) % remaining**	**S9 stability (matrix: human) % remaining**
0	100.0	100.0	0	100.000	100.000
30	96.1	100.8	30	81.090	106.613
120	103.8	105.8	90	81.171	79.748
240	108.0	107.1	-	-	-

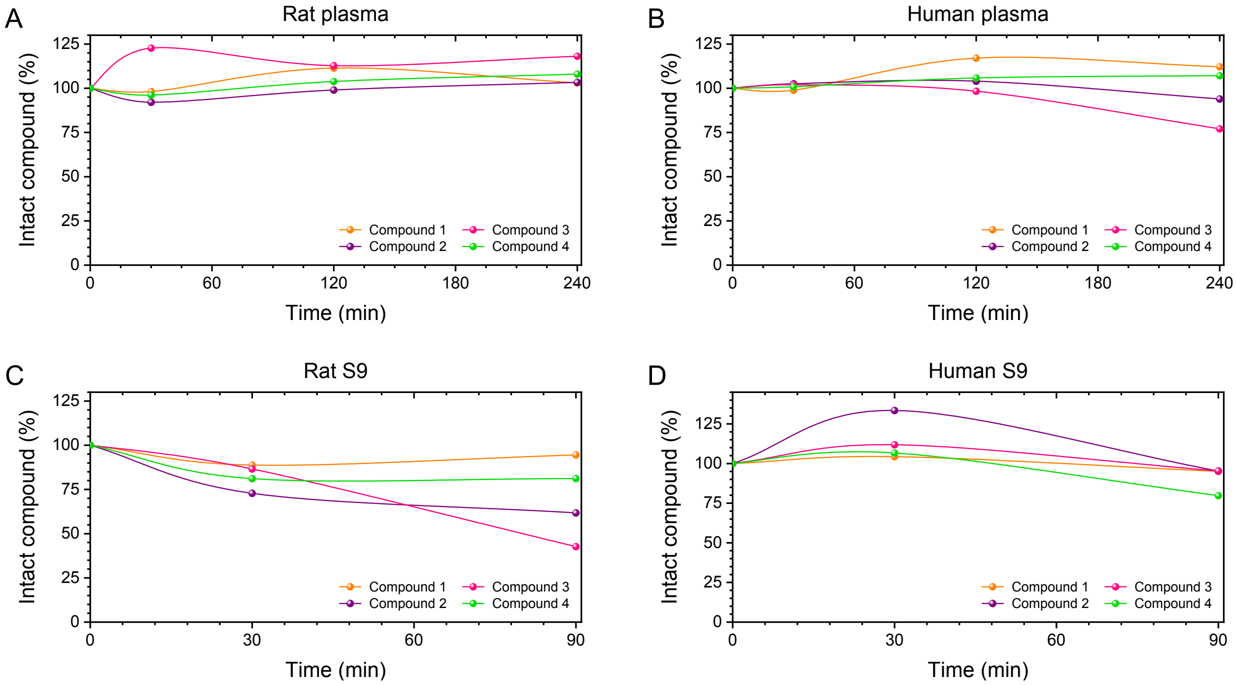

Figure S42. In vitro stability assays of compounds **1-4** in A) rat full plasma; B) human full plasma; C) rat S9 fraction; D) human S9 fraction.
